# Surface circumferential spinal cord recording in freely moving rodents

**DOI:** 10.1038/s41467-026-75128-z

**Published:** 2026-07-13

**Authors:** Salim El Hadwe, Ruben Ruiz-Mateos Serrano, George Psaltakis, Margaux Forner, Chaeyeon Lee, Sydney Swedick, Anton Banta, Xueer Zhang, Moleca Ghannam, Tawfique Hasan, Alejandro Carnicer-Lombarte, George G. Malliaras, Damiano G. Barone

**Affiliations:** 1 Electrical Engineering Division, Department of Engineering, University of Cambridge, Cambridge, UK; 2 Department of Clinical Neurosciences, University of Cambridge, Cambridge, UK; 3 Cambridge Graphene Centre, Department of Engineering, University of Cambridge, Cambridge, UK; 4 Department of Neurosurgery, Houston Methodist Hospital, Houston, TX USA; 5 Electrical and Computer Engineering Department, Rice University, Houston, TX USA; 6 Department of Biomedical Engineering, City University of Hong Kong, Hong Kong SAR, China; 7 Neuroengineering Initiative, Rice University, Houston, TX USA

**Keywords:** Biomedical engineering, Spine regulation and structure, Spinal cord diseases

## Abstract

Spinal cord injury affects over 2.5 million people worldwide, yet current neuroprosthetic strategies remain fragmented, addressing motor, sensory, or autonomic function in isolation. Here we show that a single ultrathin circumferential electrode array, conforming to the spinal cord without penetrating neural tissue, can simultaneously decode motor intent, classify sensory inputs, and discriminate visceral sensory inputs. In freely moving rats during short-term implantation (up to three days), deep learning decoders achieved robust motor intent decoding (R² = 0.97) by exploiting low-frequency spinal oscillations aligned with central pattern generator rhythms. The same interface classified eight sensory modalities with 94.4% accuracy. In acutely anaesthetized pigs, cross-species validation confirmed translational scalability and reliably distinguished visceral sensory inputs. Uniquely, the two-row electrode configuration resolved directional propagation within spinal tracts while electrode-dense one-row devices enabled high-precision intraspinal source localization. By consolidating motor, sensory, and visceral afferent decoding within a single conformal interface, this approach positions the spinal cord as a target for multifunctional neuroprosthetic interfacing, offering a path toward integrated restoration of physiological function after neurological injury.

## Abstract (web summary)

Spinal cord injury disrupts motor, sensory, and autonomic functions. Here, the authors demonstrate that a single ultrathin circumferential intradural electrode array can decode motor intent, classify sensory inputs, and discriminate visceral afferent signals across rodent and porcine models.

## Introduction

Spinal cord interfaces are rapidly transforming our ability to access^1^, modulate^2^, and decode^3^ activity from the central nervous system, enabling a new class of technologies aimed at understanding and restoring function after neurological disease or injury. Epidural spinal cord stimulation has demonstrated remarkable clinical success in restoring lost motor function^4,5^ and shows encouraging results for autonomic recovery^6–8^. These successes point the way towards comprehensive spinal neuroprosthetics that integrate motor, sensory, and autonomic function^6,9–11^.

Technological innovations have broadened the scope of spinal cord implants from epidural devices to intradural dorsal^12^ or ventral^13^ ones, as well as penetrating^3^ electrodes that can access deeper regions of the cord’s parenchyma for bidirectional interfacing. However, most existing approaches are either too invasive for clinical translation or remain constrained to limited spinal cord coverage, restraining their capacity to selectively and simultaneously engage the lateral and ventral structures that host critical circuitry essential for movement and other physiological regulatory tasks^14^.

Our prior work established the circumferential intradural recording concept: flexible thin-film arrays capable of 360-degree spinal cord coverage, with proof-of-concept recordings in anesthetized rodents, tissue-response assessment using dummy implants, and cadaveric human feasibility studies^15^. The present study extends this platform in three directions not previously demonstrated: stable recordings during short-term implantation (up to 3 days) in freely moving rats; high-fidelity motor intent extraction (*R*² = 0.97) and multimodal, plurisegmental sensory classification (94.4% accuracy) during naturalistic behavior; and cross-species scaling to porcine models with proof-of-concept visceral afferent discrimination. These advances position circumferential spinal recording as a functionally characterized platform for multidomain spinal decoding, identifying a path toward future closed-loop neuroprosthetic applications.

## Results

### Scalable circumferential designs safely validate across species

Our primary objective was to determine whether surface circumferential electrodes could capture spinal cord signals of sufficient quality and resolution to allow for their correlation with observed and evoked physiological functions. To this end, we first developed and tested device designs that could be easily and safely implanted to capture activity from the entire circumference of the spinal cord. We engineered narrow, ultrathin-film devices in one- or two-row configurations, allowing us to examine how electrode density and orientation influence coverage and directional resolution.

**Fig. 1:**
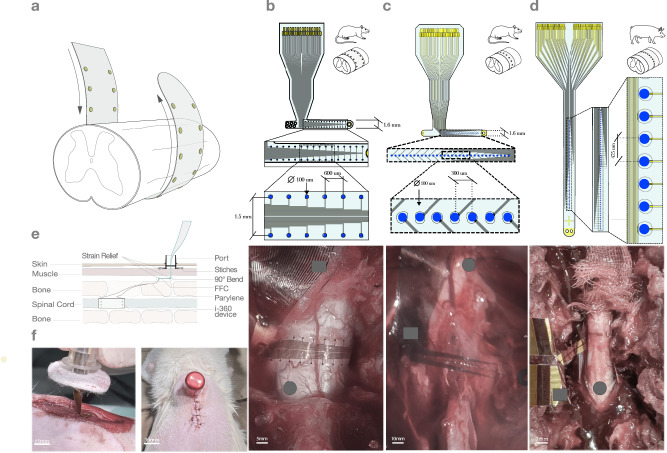
Circumferential spinal arrays enable safe cross-species implantation. **a** Concept illustration showing a circumferential electrode array wrapped around a spinal cord cross-section, providing 360-degree neural access. **b** Rodent dual-row device (32 electrodes, 600 μm spacing, 1.6 mm width) with surgical photograph demonstrating conformal placement around spinal cord, scale bar: 5 mm. **c** Rodent single-row device (32 electrodes, 300 μm spacing, 1.6 mm width) with implantation photograph showing device prior to implantation, scale bar: 10 mm. **d** Porcine single-row device (64 electrodes, 475 μm spacing) with surgical photograph demonstrating successful large-animal implantation, scale bar: 7 mm. Circles indicate the spinal cord, and Rectangles indicate the device. **e** Novel tunneling strategy with cross-sectional anatomy showing strain relief mechanisms, 90-degree bends, externalized flat flexible cable (FFC), and a subcutaneous port for recovery access. **f** Surgical photographs of the tunneling technique (left image, scale bar: 15 mm) and final device positioning (right image, scale bar: 30 mm) demonstrating successful externalization. i-360 intradural 360-degree device.

We initiated our investigation with rat models for their well-characterized spinal cord anatomy, sophisticated motor control range, and spinal cord dimensions^16^. Following a two-level laminectomy and durotomy, devices were wrapped around at the thoracolumbar junction (Fig. 1a and Supplementary Fig. 1). We deployed two-row devices containing 32 electrodes at 600 μm spacing (Fig. 1b) and one-row devices with 32 electrodes at 300 μm spacing (Fig. 1c), with overall widths of 1.6 mm and 1.6 mm, respectively. To assess scalability and translatability^17,18^, we extended the one-row design to large-animal models, fabricating porcine arrays with 64 electrodes at 475 μm spacing (Fig. 1d). Across species, devices were produced by photolithography on 4 μm parylene-C substrates, with gold electrodes coated in PEDOT: PSS to improve charge transfer and stability (Supplementary Fig. 2).

Because recovery studies require a stable connection setup that affords frequent sampling and is resistant to mechanical stress, we introduced a tunneling design incorporating a 90° bend and multiple strain relief points, integrated into a harboring port, to prevent breakage and disconnection (Fig. 1e, f). Using this approach, we implanted fifteen rodents (ten with one-row devices and five with two-row devices) and three pigs with one-row devices. In rodents, devices were maintained up to three days; in pigs, implantation was performed acutely under intravenous anesthesia. All animals resumed spontaneous ambulatory behavior following recovery from anesthesia, as documented in Supplementary Movie 1. Tissue-response characterization was conducted in our prior work^15^.

### Short-term freely moving recordings reveal locomotor signatures

**Fig. 2:**
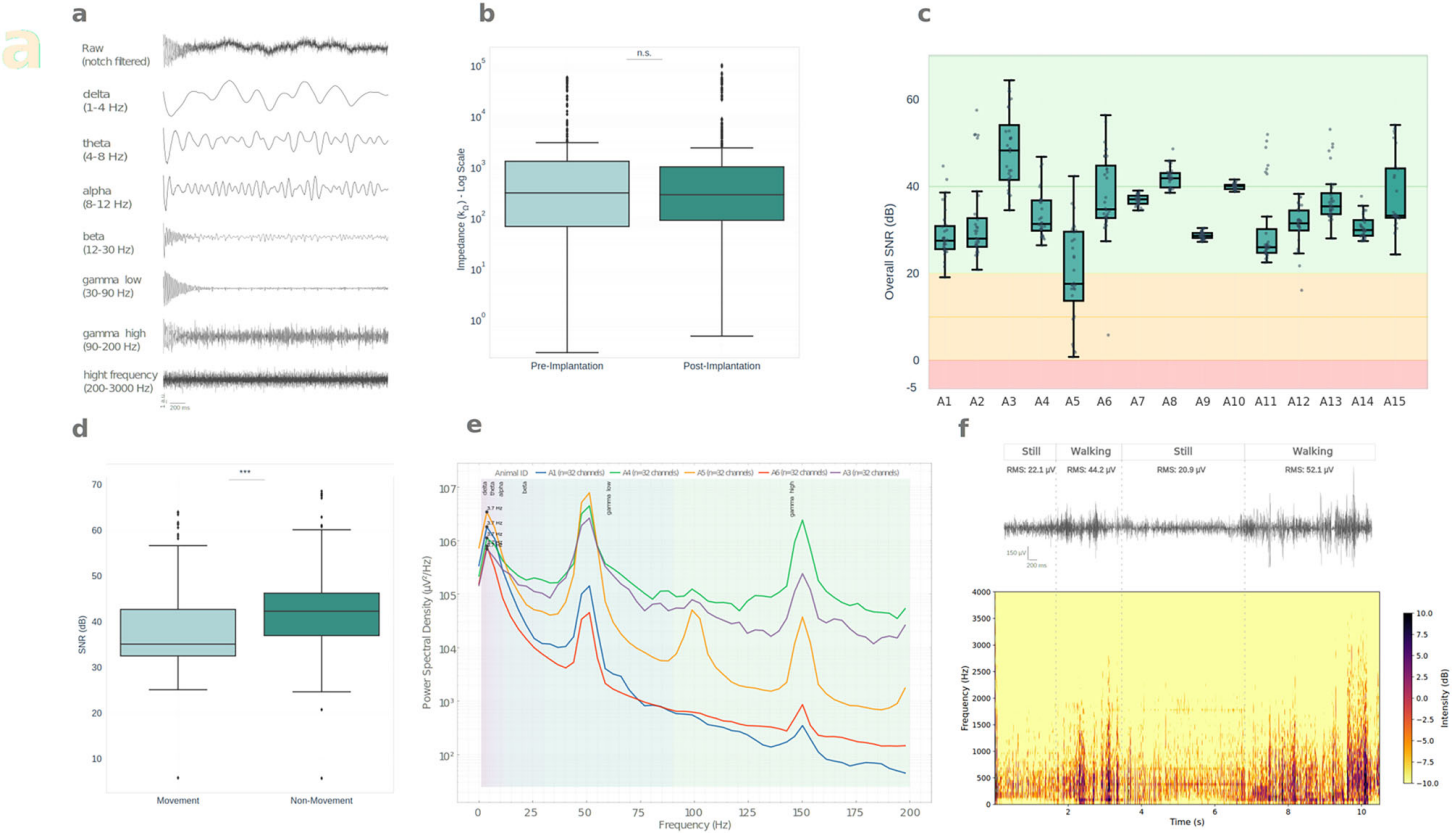
Recording from freely moving rats captures behaviorally modulated spinal activity. **a** Representative neural signals across frequency bands from a single freely moving rat, showing spectral diversity across analysis bands. Scale bars: 200 ms, 1 arbitrary unit (a.u.). Traces are representative of recordings. **b** Impedance stability pre- versus post-implantation. Box plots show median (center line), 25th–75th percentile (box bounds), 1.5× IQR (whiskers), and individual outliers (points). Data are presented as mean ± s.d. Pre-implantation: 4.2 ± 5.9 kΩ; post-implantation: 4.6 ± 6.0 kΩ. No significant difference was detected (two-sided paired *t* test, *t*(14) = 0.86, *P* = 0.412, Cohen’s *d* = 0.29; *n* = 15 animals, 32 channels per animal; normality of differences confirmed by Shapiro–Wilk test, W = 0.961, *P* = 0.792). n.s. not significant. **c** Signal-to-noise ratio distribution across all 15 animals showing consistent recording quality (overall mean ± s.d.: 33.7 ± 9.4 dB). Each box represents one animal’s electrode array (A1–A15; *n* = 32 channels per animal). Box plots show median (center line), 25th–75th percentile (box bounds), 1.5× IQR (whiskers), and individual outliers (points). Background shading indicates quality thresholds: green (> 40 dB), yellow (20–40 dB), red (< 20 dB). **d** Behavioral state modulation of SNR. SNR was significantly lower during movement than during rest (movement: 37.5 ± 9.7 dB; rest: 43.0 ± 8.8 dB; mean difference: −5.4 ± 10.0 dB; two-sided paired *t* test, *t*(159) = − 6.897, *P* = 1.19 × 10⁻¹⁰, Cohen’s *d* = −0.55, 95% CI: [−7.0, −3.9] dB; *n* = 160 electrode pairs across 5 animals, 32 channels per animal). Box plots show median (center line), 25th–75th percentile (box bounds), 1.5× IQR (whiskers), and individual outliers (points). Data are presented as mean ± s.d. ***P < 0.001. **e** Power spectral density profiles across subjects during locomotion, showing consistent 3.7 Hz locomotor peaks. Each colored trace represents one animal (A1–A5; *n* = 32 channels per animal, channel-averaged). Shaded frequency bands: delta (1–4 Hz), theta (4–8 Hz), alpha (8–12 Hz), beta (12–30 Hz), low gamma (30–90 Hz), high gamma (90–200 Hz). **f** Single-channel behavioral modulation. Top: high-frequency (300–2000 Hz) neural trace from a representative channel, with still and walking periods separated by dashed lines and annotated RMS amplitudes. Bottom: corresponding spectrogram showing spectral power modulation across behavioral states. RMS amplitude increased 2.24-fold during walking (44.2–52.1 μV) relative to rest (20.9–22.1 μV). Scale bars: 200 ms, 150 μV. Traces are representative of recordings from five animals with similar results. All box plot statistics derived from electrode pairs across independent animals. Data are presented as mean ± s.d. unless otherwise stated. n.s. not significant. ***P < 0.001.

We first assessed whether circumferential implants maintained stable performance during freely moving studies. Electrode impedances remained stable before and after implantation (4.2 ± 5.9 kΩ vs 4.6 ± 6.0 kΩ; *P* = 0.412, paired *t* test, *n* = 15), indicating that the implantation procedure, and recovery preserved device integrity and a stable tissue–electrode interface (Fig. 2b and Supplementary Figs. 3 and 4). Recordings from the fifteen freely moving rats consistently exhibited adequate signal quality (SNR: 33.7 ± 9.4 dB), confirming that the platform supports recovery of short-term spinal electrophysiology (Fig. 2c).

To determine whether these stable recordings could resolve physiologically relevant dynamics, we compared neural activity during rest and locomotion in freely moving rats. SNR systematically decreased during movement (37.5 ± 9.7 dB vs 43.0 ± 8.8 dB; *P* < 0.001, paired *t* test, *n* = 5), a finding presumably consistent with the recruitment of larger and more variable populations of neurons during active behaviors (Fig. 2d). Spectral analysis of locomotion epochs revealed robust peaks around 3.7 Hz across all animals, matching established central pattern generator rhythms that govern stepping movements (Fig. 2e)^19^.

Because spike sorting is unreliable in the moving spinal cord, where micromotion and motion artifacts confound unit isolation, we focused on local field potentials (LFPs) as a more stable representation of ensemble activity. RMS amplitude provided a measure of neural recruitment across behavioral states. Representative single-channel analyses demonstrated a 2.24-fold increase in RMS amplitude during walking compared to rest (44.2–52.1 μV vs 20.9–22.1 μV), confirming that the interface reliably captures locomotor-related neural dynamics. These findings demonstrate that our circumferential platform captures the fundamental neural signatures of spinal locomotor control, providing the stable, high-resolution recordings necessary for advanced functional decoding applications (Fig. 2a, f).

### Two-row arrays resolve directional signal flow within spinal circuits

Having established stable recording quality, we next leveraged the geometry and design of the two-row configuration to examine rostro–caudal signal propagation, a feature inaccessible to conventional planar arrays. We reasoned that electrode spacing along the spinal axis could reveal the direction of information flow, thereby exposing the functional organization of ascending and descending pathways recruited during an observed task.

**Fig. 3:**
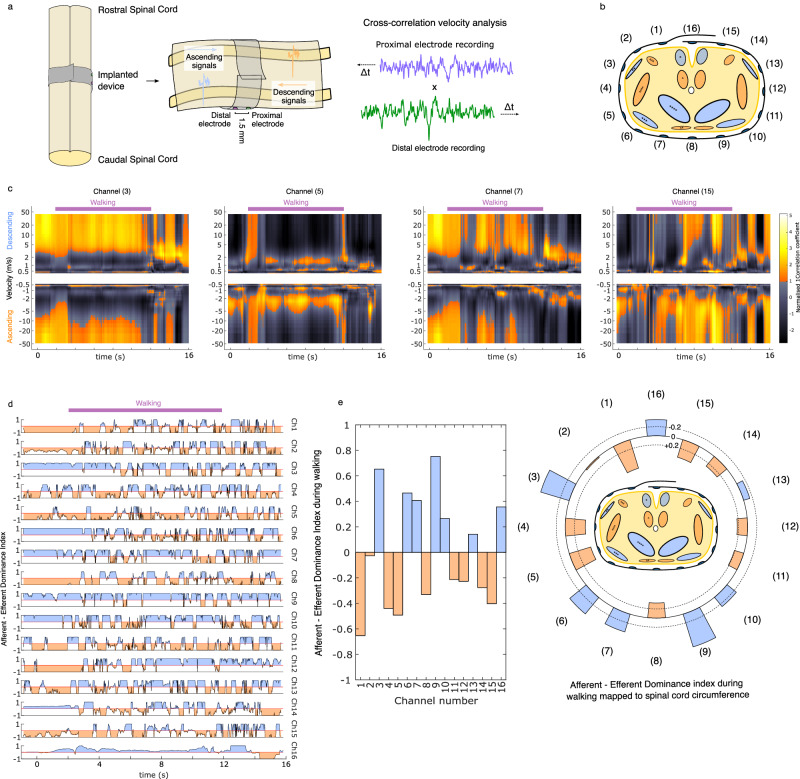
Two-row arrays reveal directional signal propagation. Data from a single representative rat across three independent locomotor sessions. **a** Schematic showing circumferential electrode placement for cross-correlation velocity analysis. Ascending (blue arrows) and descending (orange arrows) signals demonstrate directional propagation captured by the dual-row configuration. Distance between proximal and distal electrodes: 1.5 mm. **b** Electrode pairs positions (1–16) mapped around the spinal cord circumference showing anatomical orientation relative to major white matter tracts. **c** Cross-correlation velocity analysis from four representative channels during walking. Heatmaps display a temporal lag (−50 to +50 m/s), with positive velocities indicating rostral-to-caudal propagation and negative velocities indicating caudal-to-rostral propagation. Color intensity represents correlation strength. Walking periods marked by purple bars. Each channel displays a distinct velocity–time signature reflecting the anatomical position relative to spinal pathways. **d** Temporal evolution of afferent–efferent dominance index across all electrode pairs during walking. Traces range from −1 (entirely afferent, blue) to +1 (entirely efferent, orange), showing sustained directional preferences throughout locomotion epochs. **e** Left: Mean dominance index quantification across channels during walking. Positive values indicate efferent-dominant regions; negative values indicate afferent-dominant regions. Values represent a single recording session from one representative animal; no inferential statistics are shown. Equivalent analyses across all five two-row implanted animals are provided in Supplementary Fig. 24. **e** Right: Circumferential mapping of dominance indices onto spinal anatomy. Color intensity and segment size reflect directional bias strength, revealing spatially organized patterns corresponding to known tract locations. Tract assignments: *dorsal column; **dorsal spinocerebellar; ***ventral spinocerebellar; ****ventral spinothalamic; ^dorsal corticospinal; ^^rostral reticulospinal; ^^^rubrospinal; ^^^^dorsal reticulospinal.

We adapted cross-correlation velocity analyses from peripheral nerve studies^20^ and applied them to high-frequency band signals (200–3000 Hz) recorded from sixteen electrode pairs aligned rostro–caudally in a walking rat(Fig. 3a). The two-row configuration provided paired, circumferential coverage of the spinal cord cross-section (Fig. 3b), enabling simultaneous tracking of signal flow across dorsal, ventral, and lateral territories. This proof-of-concept analysis in a single rat implanted with a two-row device uncovered reproducible propagation signatures across three independent locomotor sessions, with velocity–time profiles that varied systematically with electrode location (Fig. 3c). Signal dynamics showed velocity-dependent modulation before, during, and after locomotion, with spatially heterogeneous patterns that likely reflected the proximity of each recording site to specific ascending or descending tracts (Fig. 3c).

To quantify directional bias, we computed an afferent–efferent dominance index (− 1 = afferent, +1 = efferent) across all electrode pairs during walking. This analysis revealed sustained directional preferences throughout locomotor epochs (Fig. 3d), which remained consistent across the three recording sessions. Grouped across electrodes, these preferences produced stable distributions (Fig. 3e, left). When projected onto the circumference of the cord, they formed spatially organized and laterally symmetric maps that corresponded closely to the described anatomical locations of ascending and descending tracts (Fig. 3e, right)^16^.

These findings reveal that surface recordings from two-row devices, combined with an appropriate sampling rate, can resolve the functional architecture of spinal information propagation, providing insight into how spinal circuits coordinate ascending sensory information with descending motor commands during natural movement. By functionally mapping this directional organization, circumferential recording establishes a foundation for targeted interventions that can selectively engage specific pathways to restore lost function due to injury.

### Decoding motor intent with delta-band dominance

**Fig. 4:**
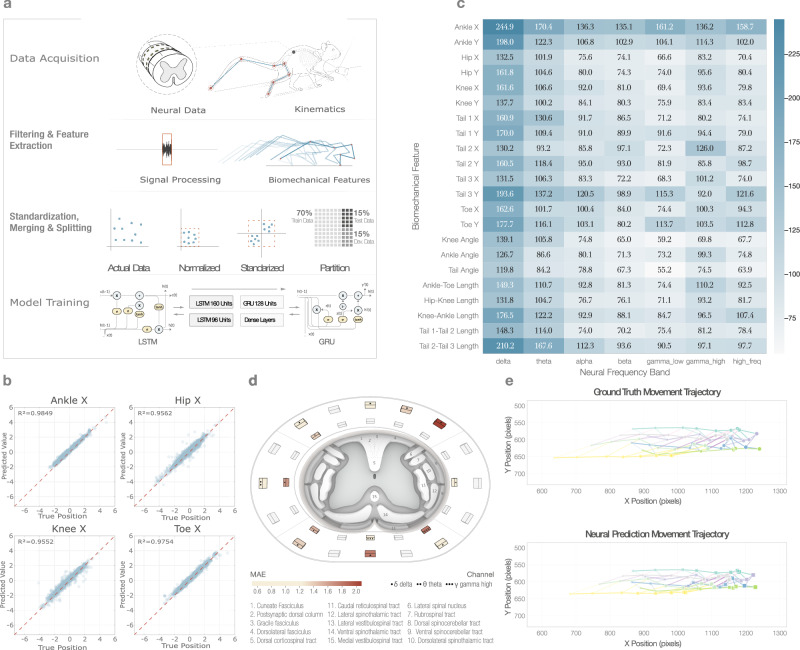
LSTM–GRU model extracts motor intent from circumferential recordings. **a** Experimental and computational workflow. Neural data underwent filtering and feature extraction (RMS and mean amplitude across seven frequency bands) before temporal alignment with DeepLabCut-tracked kinematics (7 anatomical landmarks, 15 calculated features). Data were partitioned (70% training, 15% validation, 15% test) and input to a bidirectional LSTM–GRU architecture predicting 22 biomechanical features. **b** Decoding performance for key landmarks on held-out test data. Scatter plots show predicted versus ground truth values for ankle X (*R*² = 0.988, 95% CI: [0.986, 0.989]), hip X (*R*² = 0.965, 95% CI: [0.960, 0.970]), knee X (*R*² = 0.959, 95% CI: [0.952, 0.965]), and toe X (*R*² = 0.978, 95% CI: [0.975, 0.981]). The red dashed line indicates perfect correlation. Each point represents one test-set time sample. **c** Frequency band contribution analysis. The heatmap shows the percentage MAE increase when each frequency band is systematically removed for all 22 biomechanical features. Color intensity represents the magnitude of MAE increase (%), with warmer colors indicating greater contribution. Delta band (1–4 Hz) shows the highest contribution across most features. **d** Spatial organization of predictive information. Circumferential spinal cord map showing top 20 most important channel–frequency combinations. Color intensity represents the percentage MAE increase upon ablation of that channel–frequency combination. Channel numbers (1–32) indicate electrode positions around the cord circumference. **e** Temporal trajectory validation. Representative locomotion epoch showing ground truth (top) and neural decoding (bottom) movement trajectories for *X*–*Y* coordinates. Traces are representative of recordings from all five animals with similar results. All 22 features on held-out test data showed *R*² =  0.919–0.988 (95% CIs reported in Supplementary Fig. 6) and MAE = 0.076–0.150 (95% CIs reported in Supplementary Fig. 6). Two-sided Pearson correlation exceeded *r* > 0.958 across all features (*P* < 0.001; *n* = 3441 time samples from five animals).

Having established that circumferential arrays capture stable locomotor signatures and resolve rostro–caudal propagation, we next investigated whether these signals could support precise motor intent decoding when coupled with gait kinematics. To that end, we trained a bidirectional recurrent neural network on synchronized neural and kinematic recordings from five freely moving rats, implanted with the two-row alterations of our device (Fig. 4a and Supplementary Figs. 5–6). Input to the network features consisted of band-limited average and RMS amplitudes extracted from the 32-channel recordings of each of the five rats. While output consisted of the paired to recordings tracked and calculated biomechanical trajectories derived from markerless kinematics tracking with DeepLabCut^21^.

The LSTM–GRU network architecture, trained on pooled data from all five rats, achieved robust decoding across the 22 biomechanical features when tested on held-out data. Ankle position achieved the highest accuracy (*R*² = 0.99, MAE = 0.09), representing the best-performing feature. All features exhibited strong correlations with ground truth trajectories (Pearson *r *> 0.95, *P* < 0.001, *n* = 5), as shown in scatter plots and trajectory reconstructions (Fig. 4b, e and Supplementary Figs. 7–9).

To identify the neural basis of this performance, we systematically ablated input frequency bands from the model. Prediction accuracy was most dependent on the delta band (1–4 Hz), removal of which increased MAE by 19.3%, followed by theta (13.8%) and high gamma (11.7%) (Fig. 4c and Supplementary Fig. 10). This frequency dependence aligns with the 3.7 Hz spectral peak observed during locomotion (Fig. 2e), indicating that the decoder leverages the same low-frequency spinal oscillations that underlie central pattern generator activity to extract rhythmic motor intent. To complement ablation, we applied Shapley Additive exPlanations (SHAP), a model-agnostic interpretability method that assigns importance scores to individual features based on their marginal contribution to predictions. SHAP analysis independently confirmed the critical role of delta RMS features, with recent time windows weighted most strongly (Supplementary Figs. 11–12). Unlike ablation, which removes entire frequency bands, SHAP provides fine-grained attribution, further validating that low-frequency inputs carry disproportionate predictive weight.

Per electrode ablation analysis revealed a non-uniform distribution of predictive information across the circumferential array (Fig. 4d). The top 20 most informative electrode–frequency combinations clustered predominantly in the delta band, with additional contributions from theta and high gamma channels. Ipsilateral electrodes relative to the tracked hindlimb showed the greatest influence. This spatial distribution suggests that decoding accuracy arises from structured, anatomically grounded sources, likely reflecting the organization of lumbar motor pools and their descending inputs.

To externally validate overall model performance, we benchmarked the LSTM–GRU against both classical decoders and alternative machine learning approaches (Supplementary Fig. 13). The neural network consistently outperformed all comparators, achieving the lowest mean absolute error (MAE = 0.1166) compared with Kalman filters (MAE = 0.75), Wiener filters (MAE = 0.56), Random Forest (MAE = 0.24), and XGBoost (MAE = 0.35). Significantly, the identification of low-frequency dominance was not limited to the neural network. Feature importance analyses from Random Forest and XGBoost (the second and third-best performers) again highlighted the delta and theta bands as the strongest predictors, while a mutual information analysis, an independent, non-machine learning method, confirmed that low-frequency features showed the strongest dependencies with biomechanical outputs (Supplementary Fig. 13).

Across methods and models, the same picture emerged: low-frequency spinal rhythms dominate the neural code for movement in a model that was trained on combined data from five rats. Convergent evidence from ablation, SHAP, feature importance, and mutual information strongly suggests that delta oscillations, resonating at the 3.7 Hz rhythm of locomotion, constitute the shared language through which spinal circuits broadcast rhythmic motor intent, and through which decoders can recover it with the highest fidelity.

### Neural decoding differentiates sensory and autonomic responses across species

Having demonstrated precise motor decoding, we next asked whether circumferential recordings could also resolve physiologically evoked input, including sensory and autonomic signals, both crucial for enabling closed-loop prosthetics targeting patients with lost functions.

**Fig. 5:**
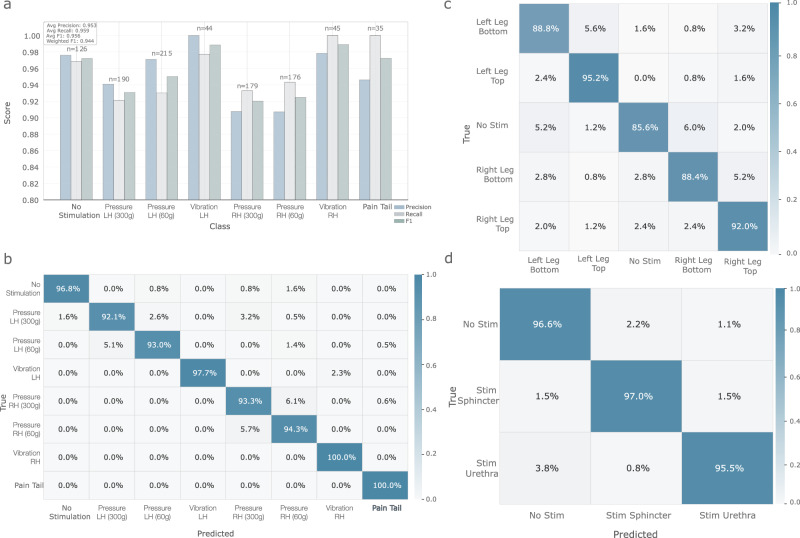
Neural decoding differentiates sensory and visceral afferent responses across species. **a** Eight-class sensory stimulus classification performance in freely moving rodents (*n* = 4 animals). Bar plots show precision (dark gray), recall (mid gray), and F1 score (light gray) for each stimulus class: bilateral pressure (60 g, 300 g), vibration (183.3 Hz), and tail nociception. Per-class test set sample sizes: no stimulation *n* = 126, pressure left 300 g *n* = 190, pressure left 60 g *n* = 215, vibration left *n* = 44, pressure right 300 g *n* = 179, pressure right 60 g *n* = 176, vibration right *n* = 45, tail pain *n *= 35; total test samples *n* = 1010. Overall weighted F1 = 0.944; macro-averaged precision = 0.953, recall = 0.959, F1 = 0.956. Cohen’s *κ* = 0.93; macro-averaged AUROC = 0.998. Overall classification accuracy was 94.36% versus a chance baseline of 21.29% (chance defined as uniform random classification across 8 classes). **b** Confusion matrix for rodent eight-class sensory classification. Color scale represents normalized classification rate per true class (0 = white, 1 = dark blue). Diagonal entries indicate correct classification rates. Systematic off-diagonal errors occur between pressure intensities applied to the same limb, consistent with overlapping somatotopic representation. **c** Cross-species validation in porcine models. Five-class bilateral leg stimulation (left leg bottom, left leg top, no stimulation, right leg bottom, right leg top) in anesthetized pigs, presented as proof-of-concept demonstration (*n* = 2 pigs, *n* = 1254 total test samples, *n* = 251 per stimulation class, *n* = 250 no stimulation class). Macro-averaged F1 = 0.90; per-class F1 range: 0.88–0.93. Color scale as in (**b**). **d** Visceral afferent classification in a single anesthetized pig, presented as proof-of-concept demonstration (*n* = 1 pig, *n* = 533 total test samples; no stimulation *n* = 267, rectal sphincter distension *n* = 133, urethral sphincter distension *n* = 133). Three-class classification of rest, urethral sphincter distension, and rectal sphincter distension achieved overall accuracy = 0.96; macro-averaged F1 = 0.96 (per-class F1: no stimulation = 0.97, sphincter = 0.96, urethra = 0.96). Color scale as in (**b**).

In recovered rodents, the same LSTM–GRU architecture reliably classified multimodal, plurisegmental, sensory stimuli (Fig. 5a). Sensory stimuli were delivered while animals were freely ambulatory, requiring discrimination of evoked responses amid ongoing locomotor activity. On held-out test data, the model achieved 94.4% accuracy, far exceeding baseline (21.3%, *P* < 0.001, *n* = 4), with excellent calibration (Cohen’s *κ* = 0.93, AUROC = 0.99). Performance varied modestly by modality: vibrational and nociceptive inputs were decoded with near-perfect fidelity (F1 > 0.97), whereas pressure stimuli yielded slightly lower but still robust values (F1 = 0.92–0.95). Confusion matrix analysis reveals that most errors originated from different pressure intensities applied to the same limb (Fig. 5b and Supplementary Figs. 14–16), consistent with overlapping neural recruitment within the same or adjacent somatotopic territories of the spinal cord.

To probe feasibility in larger anatomical models, we extended the approach to anesthetized porcine models as a proof-of-concept demonstration. Five-class classification of bilateral leg stimulation achieved a macro-averaged F1 score of 0.90 (range: 0.86–0.93, *n* = 2), confirming that sensory decoding generalizes from rodents to large animals (Fig. 5c and Supplementary Figs. 17–19). Furthermore, decoding accuracy remained consistent across stimulation sites in these experiments (*n* = 2), demonstrating the feasibility of capturing inputs distributed across the spinal circumference in a larger anatomical model that is scalable to human anatomy.

We then turned to visceral afferent decoding using an independent model, trained separately from the sensory classification network on a distinct experimental paradigm, which provides autonomically relevant information for bladder and bowel function monitoring, as an exploratory proof-of-concept demonstration. In a single porcine experiment, an independent network achieved near-perfect classification of rest, urethral sphincter distension, and rectal sphincter distension (F1 ≥ 0.97, *n* = 1), with virtually no cross-class confusion (Fig. 5d and Supplementary Figs. 20–22). These results demonstrate that even subtle physiological states, previously inaccessible to spinal surface recordings, can be discriminated with high fidelity.

Together, these findings establish that access to circumferential spinal recordings enables interrogation of functions that go beyond motor intent to encompass the afferent domains of spinal processing. By resolving sensory input and monitoring autonomic states with high accuracy across species, this platform lays the groundwork for fully bidirectional spinal neuroprosthetics that integrate motor, sensory, and autonomic control within a single interface.

### Precise intraspinal source localization via circumferential recording

A defining requirement for future spinal neuroprosthetics is not only the ability to record and decode spinal activity, but also to localize the origin of such activity within the cord. Accurate source localization would enable retro-engineered targeted stimulation of specific tracts while avoiding off-target activation, a prerequisite for the selective recruitment of motor, sensory, or autonomic pathways. We therefore evaluated whether circumferential, surface recordings could resolve the spatial origin of evoked intraspinal activity.

**Fig. 6:**
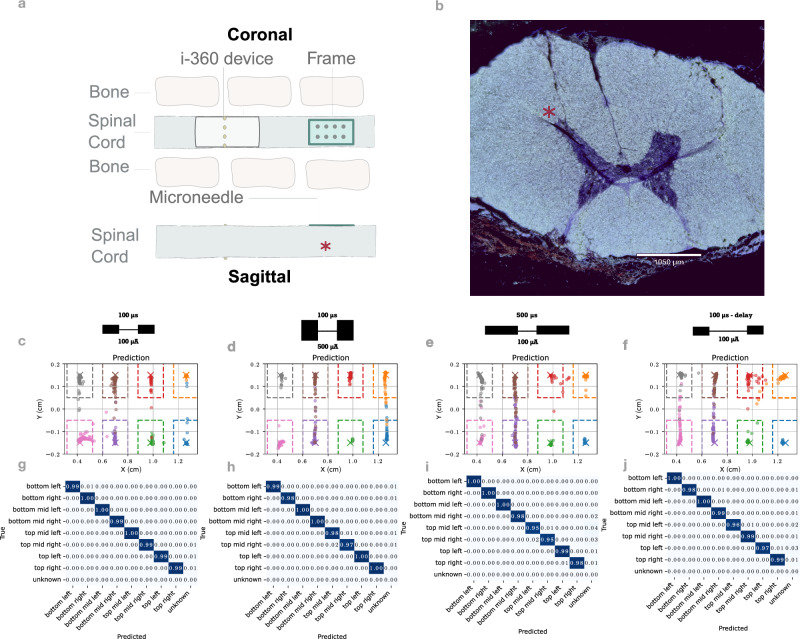
Circumferential arrays enable precise intraspinal source localization. **a** Experimental setup showing the circumferential intradural device (i-360; intradural 360-degree electrode array) placed around the spinal cord with a custom polyimide frame containing eight guide holes for controlled microneedle electrode insertion positioned caudally. Electrical stimuli were delivered to eight predefined sites across the spinal cord cross-section, with neural signals recorded via the circumferential interface positioned rostral to the stimulation region. Red asterisk indicates stimulation site. **b** Representative histological cross-section of porcine spinal cord confirming accurate microneedle placement at the intended stimulation site. This image is representative of a single experimental preparation (*n* = 1 animal); histological verification was performed once per animal. Scale bar: 1050 μm. Red asterisk indicates needle track. **c**–**f** Spatial prediction visualization across four stimulation conditions in a single anesthetized pig (*n* = 1 animal, one stimulation trial per condition): **c** control condition (100 μA, 100 μs biphasic); **d** high-amplitude condition (500 μA, 100 μs); **e** extended-duration condition (100 μA, 500 μs); **f** interphase-delay condition (100 μA, 100 μs, 500 μs interphase delay). Each colored symbol represents model-predicted coordinates for one time sample at a given stimulation site; eight colors correspond to eight stimulation sites as defined in the figure key (bottom left, bottom right, bottom mid left, bottom mid right, top mid left, top mid right, top left, top right). Cross markers (×) indicate true stimulation coordinates. Dashed boxes indicate 1 mm × 1 mm classification regions centered on each stimulation site. **g**–**j** Confusion matrices corresponding to (**c**–**f**), respectively, showing normalized classification performance when continuous coordinate predictions are discretized to spatial regions. Color scale represents normalized classification rate per true class (white = 0, dark blue = 1.00). Diagonal entries indicate correct classification rates. Classification accuracy across the four conditions: **g** 99%, **h** 98%, **i** 97%, **j** 97%. Accuracy was computed as the proportion of test-set time samples assigned to the correct 1 mm × 1 mm spatial region. results represent a proof-of-concept experiment (*n* = 1 animal). Misclassifications were confined to spatially adjacent sites within the same cord quadrant. Stimulation waveforms are shown above each panel pair.

To this end, we systematically delivered electrical stimuli at eight predefined sites, caudally, at different distances and lateralities to the center of the implanted device (Fig. 6a). Stimuli were applied using fine microneedle electrodes inserted at a fixed depth in the spinal cord’s parenchyma, with post hoc analysis histological assessment confirming accurate placement at the intended locations and depth (Fig. 6b). To ensure that recorded signals represented true neural responses rather than direct stimulation artifacts, we manually removed stimulation transients from the raw recordings before analysis, isolating physiologically relevant waveforms for decoding (Supplementary Fig. 23). We trained a similar LSTM–GRU model to predict stimulation site coordinates from the recorded responses. Across four independent experimental conditions in a single porcine preparation (*n* = 1), the system achieved 95–99% classification accuracy (Fig. 6c–f). Confusion matrix analysis revealed near-perfect discrimination between spatially distinct sites, with errors confined to high-amplitude stimulation, or adjacent locations within the same quadrant of the cord, and to sites with less distance between the source and the implant (Fig. 6g–j). This pattern of results suggests that circumferential recordings capture rich, topographically organized features of neural activation that can be exploited to resolve source localization at millimeter scales.

The ability to discriminate between closely neighboring sites demonstrates that the circumferential geometry provides complementary perspectives on activation spread across the dorsal, lateral, and ventral surfaces. By sampling simultaneously from all territories, the interface captures spatially distributed activation patterns that enable reconstruction of a spatial fingerprint for intraspinal sources; a capability that has not been demonstrated using single-plane or epidural devices.

These findings show that circumferential arrays extend beyond decoding to provide high-precision maps of intraspinal activity. This capacity is pivotal for translation, offering a path to closed-loop neuromodulation strategies in which stimulation is guided in real time by the precise location of activity within the cord.

## Discussion

We demonstrate that a single, ultrathin circumferential intradural device can decode motor, sensory, and autonomic signals while resolving spatial sources, all achieved without penetrating or disrupting neural tissue. This approach positions the spinal cord as a target for bidirectional, multifunctional neural interfacing^1^.

The clinical imperative for such integration is profound: spinal cord injury affects 2.5 million people worldwide, with current therapeutic approaches remaining functionally fragmented^22–24^. Epidural stimulation can restore some motor function but provides limited sensory feedback; brain-computer interfaces offer motor intent decoding but require separate peripheral systems for command execution. Moreover, autonomic dysfunction, disrupting cardiovascular, respiratory, bladder, and bowel control, represents one of the most debilitating consequences that remains largely unaddressed by existing neuroprosthetics^25–29^. This fragmentation necessitates multiple surgical procedures, increases infection risk, exposes unaffected brain tissue, and complicates system integration for comprehensive functional recovery.

By targeting the thoracolumbar cord, where descending supraspinal commands, lumbar CPGs, and other propriospinal circuits converge^30,31^ we established that circumferential surface recordings can capture physiologically relevant spinal signals during natural movement. The unique two-row configuration enabled device-level mapping of rostral–caudal signal propagation across the spinal circumference. Recent work^32^ demonstrated detection of propagating neural spikes along dorsal planar arrays in freely moving rodents. Our circumferential configuration extends this by sampling directional propagation across dorsal, lateral, and ventral territories simultaneously, revealing spatially organized patterns corresponding to tract anatomy. Building on these recordings, we achieved motor intent decoding in freely moving rats. Surface recordings at the thoracolumbar level likely reflect ensemble activity from multiple co-active neuronal populations, including segmental CPG circuitry, descending corticospinal and reticulospinal tracts, propriospinal projections, and sensory integration networks; our decoding approach leverages this compound signal for functional decoding purposes. Ablation analysis of our decoder revealed that removing the delta band (1–4 Hz) resulted in the biggest performance drop. This mechanistic insight is consistent with afferent–efferent dominance patterns of established V0/V1 interneuron CPG architecture and confirms that low-frequency spinal oscillations serve as primary carriers of rhythmic locomotor intent^3,33^. Our reported decoding capabilities are comparable to those reported with intraspinal electrodes^3^, achieved using a non-penetrating, circumferential interface, presumably due to the nature of the coverage. Most importantly, for both directional mapping and locomotion decoding, granular channel analysis revealed spatial patterns that aligned with known spinal cord anatomy when mapped to the circumferential geometry^16^. The per electrode ablation and importance analyses (Fig. 4d and Supplementary Figs. 10–11) demonstrate that the most informative features are distributed around the cord circumference, with predominant contributions from ventral and ipsilateral electrodes. This spatial distribution underscores the functional necessity of circumferential coverage, as a dorsal-only configuration would exclude substantial predictive information from non-dorsal territories.

Our platform’s versatility extended beyond motor control to include sensory and autonomic function decoding. In awake rats, we achieved eight-class sensory discrimination (pressure, vibration, and nociception across both hindlimbs and tail) with 94.36% accuracy. Validation in pig models showed promising scaling of sensory decoding capabilities.

Visceral afferent signal discrimination in pigs, while demonstrated in a single proof-of-concept experiment, suggests that surface electrodes may provide access to autonomic-relevant sensory pathways; a capability that, if validated in larger cohorts, could address a critical gap in current neurorehabilitation practice. Our proof-of-concept demonstrations in porcine models suggest that the principles of circumferential intradural recording may scale to larger, clinically relevant dimensions. The recovery and scaled-up device remained feasible for implantation and maintained signal discrimination across species, supporting its potential for scaling to larger anatomical dimensions. Its ultrathin, planar design and viscoelastic properties allowed it to conform to neural tissue with minimal disruption, respect surface blood vessels, preserve low impedance, and tolerate micromotion; advantages that penetrating arrays struggle to offer^3,33–35^.

We postulate that this circumferential configuration, which mirrors the organization of spinal cord tracts, allows wide access to diverse functional pathways through a single multichannel implant.

This anatomical complementarity represents an alternative approach to current brain-centric approaches toward targeting the affected organ itself. Most severe spinal cord injury patients undergo spinal surgery (e.g, decompression and/or stabilization) as part of acute injury management, making rostral spinal sampling a logical extension of these clinically necessary procedures rather than additional brain interventions in unaffected tissue^25,36^. The spinal cord offers distinct advantages as a neuroprosthetic target due to its role as a convergence point for multiple functional systems. Whereas cortical motor functions are anatomically distributed across the primary motor cortex, supplementary motor areas, basal ganglia, and cerebellum, the spinal cord represents the site where these distributed commands converge as an integrated motor intent^37,38^. This convergence provides direct access to processed movement commands while simultaneously offering proximity to ascending sensory pathways and descending autonomic control circuits^39^. Rehabilitation applications, such devices offer potential for acute post-injury assessment and recovery monitoring in patients with spinal cord injuries. Similarly, the device could be instrumentalized for intraoperative neuromonitoring during intradural spinal procedures^1^.

Finally, porcine intraspinal source-localization experiments demonstrated discrimination of eight discrete multi-setting stimulation sites across the spinal cross-section. This spatial mapping capability provides insight into the origins of intraspinal neural activity. In future applications, such spatial information could potentially guide targeted stimulation of defined parenchymal targets.

Advanced approaches, such as temporal interference, could exploit this spatial mapping to selectively activate desired pathways while minimizing off-target recruitment, thereby enabling a new generation of closed-loop, circuit-specific spinal neuroprosthetics^40–42^.

Challenges remain: Reliable and consistent implantation, as well as chronic biocompatibility, along with optimized dural-closure techniques and materials testing to prevent delamination and reduce foreign-body reactions, are prerequisites for translational fruition^43–45^. Post-injury changes present additional complexity, as altered receptor expression and synaptic remodeling emerge within weeks to months following SCI, potentially requiring adaptive decoding algorithms to maintain performance as neural representations evolve^46,47^. Importantly, circumferential recording targets preserved cord segments rostral or caudal to the lesion rather than the injury epicenter, where edema and scarring are most severe; clinical evidence from intradural duroplasty procedures confirms surgical feasibility even in the acute post-injury period^48^. Moreover, individual anatomical variability in spinal cord anatomy and level of injury may also affect electrode positioning and signal interpretation across patients. In addition, real-time translation requires miniaturized electronics, wireless telemetry, dedicated online decoding hardware, and causal, low-latency architectures for zero-latency closed-loop operation^49,50^. Finally, regulatory approval, along with human-scale safety validation, is essential^51,52^.

Our intradural device advances spinal neuroprosthetics by enabling direct, high-resolution access to the spinal cord’s intrinsic circuitry. Its ability to decode motor, sensory, autonomic, and spatial signals from a single, conformal interface provides a unified alternative to functionally fragmented cortical, peripheral, and epidural approaches. These findings establish a foundation for closed-loop neuromodulation strategies aimed at restoring integrated spinal function following cord insult.

## Methods

### Device design and fabrication

Thin-film circumferential intradural arrays were produced as 32-channel linear electrodes for rodents and 64-channel arrays for pigs, with minimized interconnect length, a reinforced gold ring for 7-0 polypropylene suture guidance, and gold alignment marks to ensure consistent placement. Device lengths were 8.78 mm (small rat), 9.33 mm (large rat), 27.12 mm (small pig), 30.12 mm (medium pig), and 33.12 mm (large pig), and each electrode site measured 0.1 × 0.1 mm.

Parylene-C substrates (2 μm for rodents; 3.5 μm for pigs) were deposited on 4″ silicon wafers (PDS 2010 Labcoter 2). Wafers were cleaned (acetone, IPA), spin-coated with AZ nLOF 2035 photoresist (~ 4 μm at 1500 rpm, 1000 rpm/s), soft-baked (110 °C, 2 min), UV-exposed (150 mJ/cm²), post-baked (110 °C, 60 s), and developed in AZ 726 MIF for ~2 min, then rinsed with distilled water and dried under nitrogen.

Oxygen plasma activation (100 W, 0.6 mbar O₂, 60 s; Plasma Pro 80 RIE) preceded e-beam evaporation of 5 nm Ti/100 nm Au ( < 6 × 10⁻⁶ Torr) and ≥2 h acetone liftoff with IPA rinse. A second parylene-C passivation (2 μm rodent; 3.5 μm porcine) was deposited, and device outlines were patterned in AZ 10XT resist (rodent: 500 rpm→3000 rpm; pig: 500 rpm→1000 rpm), UV-exposed (380 mJ/cm² rodent; 560  mJ/cm² pig), developed in AZ 726 MIF (6 min rodent; 10 min pig), and etched by RIE at 500 nm/min. Passivation windows over electrodes and tracks were defined by a further AZ resist step (500 rpm→3000 rpm, UV 380 mJ/cm², develop 6 min) and RIE at 200 nm/min. Electrode sites were coated with PEDOT:PSS (CLEVIOS PH 1000 + ethylene glycol + DBSA; spin 1500 rpm, bake 120 °C 1 h), then patterned in AZ 5214E (3000 rpm, UV 80 mJ/cm² + 500 mJ/cm² flood, develop 30 s) and etched (RIE 100 nm/min). Devices were bonded to 33-channel, 0.3 mm-pitch flat-flex cables using a Fineplacer and 5 μm anisotropic conductive film.

### Impedance measurements

Impedance measurements were obtained at 1 kHz using an AutoLAB potentiostat in phosphate-buffered saline, with a platinum counter/reference electrode. Post-implantation measurement(s) were done using the Intan RHX hardware.

### In vivo experiment

Surgical procedures were conducted under sterile conditions in compliance with the UK Animals (Scientific Procedures) Act 1986 and approved by the Animal Welfare and Ethical Review Body at the University of Cambridge. Studies were performed under Project License No. PP6076787 (large animals) and PFF2068BC (rodents), with procedures conducted under Personal License No. I54111393.Implantation duration was limited to three days by UK Home Office project license constraints (PFF2068BC), which precluded single housing; group-housed animals with externalized connectors were susceptible to cage-mate interference and thus unnecessary harm. Porcine implantations were performed under terminal anesthesia as acute proof-of-concept demonstrations of anatomical scalability.

### Animal preparation

Adult female Sprague–Dawley rats (*n* = 15; 250–300 g) were anesthetized with isoflurane (5% induction, 2–3% maintenance), received buprenorphine (0.05 mg/kg s.c.), had corneas lubricated, and core temperature maintained at 37–38 °C. Animals were placed in a stereotaxic frame with neutral cervical spine alignment and abdomen unobstructed.

Large white pigs (*n* = 3) were fasted for 12 h (water ad libitum), sedated with ketamine/xylazine/atropine, intubated, and maintained on isoflurane (1.6–2.0%). Core temperature was held at 38.6 ± 0.5 °C, Lactated Ringer’s (7 mL/kg/h IV) provided fluid support, and buprenorphine (0.02 mg/kg IM) was given 15 min pre-incision. For electrophysiological testing, anesthesia was transitioned to propofol (2–3 mg/kg IV bolus, 0.1–0.2 mg/kg/min infusion).

### Device implantation (rodents)

Under sterile conditions, a 4-cm midline thoracolumbar incision and muscle dissection exposed three spinal levels. Devices were implanted at T11–T12 vertebral level, providing access to lumbar motor circuitry while avoiding the increased diameter of spinal cord enlargements. Device widths (1.6 mm for two-row, 1.6 mm for one-row) were designed to fit between adjacent dorsal root exit zones. A two-level laminectomy and pedicle resection opened the ventral epidural plane, into which a 7-0 polypropylene suture guided the array circumferentially around the cord. The device was rotated into orientation, tacked dorsally with silicone sealant (Kwik-Sil), and a grounding wire was affixed to the paravertebral muscle. The flexible flat cable was externalized via a vascular port into a subcutaneous pocket with controlled slack and dual 90° bends to relieve strain; the port was sutured to paraspinal musculature, and the skin was closed.

### Device implantation (pigs)

A 12-cm L1–L2 incision (fluoroscopically confirmed) and muscle dissection exposed posterior elements and facets. Two-level laminectomy and flavectomy exposed the dura; durotomy with four retraction sutures and paired dentate-ligament release enabled dorsolateral access. Under magnification, the array was advanced ventrally via micro-forceps, retrieved contralaterally, circumferentially oriented using the midline electrode, tacked dorsally with Kwik-Sil, and a grounding wire was affixed to the paravertebral muscle.

### Electrophysiology recording and stimulation

i360 devices were interfaced via Omnetics/ZIF connectors to one (rats) or two (pigs) 32-channel Intan RHS2000 headstages, acquiring signals at 30 kHz. A paravertebral stainless-steel wire provided grounding, and impedances were checked pre-recording. Concentric microelectrode stimulation used the Intan third port with bipolar, charge-balanced, cathodic-first pulses (100 µA × 100 µs; 500 µA × 100 µs; 100 µA × 500 µs) and 60 s inter-stimulus intervals.

### Electrophysiological recordings from freely moving rats

We conducted recovery neural recordings in freely moving rats using the Intan RHS2000 system with a single 32-channel headstage. The electrode array was referenced and grounded via the stainless-steel wire in the paravertebral muscles.

For each session, we accessed the vascular port by removing the protective cap to expose the flat flexible cable (FFC) and grounding wire. The FFC was retrieved and connected to the headstage via an interfacing cable. Multiple daisy-chained extension cables provided sufficient length for unimpeded movement, allowing rats to navigate freely without mechanical constraints on the implanted device.

To prevent recording interruptions and data loss, we modified the Intan acquisition system to enable automatic file segmentation, saving data in one-minute intervals. These segmented files were subsequently concatenated offline into a single continuous dataset for analysis.

### Recovery recordings (rats)

Freely moving rats were connected via the vascular port’s FFC to a single 32-channel Intan RHS2000 headstage, with grounding via the paravertebral wire. Extension cables allowed unrestricted locomotion. Data were acquired continuously at 30 kHz with one-minute file segmentation and later concatenated offline.

### Kinematic assessment

Freely moving rats traversed a 1.5 m × 30 cm × 40 cm Plexiglas tunnel while being recorded at 60 fps (48 MP iPhone 15 Pro) with synchronized timestamps to 30 kHz electrophysiological data. The cable was secured with slack to prevent restriction of movement.

### Sensory testing (rodents)

Sensory testing was performed in recovered rats (*n* = 4) during naturally stationary periods in a confined chamber to minimize movement artifacts: calibrated von Frey filaments (60 g and 300 g at 1 Hz for 30 s epochs in ascending order) were applied to the plantar hindlimbs, followed by 183.3 Hz vibration (30 s per site) and nociceptive stimulation to the tail using a calibrated force applicator; all epochs were precisely time-stamped for alignment with electrophysiological recordings.

### Porcine testing

Porcine sensory testing was conducted under stable propofol anesthesia (following isoflurane transition confirmed by capnography), beginning with a 60-min baseline recording; stimuli were then applied in sequence to the right hindlimb plantar surface, right proximal hindlimb, left proximal hindlimb and left plantar surface using a hard-bristled brush at consistent pressure for 10 s per site with standardized inter-stimulus intervals, and the entire sequence was repeated twice to confirm reproducibility of evoked neural responses.

### Autonomic testing in the porcine model

Multimodal sensory stimuli were performed in the same order across all experiments at both lumbosacral and thoracic implantation. This was performed in the following order. The first 30 s were sans stimulus, followed by 30 s of left leg passive range of motion exercise, 30 additional seconds of rest, 30 s of left distal hind limb brushing, 30 s of proximal hind limb and gluteal brushing, 30 s of anal sphincter brushing, 30 s of right gluteal and proximal hind limb brushing, 30 s of distal hind limb brushing, 30 s of rest, 30 s of urethral sphincter balloon inflation, 30 s of rectal balloon inflation, followed by a final 30 s of rest. During this time, the device was actively recording first at the thoracic level and then repeated at the lumbosacral level.

### Electrode mapping

Electrode mapping was performed to preserve spatial localization relative to spinal anatomy. At implantation, device orientation was documented relative to anatomical landmarks, specifically noting which electrode pairs were positioned on the dorsal surface in relation to the central descending artery and dorsal midline. The device was stabilized with cyanoacrylate adhesive applied to the dura, preventing rotation around the spinal cord. The intrinsic adhesiveness of the parylene-C substrate to the spinal cord surface provided additional stabilization. Device position was verified at the terminal procedure to confirm no displacement had occurred relative to the documented anatomical landmarks.

Electrode channel identity was preserved throughout the recording chain using a standardized mapping protocol. Mapping of electrode locations from devices to recording unit channels was performed using a custom script that tracked electrode positions through the entire signal path: from the device electrode array through the FFC, the FFC to the zero-insertion-force (ZIF) connector on the intermediate connector PCB, the intermediate PCB to the recording unit connector, and finally to the recording unit channels. The script processed an array representing the original spatial positions of the electrodes on the device and mapped them to the corresponding recording unit channels, ensuring that the physical arrangement of electrodes was maintained during visualization and analysis.

### Data processing and analysis

Neural signals (30 kHz) were notch-filtered at 50 Hz (*Q* = 30), common-median referenced, and band-passed into seven bands (delta 1–4 Hz, theta 4–8 Hz, alpha 8–12 Hz, beta 12–30 Hz, low gamma 30–90 Hz, high gamma 90–200 Hz, high-frequency 200–3000 Hz) using bidirectional first-order Butterworth filters. Signal-to-noise ratio was quantified via Welch’s method (8192-point segments), a 75th-percentile frequency-threshold, and peak-to-RMS measures. Band-specific SNR was calculated as the ratio of mean power within each frequency band to mean power outside the band, with all SNR values expressed in decibels. Features (RMS and mean amplitude) were extracted from overlapping 500 ms windows for each channel and band. Kinematics (60 fps) were tracked in DeepLabCut (ResNet-50, 350 k iterations, >95% cross-validation) at seven landmarks, with low-confidence (< 0.7) points excluded and gaps ≤10 frames interpolated; hip position, joint positions, angles, and segment lengths were computed. Neural and kinematic streams were aligned by downsampling to 60 Hz (verified by cross-correlation) and z-scored. For sequence modeling, 9-step sliding windows predicted one-step-ahead biomechanics; data were split continuously 70/15/15% per epoch. For sensory and autonomic neural classification, time-stamped stimulation and baseline epochs were segmented into 100 ms windows and grouped into 300 ms sequences (history_length = 3) for supervised decoding. Datasets were partitioned 70/15/15% (train/validation/test) per epoch.

### Cross-correlation analysis

Signal velocity and directionality were analyzed similarly to previously described^20,53^. Electrode recorded signals were bandpass filtered (2nd order Butterworth filter, 200–3000 Hz) and notch filtered to remove mains noise. The cross-correlation coefficient was calculated between signals from proximal-distal electrode pairs on the spinal cord device using a rolling window approach, using a window width of 1 s and a step size of 50 ms. Each correlation coefficient slice was normalized by scaling its amplitude to match a standard deviation equal to 1. The time delays of the cross-correlation coefficient were converted to velocities using the known distance between proximal and distal electrodes (1.5 mm) and were plotted in heatmaps with an asinh-transformed y axis to more easily visualize slow velocities. Heatmap color scales were adjusted to transition from a gray to a heat color scheme above correlation values > 1 std to better visualize high correlation bands. The sum of correlation coefficient values between −100 and −0.5 m/s (*A*, afferent) and +0.5 to +100 m/s (*E*, efferent) were used to calculate the afferent–efferent dominance index per electrode pair, either per cross-correlation time slice or for all time slices. The afferent–efferent dominance index is defined as:

1 $$\frac{E-A}{{|E|}+{|A|}}$$

### Neural decoding models

Kinematic decoding employed a hierarchical bidirectional RNN with two BiLSTM layers (128, 64 units) followed by a GRU (96 units), each with recurrent dropout (0.05) and inter-layer batch normalization, three ELU-activated dense layers (96, 64, output) with L2 regularization (1 × 10^−^⁶), and was trained in TensorFlow on GPU using Adam (5 × 10^−^⁴) and a Huber loss. Sensory/autonomic classification used two BiLSTM layers (160, 96 units) and a BiGRU (128 units) with recurrent dropout (0.2), layer normalization, three ReLU-activated dense layers (128, 96, 64), and was trained with AdamW (weight decay 1 × 10^−^⁴) and a warm-up cosine schedule (max lr 4 × 10^−^⁴) with class weighting. Both models incorporated early stopping (patience 15–25 epochs), gradient clipping (clipnorm 0.5), and hyperparameter tuning on validation splits.

### Model evaluation and analysis

We benchmarked kinematic RNN decoding against Kalman and Wiener filters, Random Forest, XGBoost, and SVM (70 / 15 / 15% splits) using MAE, MSE, and *R*². Moreover, we used mutual information analysis as an external validation technique. Sensory classifiers were assessed on eight-condition, modality-only, and laterality frameworks via accuracy, precision, recall, F1, and ROC metrics. Temporal, frequency, and spatial contributions were quantified by sequential ablation and permutation importance, zeroing individual time segments, bands, or channels, and performance degradation was measured as absolute and percentage error change. SHAP analysis further quantified feature–output relationships by aggregating importance values by frequency band.

### Histological analysis

Tissue samples were fixed in 4% paraformaldehyde for 48 h, then transferred to 30% sucrose in phosphate-buffered saline for 72 h for cryo-protection. Samples were embedded in OCT (optimal cutting temperature compound), frozen, and sectioned at 10 μm thickness using a cryostat. Sections were stained with hematoxylin and eosin for histological assessment.

### Microneedle intraspinal stimulation

A custom polyimide frame (1 × 1.5 × 0.3 cm) with six guide holes was sutured dorsal to the i360 implant. Tungsten concentric microneedle electrodes (Science Products GmbH, Germany) were inserted through designated holes to a suture-limited depth and delivered four series of stimulation pulses at 60 s intervals. Following each session, the electrode was removed and the tract coagulated with monopolar diathermy for histological depth confirmation.

### Source localization

Neural signals (30 kHz) were preprocessed for artifact removal, notch-filtered at 60 Hz (*Q* = 30), common-median referenced, and band-passed into seven frequency bands (delta 1–4 Hz through high-frequency 200–3000 Hz) using zero-phase first-order Butterworth filters. RMS features were extracted from overlapping 200-ms windows per channel and band. Eight predefined stimulation sites along the porcine nerve trunk were represented by *x*-coordinates (longitudinal distance, increasing top-to-bottom) and *y*-coordinates (medio-lateral position; negative left, positive right). Feature matrices were z-score normalized and split 60%/40% (training/testing) with stratified class balance.

Stimulation-site localization employed a three-layer LSTM architecture (128 units each) with dropout (0.1), followed by a ReLU-activated dense layer (128 units) and linear output predicting continuous *x,y* coordinates. The model was trained in TensorFlow using Adam optimization (1 × 10^−^³) with MSE loss and early stopping (patience 20 epochs). Performance was assessed via MAE, with continuous predictions discretized to 1 mm × 1 mm regions for classification accuracy evaluation.

### Ethics

Every experiment involving animals was carried out following a protocol approved by an ethical commission. Surgical procedures were conducted in compliance with the UK Animals (Scientific Procedures) Act 1986 and approved by the Animal Welfare and Ethical Review Body at the University of Cambridge. Studies were performed under Project License No. PP6076787 (large animals) and PFF2068BC (rodents), with procedures conducted under Personal License No. I54111393.

### Reporting summary

Further information on research design is available in the Nature Portfolio Reporting Summary linked to this article.

## Supplementary information

Supplementary Information

Description of Additional Supplementary Files

Supplementary movie 1

Reporting Summary

Transparent Peer Review file

## Source data

Source Data

## Data Availability

The electrophysiological, kinematic, and processed machine learning data generated in this study have been deposited in the Zenodo database under accession code [10.5281/zenodo.20030734]. Any additional requests for information can be directed to, and will be fulfilled by, the corresponding author. Source data are provided with this paper.

## Code availability

The code used in this study has been deposited in the Zenodo database under accession code 10.5281/zenodo.20030734.

## References

[1] Jiang, L., Woodington, B., Carnicer-Lombarte, A., Malliaras, G. & Barone, D. G. Spinal cord bioelectronic interfaces: opportunities in neural recording and clinical challenges. *J. Neural Eng.* **19**, 021003 (2022). 10.1088/1741-2552/ac605f 35320780

[2] Capogrosso, M. et al. A brain-spine interface alleviating gait deficits after spinal cord injury in primates. *Nature* **539**, 284–288 (2016). 27830790 10.1038/nature20118 PMC5108412

[3] Fan, J. et al. A hyperflexible electrode array for long-term recording and decoding of intraspinal neuronal activity. *Adv. Sci.* **10**, 2303377 (2023). 10.1002/advs.202303377 PMC10667843 37870208

[4] Chalif, J. I. et al. Epidural spinal cord stimulation for spinal cord injury in humans: a systematic review. *J. Clin. Med.* **13**, 1090 (2024). 38398403 10.3390/jcm13041090 PMC10889415

[5] Moritz, C. et al. Non-invasive spinal cord electrical stimulation for arm and hand function in chronic tetraplegia: a safety and efficacy trial. *Nat. Med.* **30**, 1276–1283 (2024). 38769431 10.1038/s41591-024-02940-9 PMC11108781

[6] Herrity, A. N. et al. Targeting bladder function with network-specific epidural stimulation after chronic spinal cord injury. *Sci. Rep.* **12**, 11179 (2022). 35778466 10.1038/s41598-022-15315-2 PMC9249897

[7] Phillips, A. A. et al. An implantable system to restore hemodynamic stability after spinal cord injury. *Nat. Med.* **31**, 2946–2957 (2025). 40962906 10.1038/s41591-025-03614-w PMC12443590

[8] Squair, J. W. et al. Neuroprosthetic baroreflex controls haemodynamics after spinal cord injury. *Nature* **590**, 308–314 (2021). 33505019 10.1038/s41586-020-03180-w

[9] Angeli, C. A. et al. Recovery of over-ground walking after chronic motor complete spinal cord injury. *N. Engl. J. Med.* **379**, 1244–1250 (2018). 30247091 10.1056/NEJMoa1803588

[10] Gill, J. S., Asgerally, A. & Simopoulos, T. T. High-frequency spinal cord stimulation at 10 kHz for the treatment of complex regional pain syndrome: a case series of patients with or without previous spinal cord stimulator implantation. *Pain. Pr.* **19**, 289–294 (2019). 10.1111/papr.12739 30365222

[11] Wagner, F. B. et al. Targeted neurotechnology restores walking in humans with spinal cord injury. *Nature* **563**, 65–71 (2018). 30382197 10.1038/s41586-018-0649-2

[12] Minev, I. R. et al. Electronic dura mater for long-term multimodal neural interfaces. *Science* **347**, 159–163 (2015). 25574019 10.1126/science.1260318

[13] Hogan, M. K. et al. A wireless spinal stimulation system for ventral activation of the rat cervical spinal cord. *Sci. Rep.* **11**, 14900 (2021). 34290260 10.1038/s41598-021-94047-1 PMC8295294

[14] Bogduk, N. Functional anatomy of the spine. In *Handbook of Clinical Neurology* Vol. 136 (eds Masdeu, J. C. & González, R. G.) 675–688 (Elsevier, 2016). 10.1016/B978-0-444-53486-6.00032-6 27430435

[15] Woodington, B. J. et al. Flexible circumferential bioelectronics to enable 360-degree recording and stimulation of the spinal cord. *Sci. Adv.* **10**, eadl1230 (2024). 38718109 10.1126/sciadv.adl1230 PMC11078185

[16] Watson, C., Paxinos, G., Kayalioglu, G. & Heise, C. Atlas of the rat spinal cord. In The Spinal Cord (eds Watson, C., Paxinos, G. & Kayalioglu, G.) 238–306 (Elsevier, 2009).

[17] Bech, J. et al. The porcine corticospinal decussation: a combined neuronal tracing and tractography study. *Brain Res. Bull.* **142**, 253–262 (2018). 30086351 10.1016/j.brainresbull.2018.08.004

[18] Ahmed, R. U. et al. Porcine spinal cord injury model for translational research across multiple functional systems. *Exp. Neurol.* **359**, 114267 (2023). 36356636 10.1016/j.expneurol.2022.114267

[19] Guertin, P. A. & Steuer, I. Key central pattern generators of the spinal cord. *J. Neurosci. Res.* **87**, 2399–2405 (2009). 19326451 10.1002/jnr.22067

[20] Carnicer-Lombarte, A. et al. Ultraconformable cuff implants for long-term bidirectional interfacing of peripheral nerves at sub-nerve resolutions. *Nat. Commun.* **15**, 7523 (2024). 39214981 10.1038/s41467-024-51988-1 PMC11364531

[21] Nath, T. et al. Using DeepLabCut for 3D markerless pose estimation across species and behaviors. *Nat. Protoc.* **14**, 2152–2176 (2019). 31227823 10.1038/s41596-019-0176-0

[22] Carhart, M. R., He, J., Herman, R., D’Luzansky, S. & Willis, W. T. Epidural spinal-cord stimulation facilitates recovery of functional walking following incomplete spinal-cord injury. *IEEE Trans. Neural Syst. Rehabil. Eng.* **12**, 32–42 (2004). 15068185 10.1109/TNSRE.2003.822763

[23] Formento, E. et al. Electrical spinal cord stimulation must preserve proprioception to enable locomotion in humans with spinal cord injury. *Nat. Neurosci.* **21**, 1728–1741 (2018). 30382196 10.1038/s41593-018-0262-6 PMC6268129

[24] Capogrosso, M. et al. Configuration of electrical spinal cord stimulation through real-time processing of gait kinematics. *Nat. Protoc.* **13**, 2031–2061 (2018). 30190556 10.1038/s41596-018-0030-9

[25] Kanakis, A. K. et al. Electrical stimulation and motor function rehabilitation in spinal cord injury: a systematic review. *Cureus* **16**, e61436 (2024). 38947571 10.7759/cureus.61436 PMC11214755

[26] Lin, A. et al. A review of functional restoration from spinal cord stimulation in patients with spinal cord injury. *Neurospine* **19**, 703–734 (2022). 36203296 10.14245/ns.2244652.326 PMC9537842

[27] Lucci, V. E. M. Recent updates in autonomic research: advances in the understanding of autonomic dysfunction after spinal cord injury. *Clin. Auton. Res.* **33**, 83–85 (2023). 37071264 10.1007/s10286-023-00944-y

[28] Henke, A. M., Billington, Z. J. & Gater, D. R. Autonomic dysfunction and management after spinal cord injury: a narrative review. *J. Pers. Med.* **12**, 1110 (2022). 35887607 10.3390/jpm12071110 PMC9320320

[29] Karlsson, A.-K. Overview: autonomic dysfunction in spinal cord injury: clinical presentation of symptoms and signs. In *Progress in Brain Research* Vol. 152 (eds Weaver, L. C. & Polosa, C.) 1–8 (Elsevier, 2006). 10.1016/S0079-6123(05)52034-X 16198689

[30] Dimitrijevic, M. R., Gerasimenko, Y. & Pinter, M. M. Evidence for a spinal central pattern generator in humans. *Ann. N. Y. Acad. Sci.* **860**, 360–376 (1998). 9928325 10.1111/j.1749-6632.1998.tb09062.x

[31] Moraud, E. M. et al. Mechanisms underlying the neuromodulation of spinal circuits for correcting gait and balance deficits after spinal cord injury. *Neuron* **89**, 814–828 (2016). 26853304 10.1016/j.neuron.2016.01.009

[32] Hazelgrove, B. et al. Detection of spinal action potentials with subdural electrodes in freely moving rodents. *Sci. Rep.* **15**, 30635 (2025). 40835679 10.1038/s41598-025-15795-y PMC12368248

[33] Wu, Y. et al. Ultraflexible electrodes for recording neural activity in the mouse spinal cord during motor behavior. *Cell Rep.* **43**, 114199 (2024). 38728138 10.1016/j.celrep.2024.114199 PMC11233142

[34] Russman, S. M. et al. The conformal, high-density SpineWrap microelectrode array for focal stimulation and selective muscle recruitment. *Adv. Funct. Mater.* **35**, 2420488 (2025). 40787278 10.1002/adfm.202420488 PMC12330848

[35] Huang, S. et al. Anisotropic hydrogel microelectrodes for intraspinal neural recordings in vivo. *Nat. Commun.* **16**, 1127 (2025). 39875371 10.1038/s41467-025-56450-4 PMC11775234

[36] Sandean, D. Management of acute spinal cord injury: a summary of the evidence pertaining to the acute management, operative and non-operative management. *World J. Orthop.* **11**, 573–583 (2020). 33362993 10.5312/wjo.v11.i12.573 PMC7745491

[37] Grillner, S. & El Manira, A. Current principles of motor control, with special reference to vertebrate locomotion. *Physiol. Rev.* **100**, 271–320 (2020). 31512990 10.1152/physrev.00015.2019

[38] Courtine, G. & Sofroniew, M. V. Spinal cord repair: advances in biology and technology. *Nat. Med.* **25**, 898–908 (2019). 31160817 10.1038/s41591-019-0475-6

[39] Coote, J. H. & Spyer, K. M. Central control of autonomic function. *Brain Neurosci. Adv.* **2**, 2398212818812012 (2018). 32166159 10.1177/2398212818812012 PMC7058216

[40] Violante, I. R. et al. Non-invasive temporal interference electrical stimulation of the human hippocampus. *Nat. Neurosci.* **26**, 1994–2004 (2023). 37857775 10.1038/s41593-023-01456-8 PMC10620081

[41] Mirzakhalili, E., Barra, B., Capogrosso, M. & Lempka, S. F. Biophysics of temporal interference stimulation. *Cell Syst.* **11**, 557–572.e5 (2020). 33157010 10.1016/j.cels.2020.10.004

[42] Sunshine, M. D. et al. Restoration of breathing after opioid overdose and spinal cord injury using temporal interference stimulation. *Commun. Biol.* **4**, 107 (2021). 33495588 10.1038/s42003-020-01604-x PMC7835220

[43] Carnicer-Lombarte, A., Chen, S. T., Malliaras, G. G. & Barone, D. G. Foreign body reaction to implanted biomaterials and its impact in nerve neuroprosthetics. *Front. Bioeng. Biotechnol.* **9**, 622524 (2021). 33937212 10.3389/fbioe.2021.622524 PMC8081831

[44] Oldroyd, P. & Malliaras, G. G. Achieving long-term stability of thin-film electrodes for neurostimulation. *Acta Biomater.* **139**, 65–81 (2022). 34020055 10.1016/j.actbio.2021.05.004

[45] Oldroyd, P., Gurke, J. & Malliaras, G. G. Stability of thin film neuromodulation electrodes under accelerated aging conditions. *Adv. Funct. Mater.* **33**, 2208881 (2023).

[46] Davis, O. C. & Price, T. J. Tiam1 creates a painful link between dendritic spine remodeling and NMDA receptors. *Neuron* **111**, 1993–1995 (2023). 37413965 10.1016/j.neuron.2023.06.001

[47] Hosseini, S. M. et al. Circuit reconstruction after traumatic spinal cord injury by cellular and pharmacological approaches. *J. Neurosci.* **45**, e0362252025 (2025). 40744729 10.1523/JNEUROSCI.0362-25.2025 PMC12392074

[48] Saadoun, S. et al. Duroplasty for injured cervical spinal cord with uncontrolled swelling: protocol of the DISCUS randomized controlled trial. *Trials* **24**, 497 (2023). 37550727 10.1186/s13063-023-07454-2 PMC10405486

[49] Kathe, C. et al. Wireless closed-loop optogenetics across the entire dorsoventral spinal cord in mice. *Nat. Biotechnol.* **40**, 198–208 (2022). 34580478 10.1038/s41587-021-01019-x PMC7612390

[50] Zhu, B., Shin, U. & Shoaran, M. Closed-loop neural prostheses with on-chip intelligence: a review and a low-latency machine learning model for brain state detection. *IEEE Trans. Biomed. Circuits Syst.* **15**, 877–897 (2021). 34529573 10.1109/TBCAS.2021.3112756 PMC8733782

[51] Famm, K., Litt, B., Tracey, K. J., Boyden, E. S. & Slaoui, M. A jump-start for electroceuticals. *Nature* **496**, 159–161 (2013). 23579662 10.1038/496159a PMC4179459

[52] Wellman, S. M. et al. A materials roadmap to functional neural interface design. *Adv. Funct. Mater.* **28**, 1701269 (2018). 29805350 10.1002/adfm.201701269 PMC5963731

[53] Klus, J. et al. Development of novel signal and spike velocity analysis tools in compact peripheral nerve recording designs. *J. Neural Eng.* **22**, ae0c3b (2025). 10.1088/1741-2552/ae0c3b 41005323

