# Supplementary material for: Surface circumferential spinal cord recording in freely moving rodents
Source: Nat Commun. 2026 Jul 13;17:8610. doi: 10.1038/s41467-026-75128-z (PMC13487197; doi:10.1038/s41467-026-75128-z)

Supplementary Figure 1 | Surgical implantation steps in rodents. Sequential intraoperative photographs illustrating the circumferential device implantation procedure. Top row, left to right: (1) exposure of posterior spinal elements following midline incision and muscle dissection; (2) laminectomy exposing the spinal cord; (3) passage of 7-0 polypropylene guiding suture beneath the spinal cord through the ventral epidural plane; (4) retrieval of the suture after threading through the device eyelet. Bottom row, left to right: (5&6) circumferential wrapping and stabilization of the electrode array around the spinal cord; (7) suturing of paravertebral and dorsal musculature to secure the device in position; (8) externalization of the flexible flat cable (FFC) through a subcutaneous vascular port with final postoperative view showing the externalized port and closed skin.

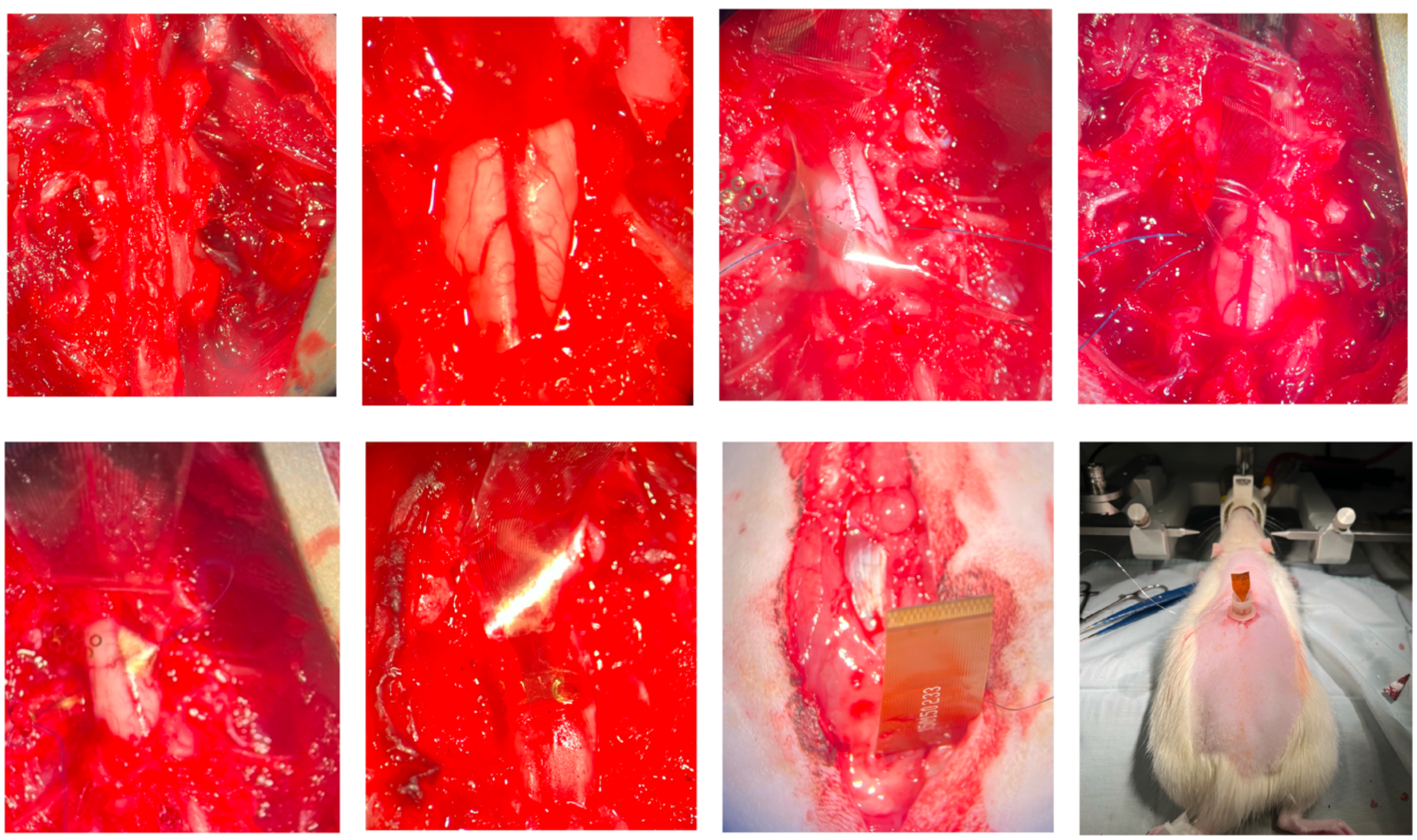

Supplementary Figure 2 | Schematic of the multilayer electrode array fabrication for the circumferential spinal cord device

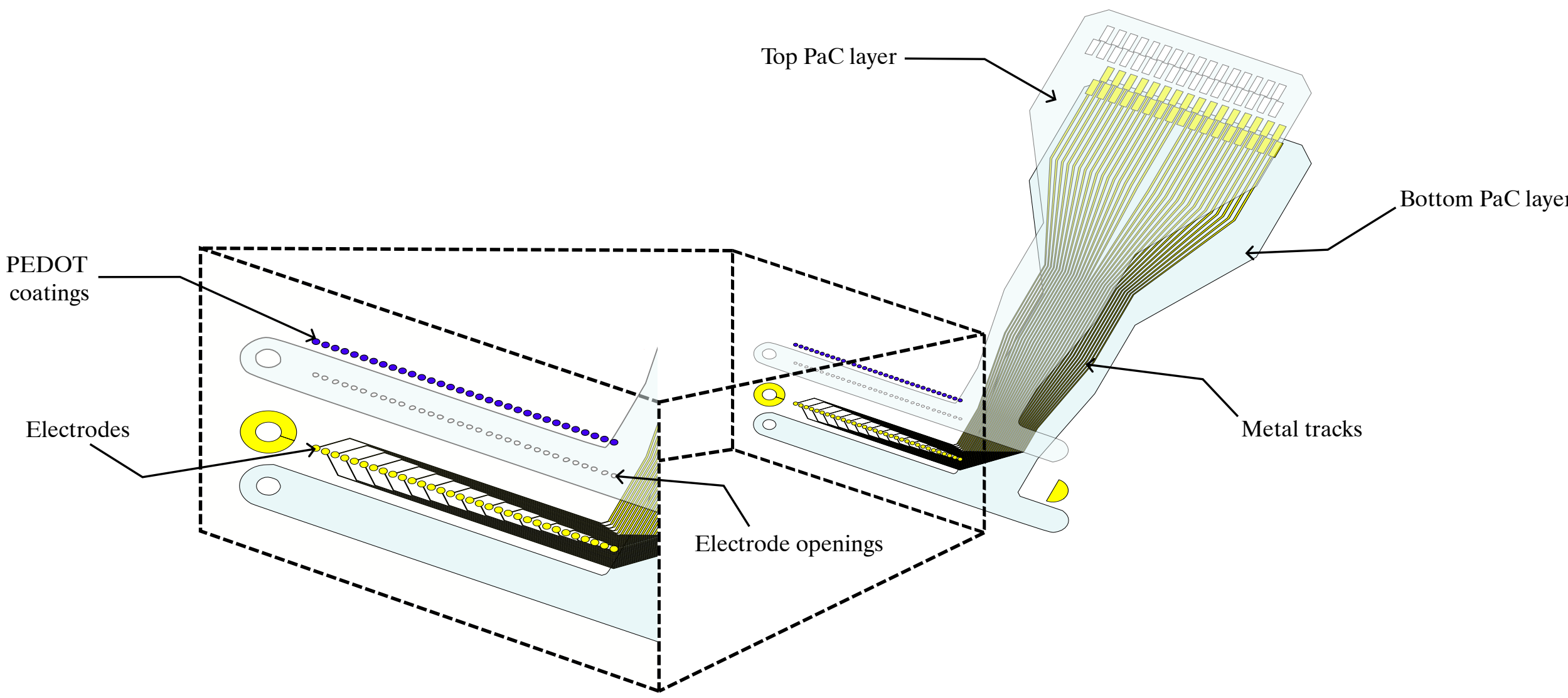

Supplementary Figure 3 | Postoperative electrode impedance measurements in porcine models. Box plot shows impedance values (kΩ) measured post-implantation across all electrodes from n = 3 porcine implants (n = 64 channels per animal, n = 192 electrodes total). Individual data points are shown as overlaid dots. Box plot shows median (centre line), 25th–75th percentile (box bounds), 1.5× IQR (whiskers), and individual outliers (points).

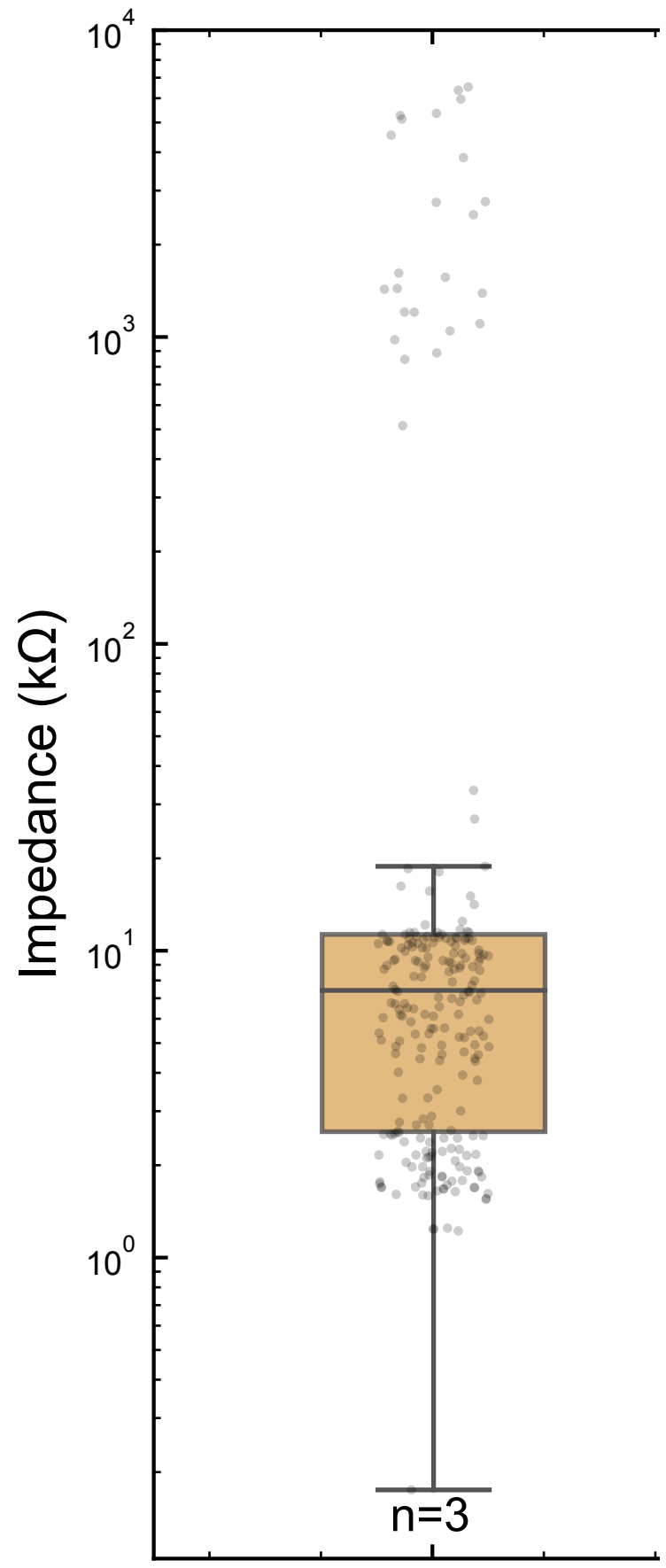

Supplementary Figure 4 | Electrode-by-electrode impedance stability. Paired impedance measurements at 1 kHz for all 15 implanted rodent devices (32 channels per animal, n = 480 electrodes total, 15 biological replicates). Each grey line connects the pre- and post-implantation impedance values of a single electrode on a log scale (kΩ). The overlapping appearance reflects the full dataset plotted simultaneously to maintain transparency across all 480 electrode pairs. The population-level analysis shows no significant change across implantation which is supported by a two-sided paired t-test ($t(14) = 0.86$, $p = 0.412$, Cohen's $d = 0.29$).

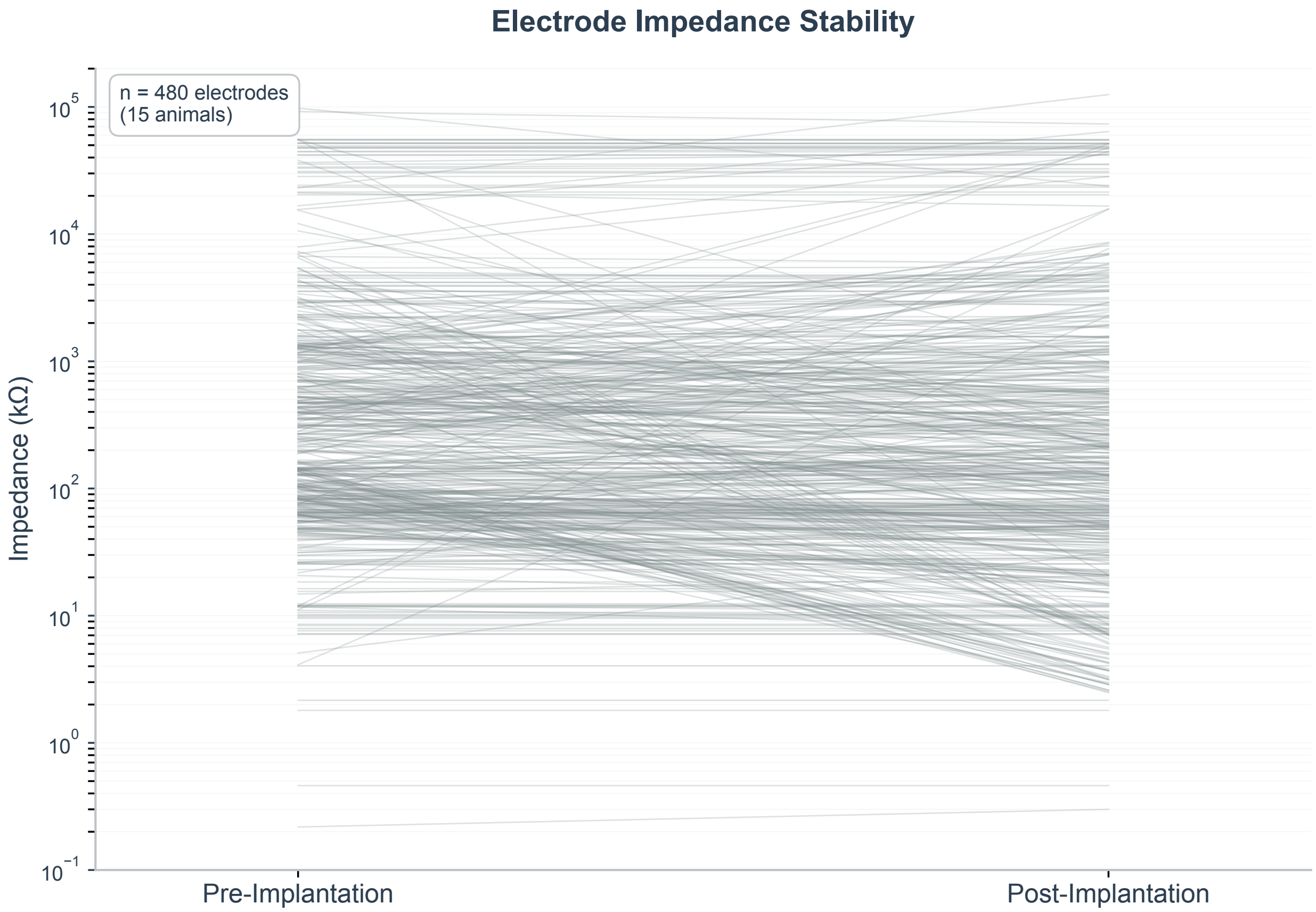

Supplementary Figure 5 | LSTM-GRU architecture for kinematic decoding. Left: representative input feature matrix (frame 100 of 120) showing RMS and mean amplitude values across 32 spinal channels (x-axis) and 7 frequency bands (y-axis: delta, theta, alpha, beta, low gamma, high gamma, high-frequency). Color intensity represents feature magnitude (purple = low, yellow = high). Vertical cyan lines delineate individual channels. Right: hierarchical deep learning network architecture. Input layer receives the 32-channel × 7-band feature matrix. Two bidirectional LSTM layers (Bi-LSTM 1: 128 units; Bi-LSTM 2: 64 units) perform temporal modelling, each followed by batch normalization. A GRU layer (96 units) with layer normalization integrates contextual information. Two fully connected dense layers (Dense 1: 96 units; Dense 2: 64 units) perform feature regression. Output layer predicts 22 biomechanical features.

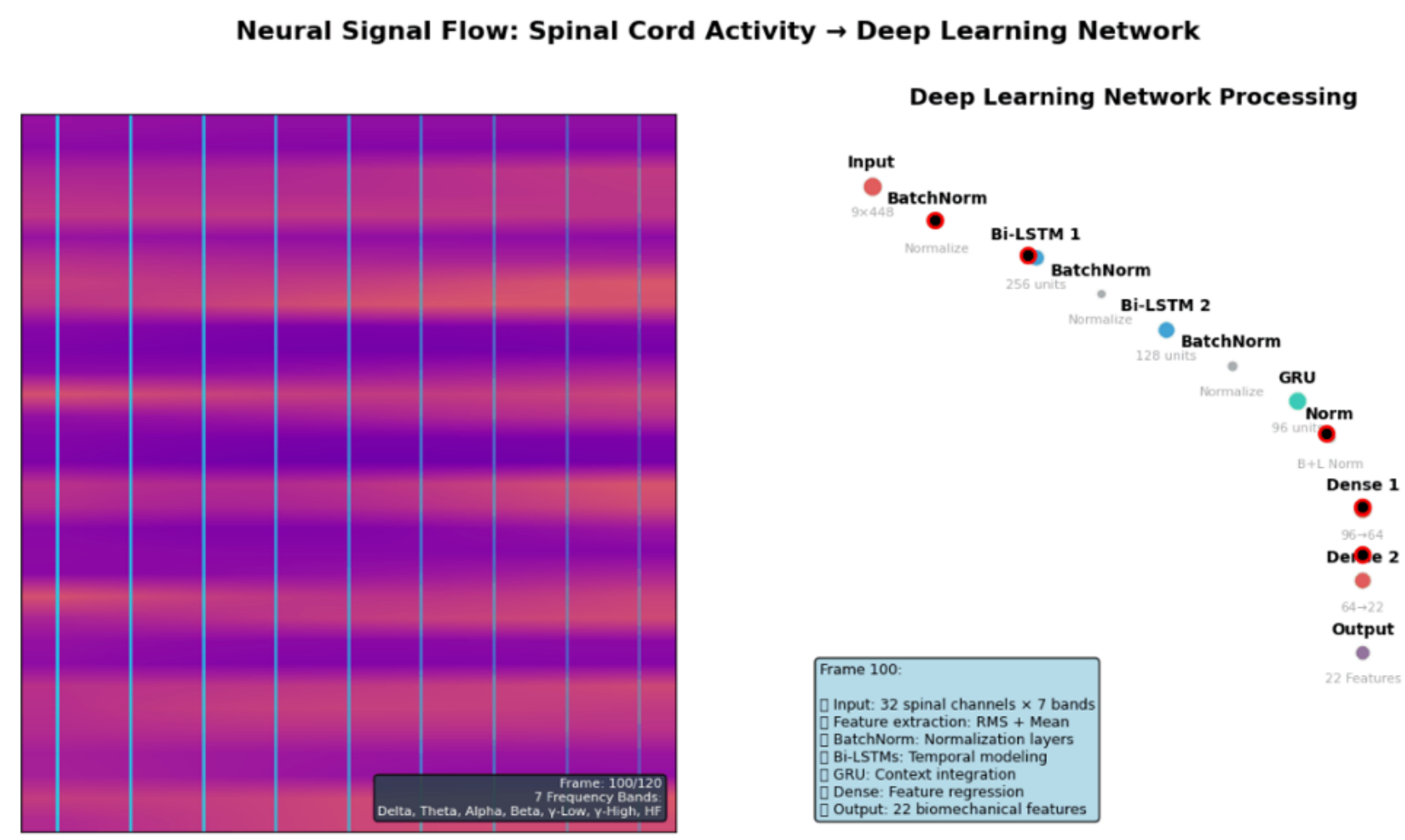

Supplementary Figure 6 | Training convergence curves for kinematic decoding using the LSTM-GRU model. Training (blue) and validation (orange) curves across 150 epochs for three metrics: left, Huber loss; center, mean absolute error (MAE); right, mean squared error (MSE). Data represent a single training run on pooled data from n = 5 animals (70% training, 15% validation, 15% test split).

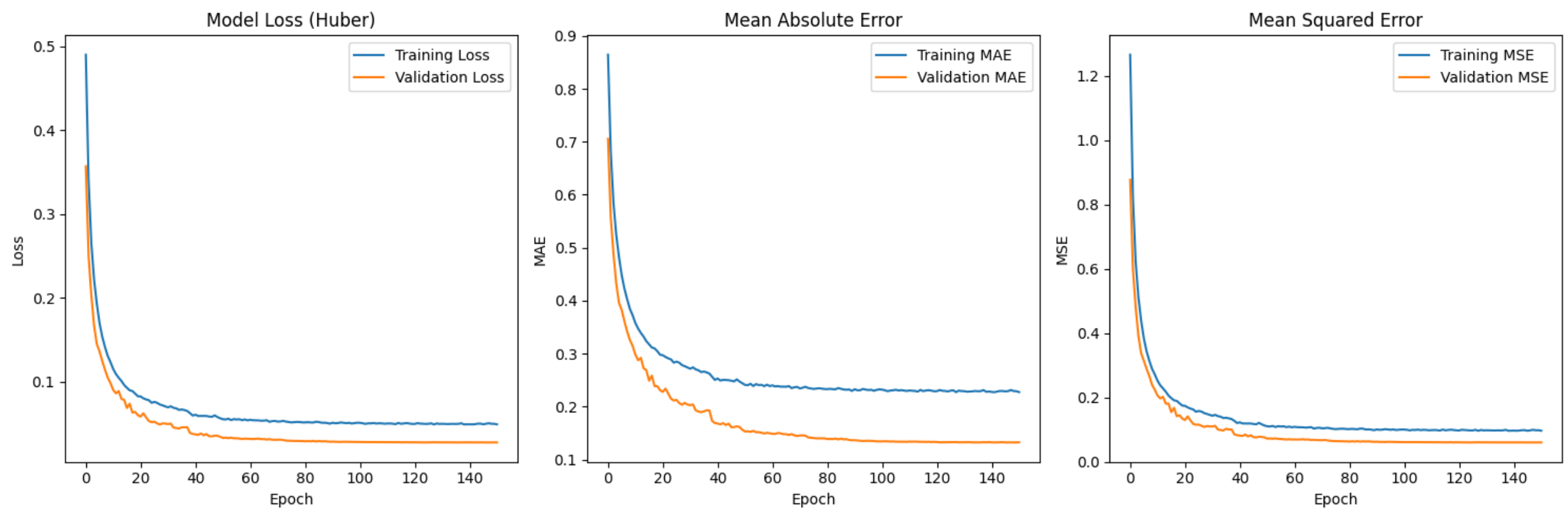

Supplementary Figure 7 | Quantitative decoding performance metrics for locomotor kinematics in rodents. Table of mean absolute error (MAE), root mean squared error (RMSE), and coefficient of determination (R^2) for all 22 biomechanical features decoded by the LSTM-GRU model on held-out test data (n = 5 animals, test set n = 3,441 time samples). Features include tracked landmark positions (ankle, hip, knee, toe, tail segments), joint angles (hip–knee–ankle, knee–ankle–toe, tail), and segment lengths. MAE and RMSE are reported in normalized units following z-score standardization of kinematic outputs during preprocessing.

Motor_rodent - Performance Metrics
LSTM-GRU Model Evaluation

Feature	MAE	RMSE	R²
Ankle R_rel_x	0.0889	0.1299	0.9824
Ankle R_rel_y	0.1165	0.1821	0.9675
Hip X	0.1351	0.2079	0.9561
Hip Y	0.1285	0.214	0.9556
Knee R_rel_x	0.1324	0.221	0.9515
Knee R_rel_y	0.1494	0.2385	0.9415
Tail 1 R_rel_x	0.1158	0.2018	0.9597
Tail 1 R_rel_y	0.1283	0.1982	0.9608
Tail 2 R_rel_x	0.1345	0.2063	0.9579
Tail 2 R_rel_y	0.1257	0.203	0.9591
Tail 3 R_rel_x	0.1252	0.1879	0.9617
Tail 3 R_rel_y	0.1175	0.1879	0.9641
Toe R_rel_x	0.1083	0.1621	0.9714
Toe R_rel_y	0.1125	0.1686	0.9698
angle_Hip R_Knee R_Ankle R	0.1453	0.2571	0.9334
angle_Knee R_Ankle R_Toe R	0.1553	0.2657	0.9283
angle_Tail 1 R_Tail 2 R_Tail 3 R	0.162	0.3134	0.9056
length_Ankle R_Toe R	0.1378	0.2809	0.9185
length_Hip R_Knee R	0.1583	0.3209	0.8978
length_Knee R_Ankle R	0.1098	0.1874	0.9628
length_Tail 1 R_Tail 2 R	0.1363	0.2151	0.9507
length_Tail 2 R_Tail 3 R	0.1198	0.1964	0.9579

Supplementary Figure 8 | Scatter plot matrix of predicted versus actual biomechanical feature values for locomotor decoding. Each panel shows predicted (y-axis) versus true (x-axis) values for one of the 22 biomechanical features decoded by the LSTM-GRU model on held-out test data (n = 5 animals, n = 3,441-time samples). R^2 values are indicated in each panel title. Red dashed line indicates perfect prediction (slope = 1, intercept = 0). Point density is represented by color intensity (purple = low density, dark = high density).

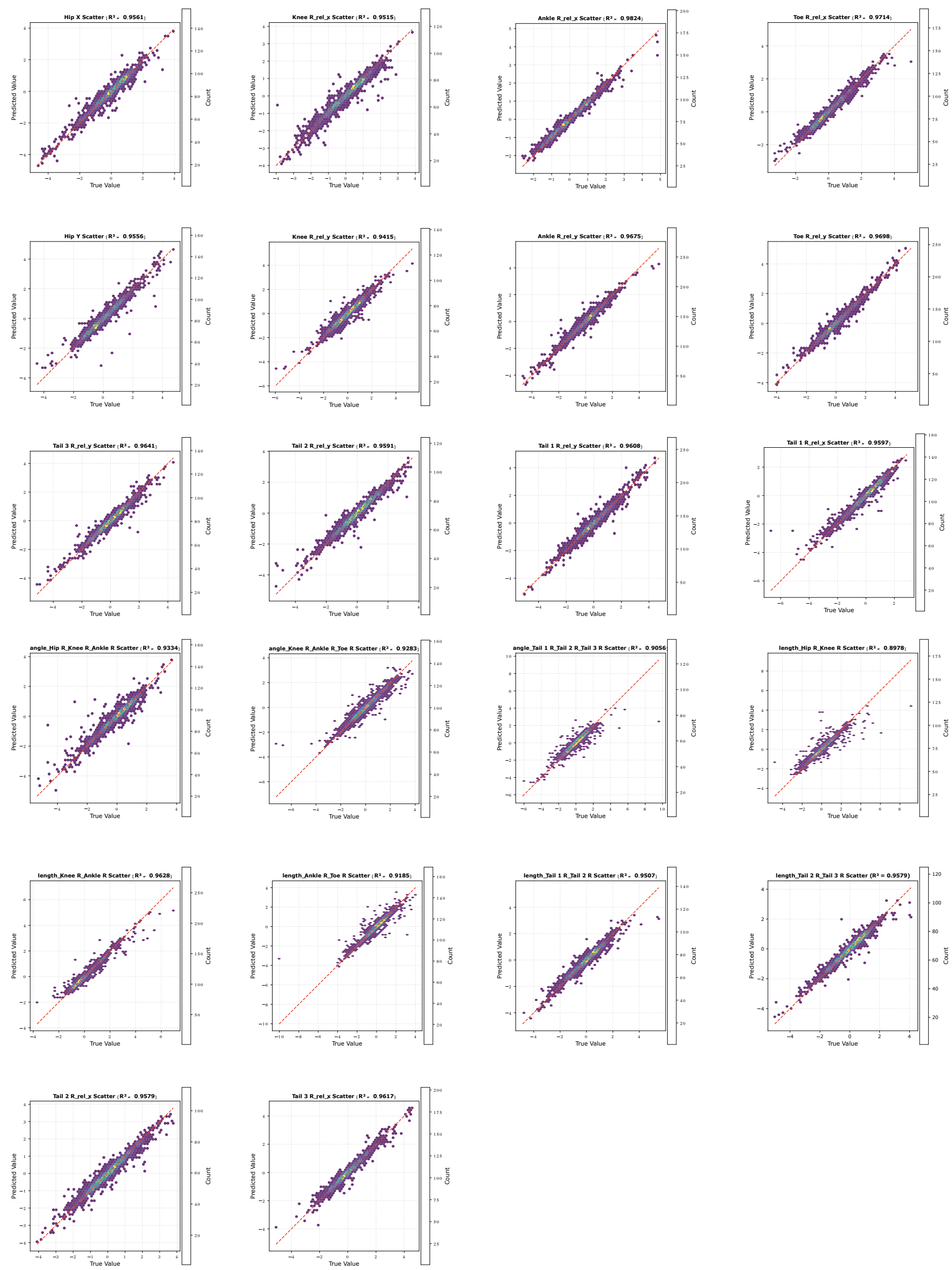

Supplementary Figure 9 | Time-series comparison of actual and predicted joint trajectories during locomotion. Three-dimensional time-series plot showing actual (solid, dark colors) and predicted (lighter shades) trajectories for four key joints : hip (blue/light blue), knee (green/light green), ankle (red/pink), and toe (purple/light purple) , across 75 consecutive time steps from a representative locomotion epoch. Y-axis represents normalized position values (z-scored). Data shown are from a single representative recording session from one animal; equivalent performance across all 5 animals and all 22 features is documented in Supplementary Figs. 7–8.

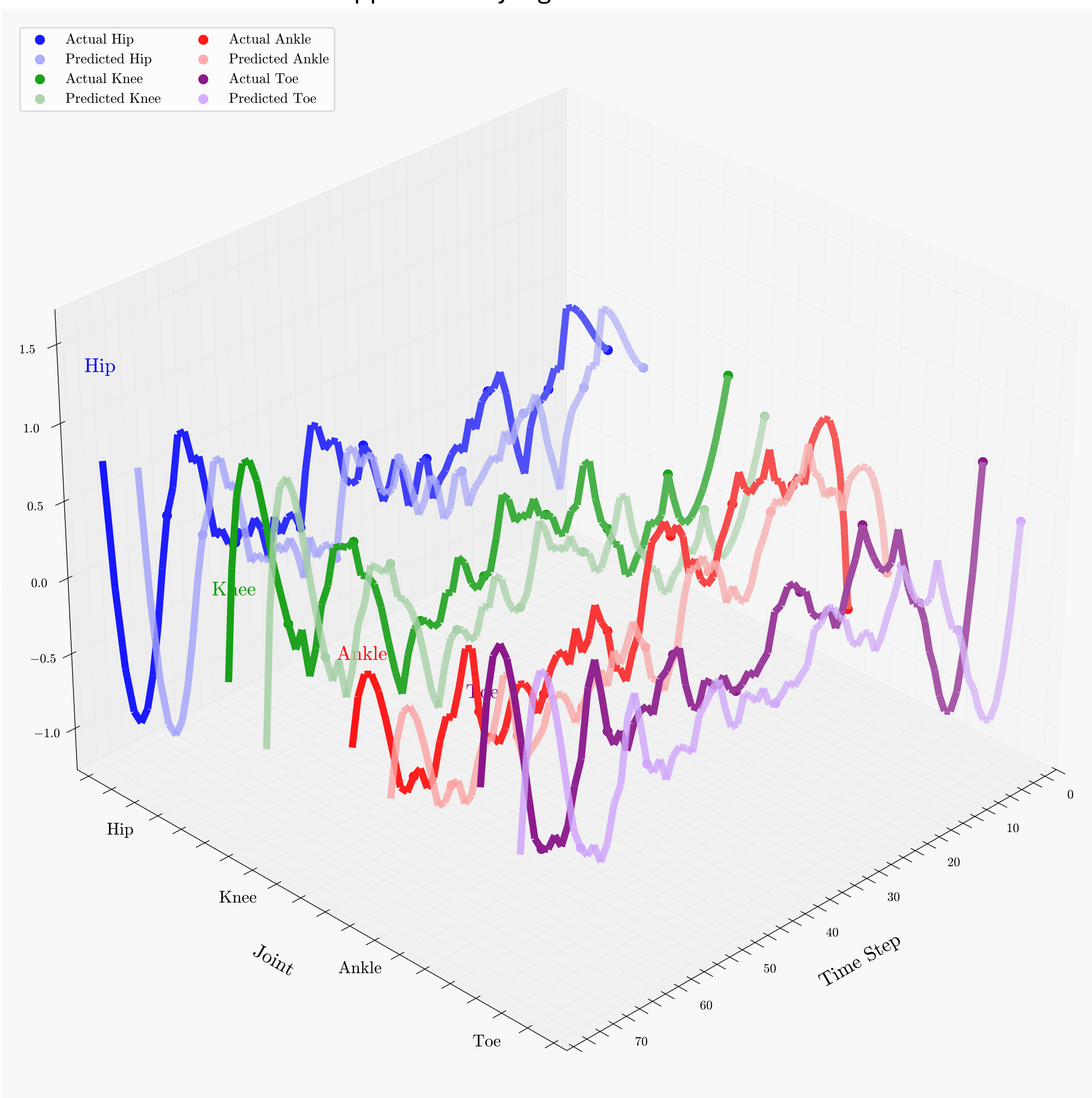

Figure 10 | Frequency-band ablation impact on sensory classification accuracy. Bar plot showing accuracy drop when each frequency band is systematically removed from the input to the LSTM-GRU classifier (n = 5 animals, pooled test set). Each bar represents the absolute decrease in classification accuracy relative to the full-band model when that band is withheld. Bars are ordered by decreasing impact. Colors indicate frequency bands. Red dashed line indicates the 0.05 accuracy drop threshold, above which band removal is considered to have a meaningful impact on classification performance.

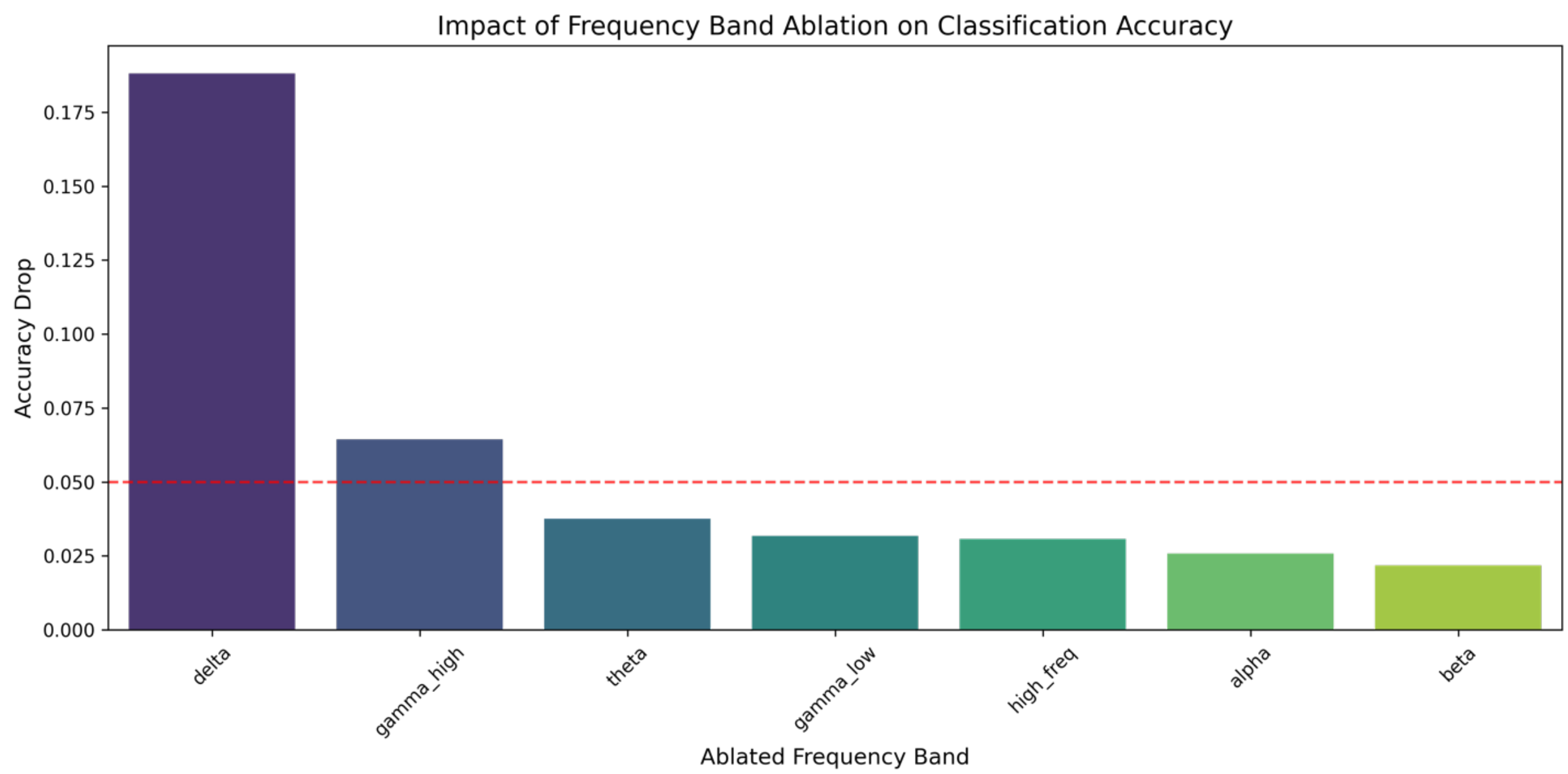

Supplementary Figure 11 | Frequency-band importance distribution from SHAP analysis. Box plots showing SHAP importance score distributions across seven frequency bands for the kinematic decoding model (n = 5 animals, pooled test set). Each data point represents the SHAP importance score for one channel–timepoint combination. Box plots show median (center line), 25th–75th percentile (box bounds), 1.5× IQR (whiskers), and individual outliers (open circles). Colors indicate frequency bands.

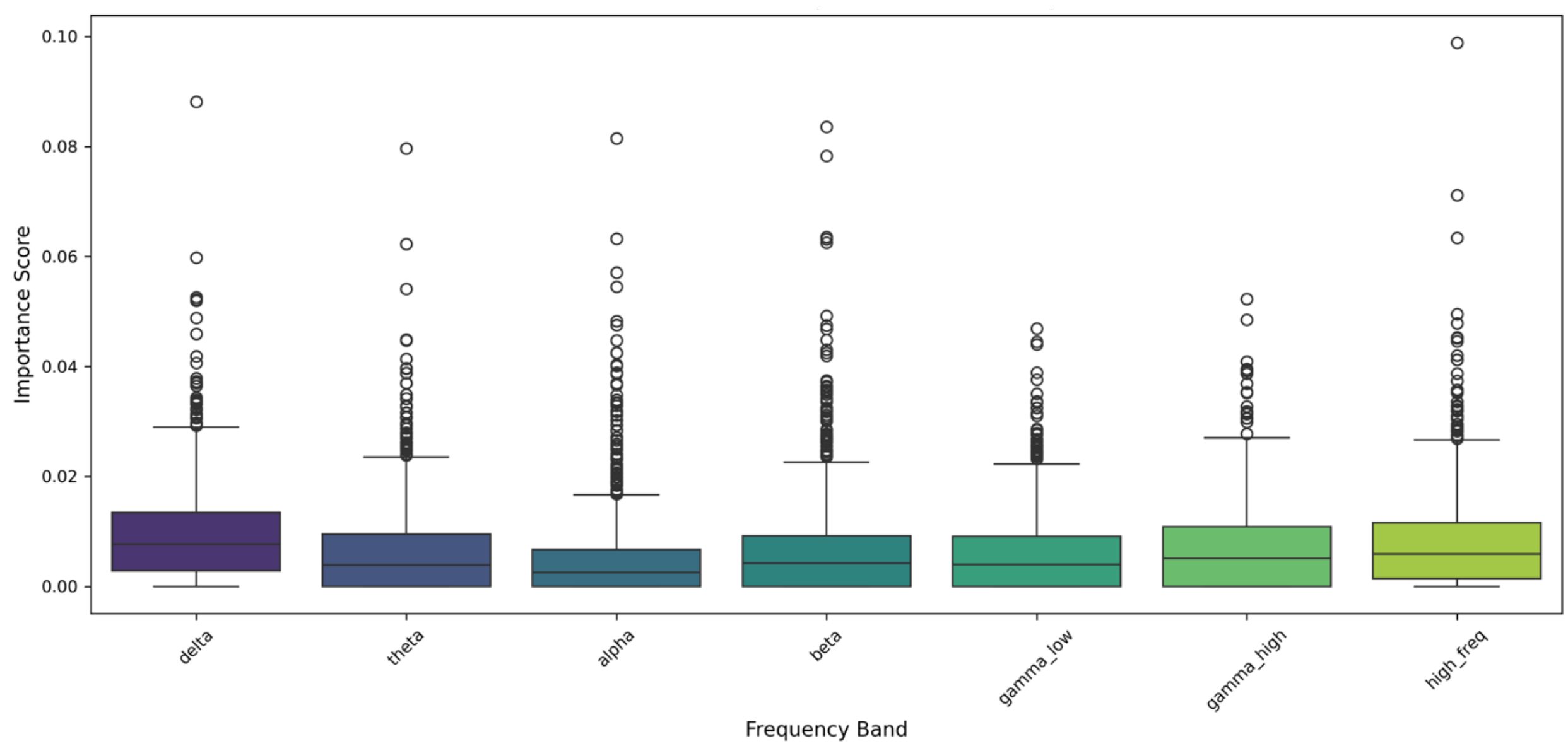

Supplementary Figure 12 | Temporal window importance analysis for decoding performance across recent time steps. Bar plot showing normalized importance scores for each of the 9 input time steps (x-axis, 1 = oldest, 9 = most recent) derived from two independent methods: ablation (blue) and permutation importance (orange). Each bar represents the normalized contribution of that time step to decoding performance when removed (ablation) or randomly shuffled (permutation). n = 5 animals, pooled test set.

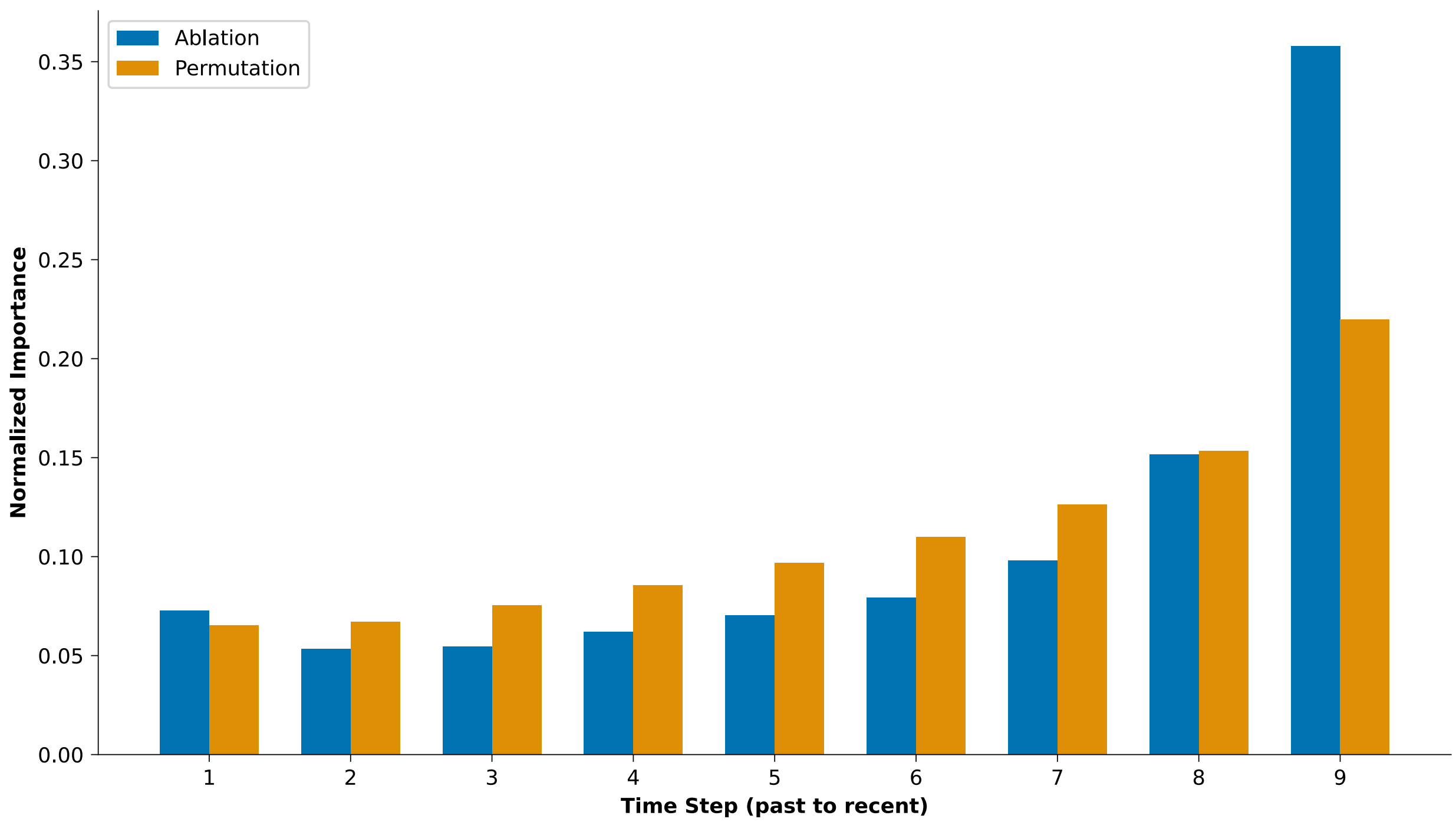

Supplementary Figure 13 | Workflow table of the neural decoding pipeline for locomotor kinematics with external validation. Table summarizing the 11-step decoding pipeline applied to circumferential spinal recordings. Steps 1–4 (blue) cover preprocessing and feature extraction: LFP notch filtering and common median referencing, RMS and mean feature extraction across 7 frequency bands and 32 channels (448 features total), DeepLabCut-based biomechanical output extraction, and dataset preparation including alignment, resampling and normalization. Step 5 (green) shows the primary LSTM-GRU neural network (history = 9 timesteps), which achieved the best performance (MAE = 0.1166). Steps 6–9 (orange) show benchmark comparators: Kalman filter (MAE = 0.753), Wiener filter (MAE = 0.566), Random Forest (MAE = 0.244), and XGBoost (MAE = 0.349). Steps 10–11 provide independent validation via feature importance analysis (Random Forest and XGBoost both identifying delta and theta bands as dominant) and mutual information analysis confirming frequency-independent validation of low-frequency band superiority. All models were evaluated on the same held-out test set (n = 5 animals, n = 3,441-time samples, 70/15/15% train/validation/test split)

Step	Method/Technique	Description	Output	Result/Performance
1	LFP Preprocessing	Notch filters (50/60Hz), Common Median Referencing (CMR), Bandpass filters	Cleaned LFP signal by band (delta to high_freq)	Input to feature extraction
2	Feature Extraction	RMS and Mean in sliding window	448 LFP features: 7 bands × 2 stats × 32 ch	Used in all models
3	Biomechanical Output Extraction	DeepLabCut tracking → joint positions, angles, segment lengths	Relative, angular, and absolute joint features	Used as targets for model training
4	Dataset Preparation	LFP and DLC alignment + resampling, normalization	Matched samples across modalities	21 biomechanical features (Y)
**5**	**LSTM-GRU Neural Network**	**Deep model with temporal memory (history = 9 timesteps)**	**Trained end-to-end regression**	**MAE = 0.1166**, best performance
6	Kalman Filter	Recursive linear estimator, 1 per output feature	Smoothed, framewise prediction	MAE = 0.7530, MSE = 0.9383
7	Wiener Filter	Linear regression per feature (Ridge)	Baseline linear decoder	MAE = 0.5656, MSE = 0.6422
8	Random Forest	Tree-based ensemble, nonlinear with feature importance	Model benchmark + interpretability	MAE = 0.2436, MSE = 0.1539
9	XGBoost	Boosted trees, tuned for compact regression	Alternative nonlinear model	MAE = 0.3489, MSE = 0.2488
10	Feature Importance Analysis	Mean importance per band, stat, and channel	Band-level and channel-level insights	RF: delta > theta; XGB: alpha > theta
11	Mutual Information Analysis	MI between each LFP feature and each biomech feature	Frequency-independent validation	alpha, theta, delta bands superior

Supplementary Figure 14 | Training convergence curves for sensory stimulus classification in rodents using LSTM-GRU. Training (blue) and validation (orange) curves across 120 epochs for two metrics: left, cross-entropy loss; right, classification accuracy. Data represent a single training run on pooled data from n = 4 animals (70% training, 15% validation, 15% test split).

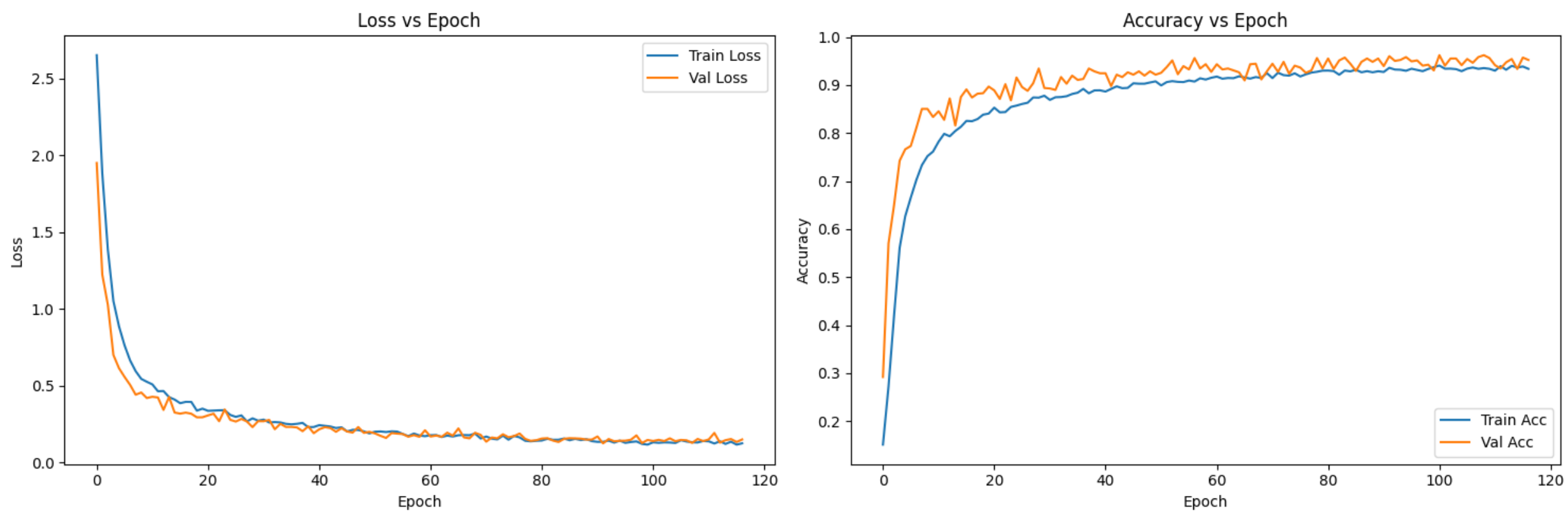

Supplementary Figure 15 | Radar plot of class-wise F1 scores for multimodal sensory stimulus classification in freely moving rodents. Radar plot showing F1 scores for each of the eight stimulus classes decoded by the LSTM-GRU classifier (n = 4 animals, total test samples n = 1,010). Radial axis ranges from 0 to 1.0, with concentric rings at 0.2 intervals. Classes shown clockwise: pressure left 60 g, pressure left 300 g, no stimulation, tail pain, vibration right, pressure right 60 g, pressure right 300 g, vibration left. The shaded blue polygon represents the F1 score profile across all classes. Full per-class metrics are provided in Supplementary Fig. 16

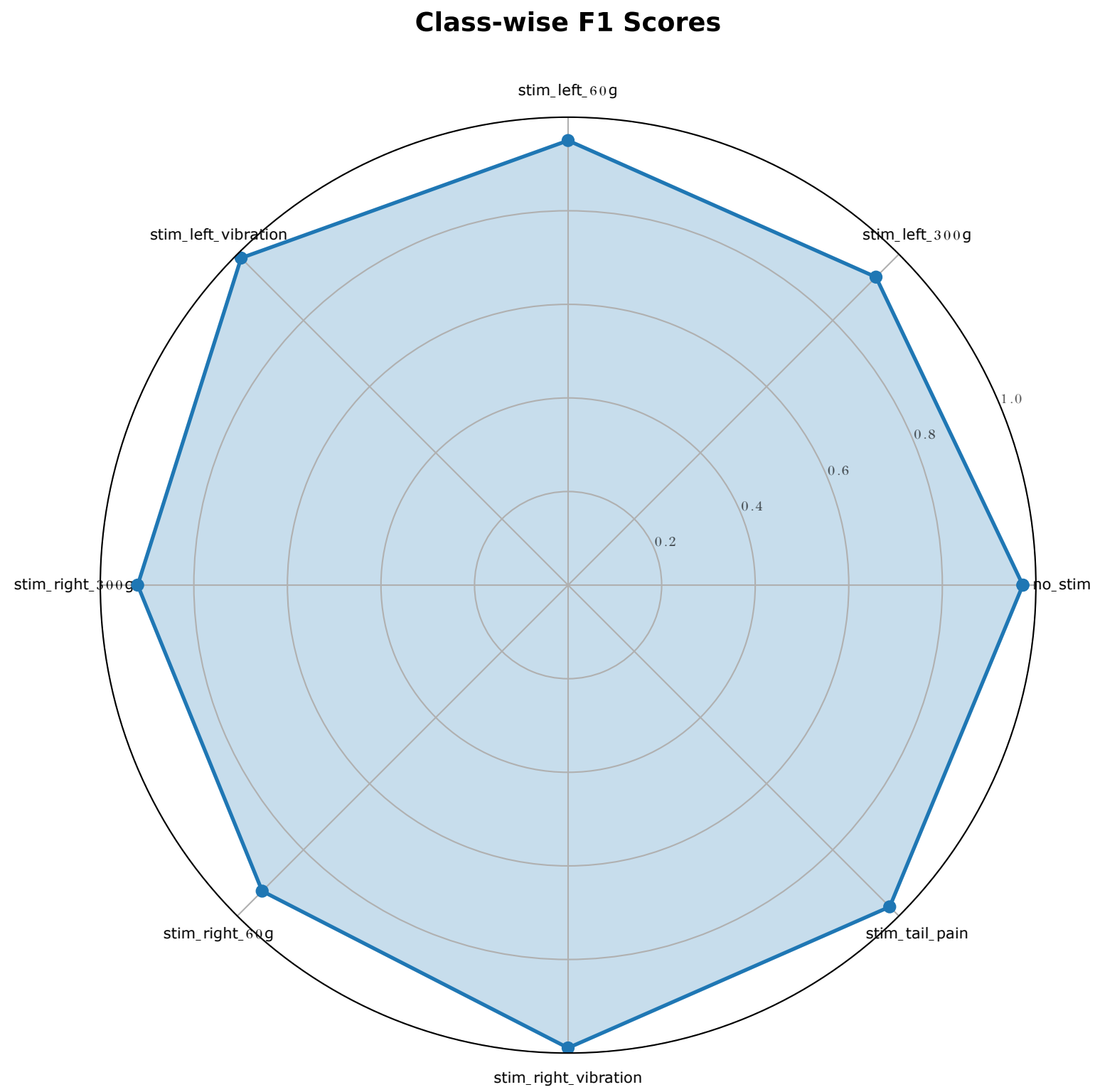

Supplementary Figure 16 | Performance metrics for multimodal sensory stimulus detection in rodents. Table of per-class precision, recall/sensitivity, F1 score, specificity, and ROC AUC for the eight-class sensory stimulus classifier evaluated on held-out test data (n = 4 animals, total test samples n = 1,010). Support column indicates the number of test samples per class. All metrics were computed on temporally held-out test data from the pooled dataset.

Feature	Precision	Recall/Sensitivity	F1 Score	Specificity	ROC AUC	Support
no_stim	0.976	0.9683	0.9721	0.9966	0.9983	126
stim_left_300g	0.9409	0.9211	0.9309	0.9866	0.9964	190
stim_left_60g	0.9709	0.9302	0.9501	0.9925	0.9966	215
stim_left_vibration	1.0	0.9773	0.9885	1.0	1.0	44
stim_right_300g	0.9076	0.933	0.9201	0.9795	0.9954	179
stim_right_60g	0.9071	0.9432	0.9248	0.9796	0.995	176
stim_right_vibration	0.9783	1.0	0.989	0.999	1.0	45
stim_tail_pain	0.9459	1.0	0.9722	0.9979	1.0	35

Supplementary Figure 17 | Training convergence curves for bilateral sensory stimulus classification in porcine models. Training (blue) and validation (orange) curves across 280 epochs for two metrics: left, cross-entropy loss; right, classification accuracy. Data represent a single training run on pooled data from n = 2 pigs (70% training, 15% validation, 15% test split), presented as a proof-of-concept feasibility demonstration.

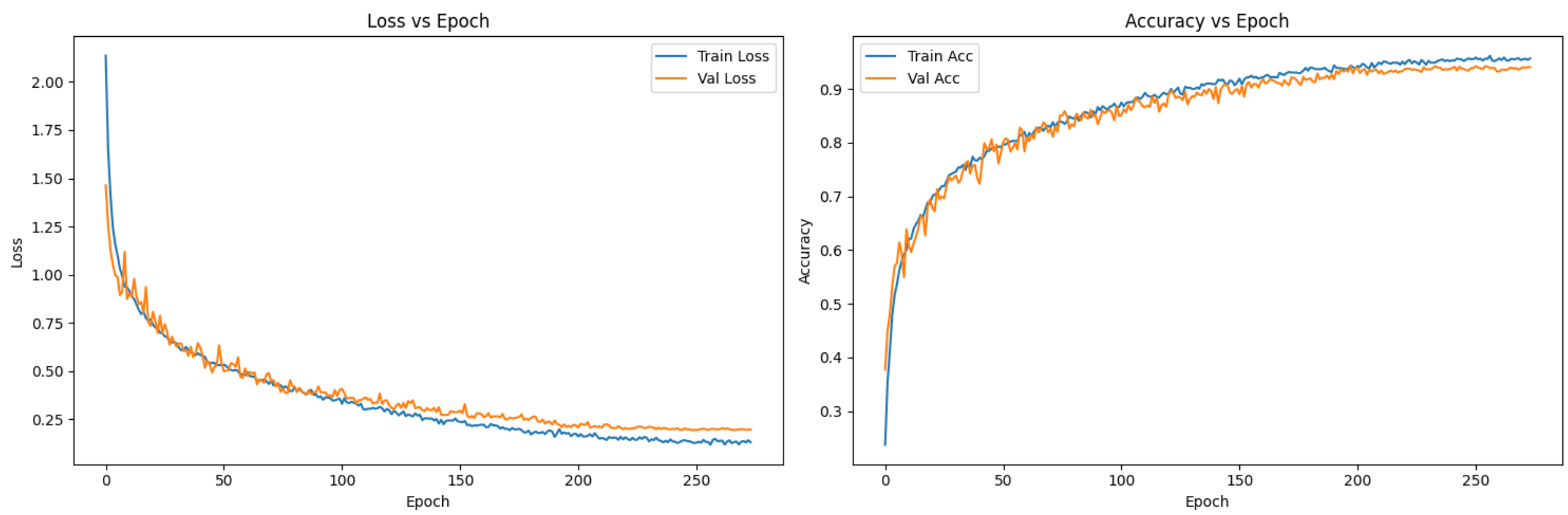

Supplementary Figure 18 | Radar plot of class-wise F1 scores for bilateral sensory stimulus classification in porcine models. Radar plot showing F1 scores for each of the five stimulus classes decoded by the LSTM-GRU classifier in anesthetised pigs, presented as a proof-of-concept feasibility demonstration (n = 2 pigs, total test samples n = 1,254). Radial axis ranges from 0 to 1.0, with concentric rings at 0.2 intervals. Classes shown clockwise: left leg top, left leg bottom, right leg top, right leg bottom, no stimulation. The shaded blue polygon represents the F1 score profile across all classes. Full per-class metrics are provided in Supplementary Fig. 19.

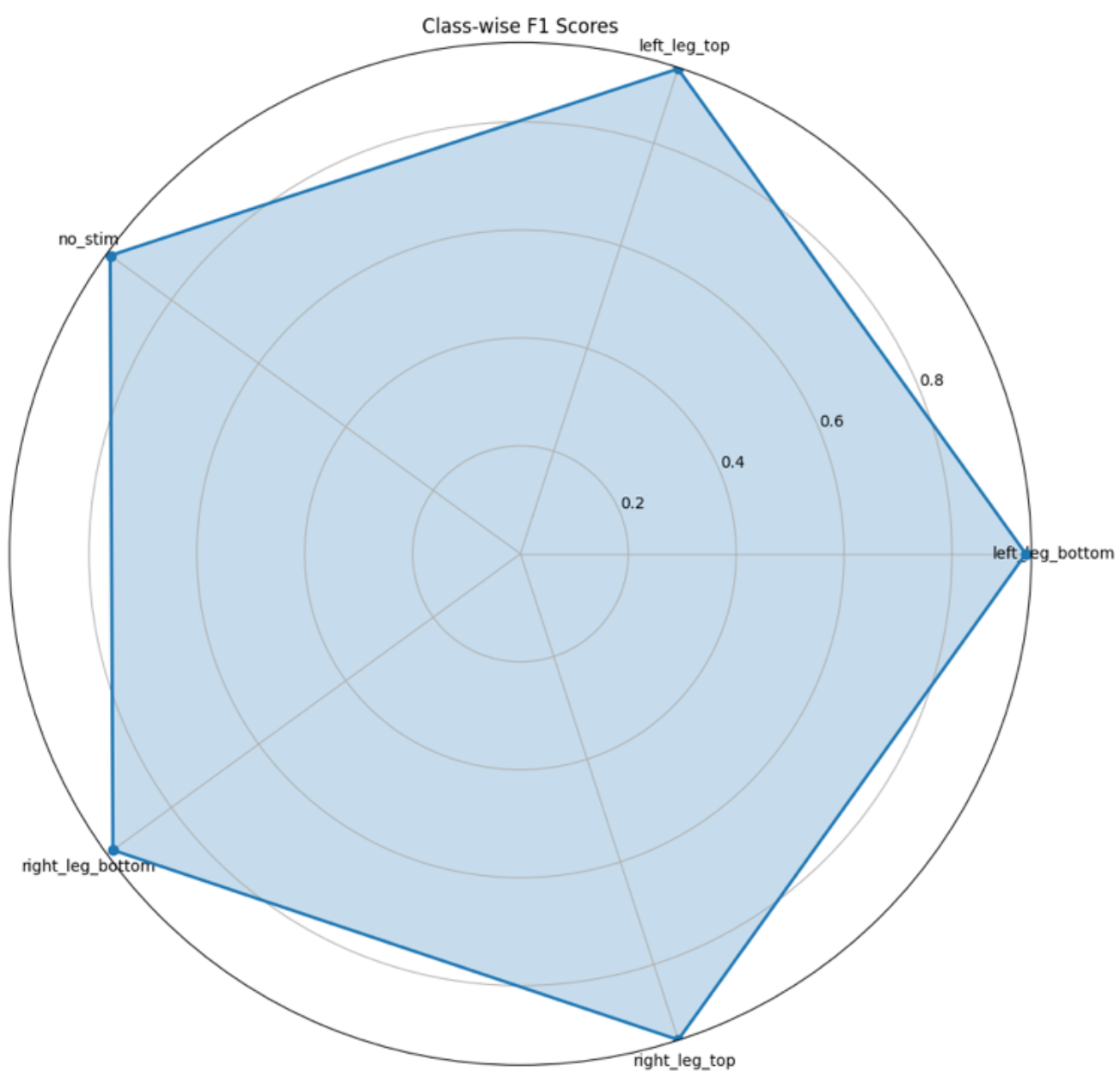

Supplementary Figure 19 | Performance metrics for sensory classification in porcine models. Table of per-class precision, recall/sensitivity, and F1 score for the five-class bilateral sensory stimulus classifier evaluated on held-out test data, presented as a proof-of-concept feasibility demonstration (n = 2 pigs, total test samples n = 1,254). Support column indicates the number of test samples per class (n = 251 per stimulation class, n = 250 no stimulation class. All metrics were computed on temporally held-out test data from the pooled dataset**.**

Feature	Precision	Recall/Sensitivity	F1 Score	Support
Left Leg Bottom	0.88	0.89	0.88	251
Left Leg Top	0.92	0.95	0.93	251
No Stim	0.93	0.86	0.89	250
Right Leg Bottom	0.9	0.88	0.89	251
Right Leg Top	0.89	0.92	0.9	251
**accuracy**			**0.9**	**1254**
**macro avg**	**0.9**	**0.9**	**0.9**	**1254**
**weighted avg**	**0.9**	**0.9**	**0.9**	**1254**

Supplementary Figure 20 | Training convergence curves for visceral afferent classification in porcine models. Training (blue) and validation (orange) curves across 120 epochs for two metrics: left, cross-entropy loss; right, classification accuracy. Data represent a single training run from n = 1 pig (70% training, 15% validation, 15% test split), presented as a proof-of-concept feasibility demonstration.

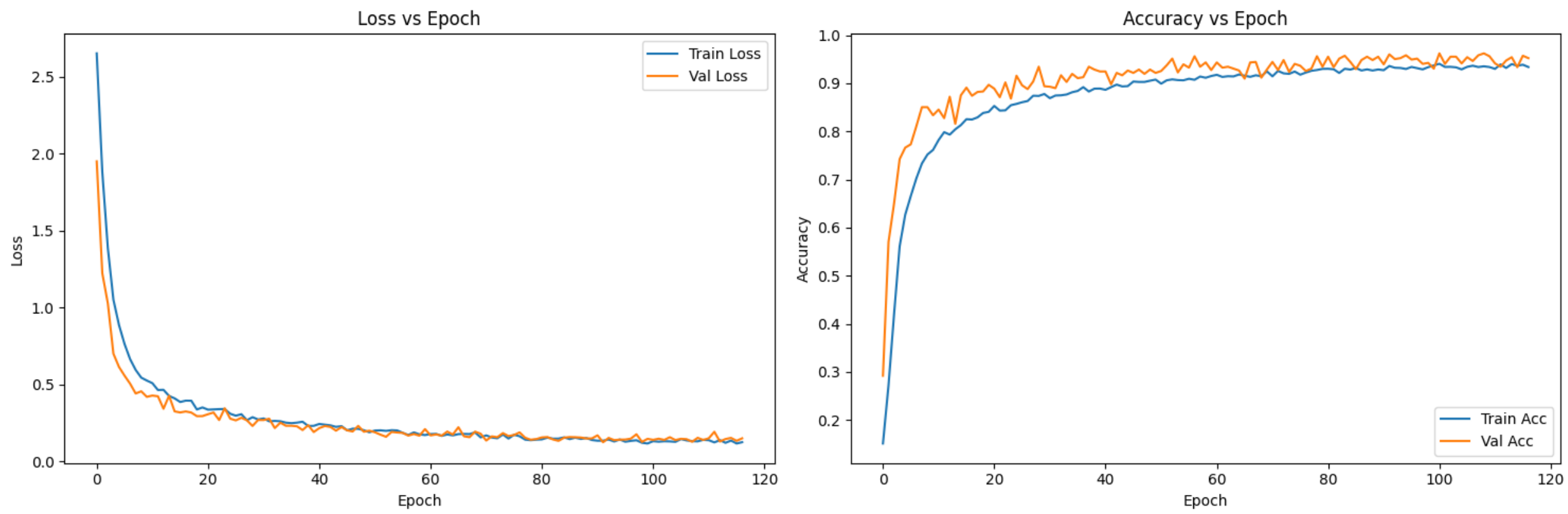

Supplementary Figure 21 | Radar plot of class-wise F1 scores for visceral afferent classification in porcine models. Radar plot showing F1 scores for each of the three classes decoded by the LSTM-GRU classifier in a single anesthetized pig, presented as a proof-of-concept feasibility demonstration (n = 1 pig, total test samples n = 533). Radial axis ranges from 0 to 1.0, with concentric rings at 0.2 intervals. Classes shown: rectal sphincter distension . no stimulation, and urethral sphincter distension. The shaded blue polygon represents the F1 score profile across all three classes. Full per-class metrics are provided in Supplementary Fig. 22.

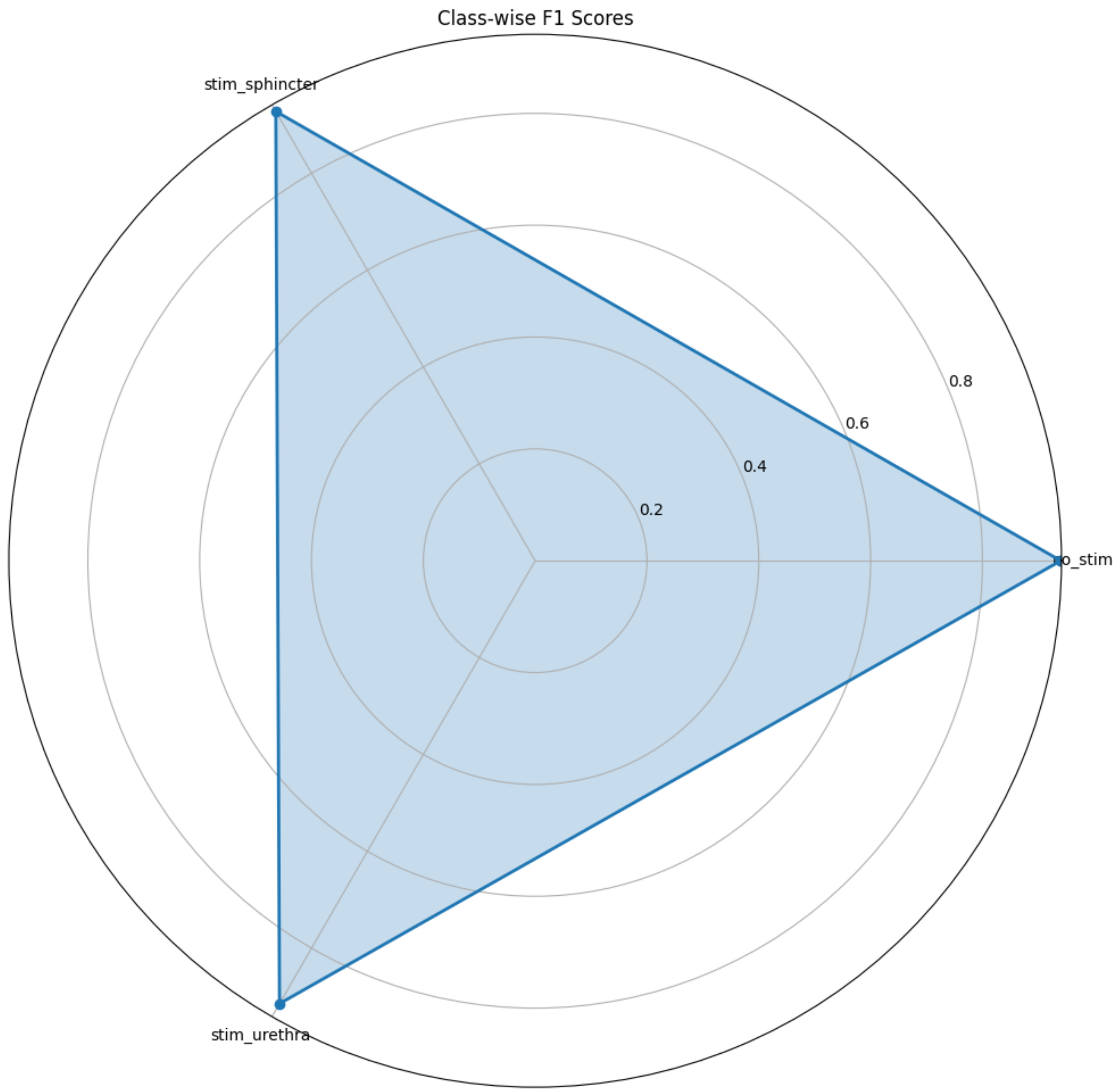

Supplementary Figure 22 | Performance metrics for visceral afferent classification in porcine models. Table of per-class precision, recall/sensitivity, and F1 score for the three-class visceral afferent classifier evaluated on held-out test data, presented as a proof-of-concept feasibility demonstration (n = 1 pig, total test samples n = 533). Support column indicates the number of test samples per class (no stimulation n = 267, rectal sphincter distension n = 133, urethral sphincter distension n = 133). All metrics were computed on temporally held-out test data.

Feature	Precision	Recall/Sensitivity	F1 Score	Support
no_stim	0.97	0.97	0.97	267
stim_sphincter	0.95	0.97	0.96	133
stim_urethra	0.96	0.95	0.96	133
**accuracy**			**0.96**	**533**
**macro avg**	**0.96**	**0.96**	**0.96**	**533**
**weighted avg**	**0.96**	**0.96**	**0.96**	**533**

Supplementary Figure 23 | Representative neural waveforms for source localization model training. Channel-averaged evoked responses recorded from the circumferential electrode array during intraspinal electrical stimulation across four conditions in a single anesthetized pig (n = 1 animal). Each panel shows amplitude (μV, y-axis) over time (ms, x-axis) across 17,500 ms recording windows. Colored traces represent the eight stimulation site positions: bottom left (light blue), bottom right (orange), bottom mid left (grey), bottom mid right (red), top mid left (purple), top mid right (brown), top left (pink), top right (dark grey). Stimulation waveforms are shown in the top right of each panel. a, Delay condition: biphasic pulse, 100 μA, 100 μs, 500 μs interpulse delay. b, High-amplitude condition: 500 μA, 100 μs. c, Extended-duration condition: 100 μA, 500 μs. d, Control condition: 100 μA, 100 μs.

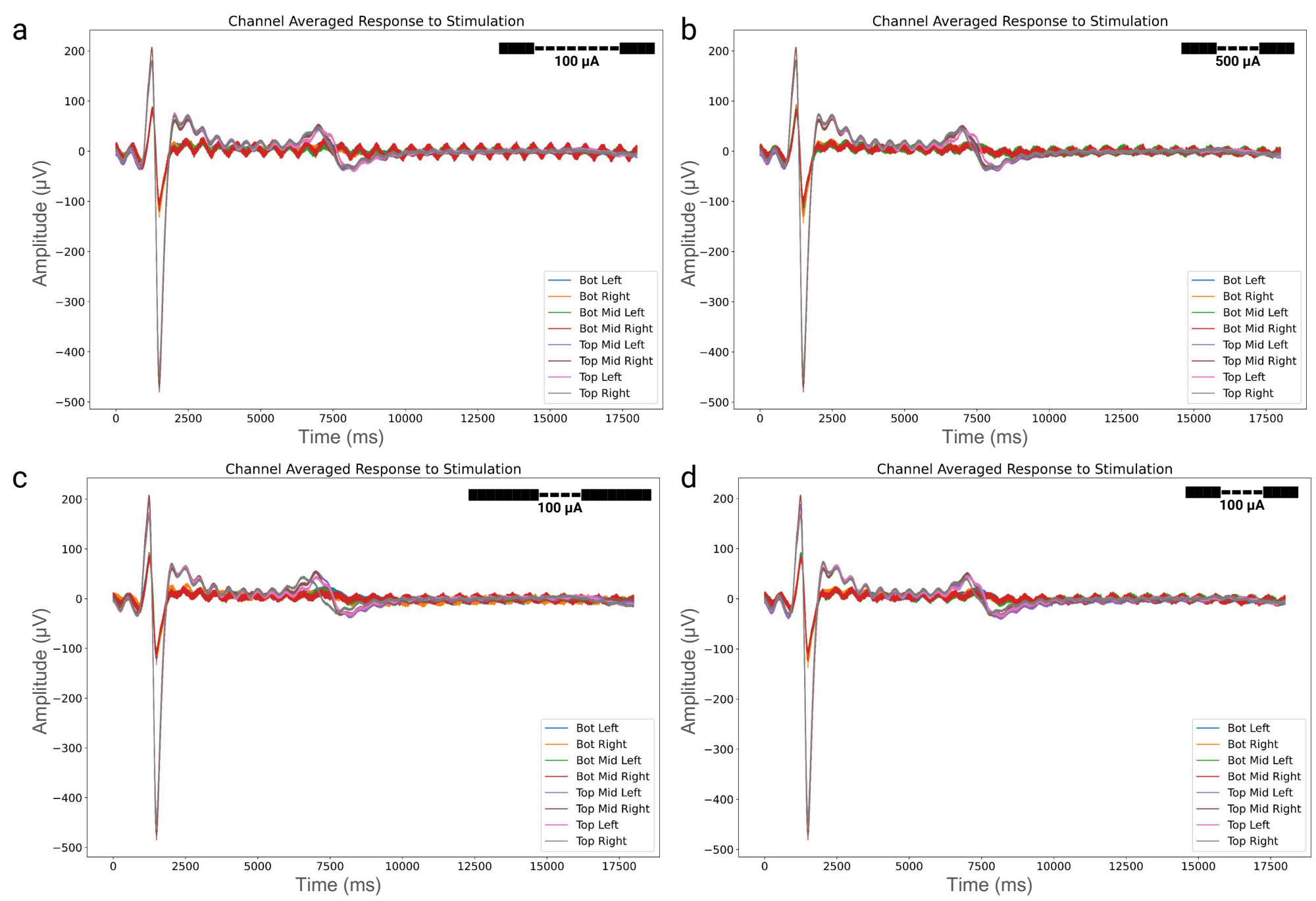

Supplementary Figure 24 | Afferent–efferent dominance index across the remaining four two-row implanted rats. Bar plots showing the net correlation coefficient (summed across all time) per electrode channel (channels 1–16, x-axis) for four additional animals (a–d, rats 2–5). Blue bars indicate efferent-dominant channels (positive values, rostral-to-caudal propagation); orange bars indicate afferent-dominant channels (negative values, caudal-to-rostral propagation). Y-axis ranges from −1 to +1.

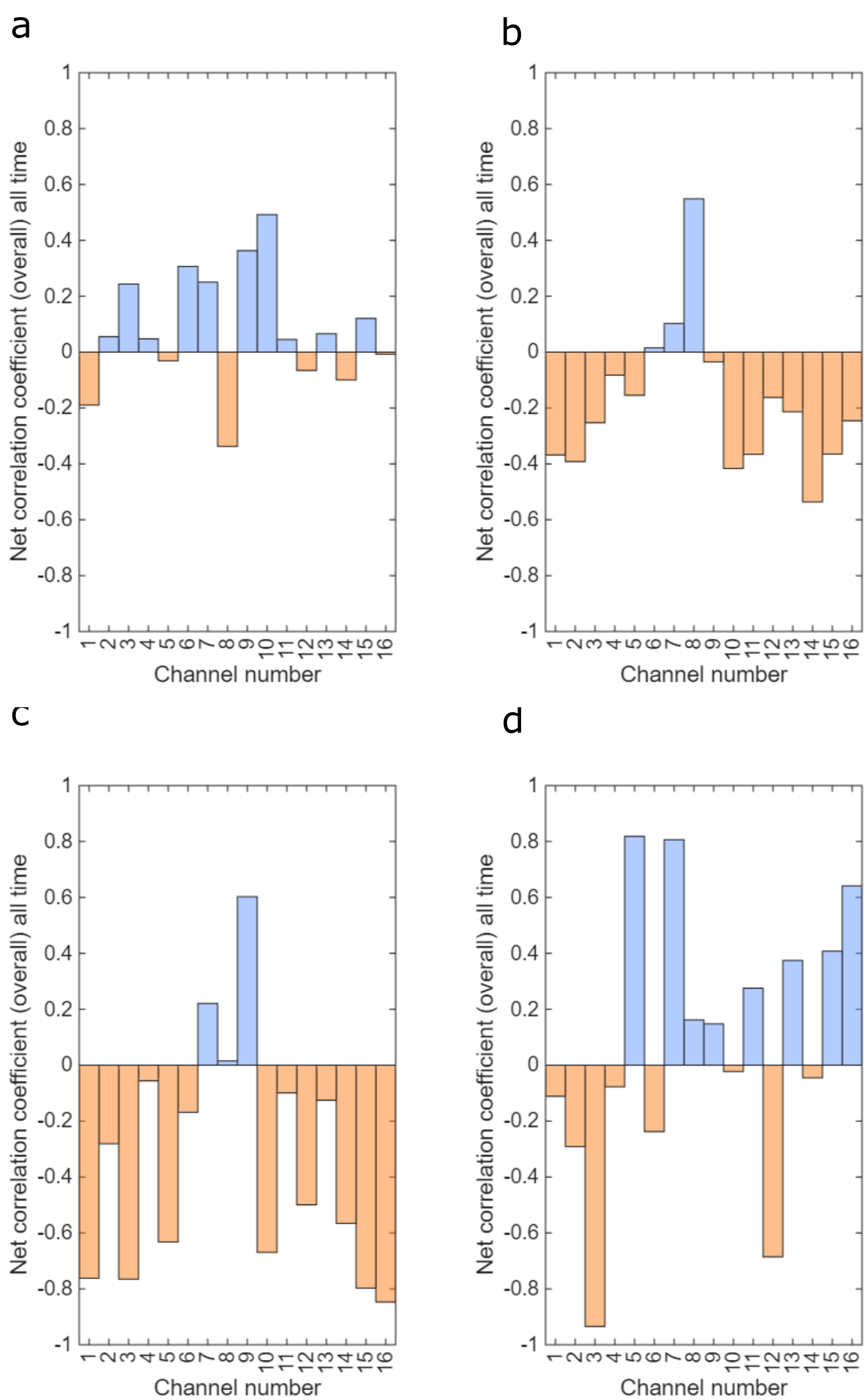

Supplement: Supplementary file 1 [file 41467_2026_75128_MOESM1_ESM.pdf]
