# Supplementary material for: Surface circumferential spinal cord recording in freely moving rodents
Source: Nat Commun. 2026 Jul 13;17:8610. doi: 10.1038/s41467-026-75128-z (PMC13487197; doi:10.1038/s41467-026-75128-z)
Supplement: Supplementary file 2 [file 41467_2026_75128_MOESM2_ESM.pdf]

Description of Additional Supplementary File

Supplementary Movie 1. Neural decoding of locomotor kinematics from circumferential spinal recordings. Top: Frame-by-frame comparison of ground truth skeletal trajectory reconstruction (left) derived from DeepLabCut markerless pose estimation versus neural decoder outcome from circumferential spinal electrode recordings (right). Bottom: Synchronized video of a freely moving rat with pose estimation landmarks overlaid, showing the naturalistic locomotor behavior being decoded in real time. Video playback at 0.3× original speed.
