# Supplementary material for: Surface circumferential spinal cord recording in freely moving rodents
Source: Nat Commun. 2026 Jul 13;17:8610. doi: 10.1038/s41467-026-75128-z (PMC13487197; doi:10.1038/s41467-026-75128-z)
Supplement: Supplementary file 4 [file 41467_2026_75128_MOESM5_ESM.pdf]

Peer Review File

Surface circumferential spinal cord recording in freely moving rodents

Corresponding Author: Professor Damiano G. Barone

This file contains all reviewer reports in order by version, followed by all author rebuttals in order by version.

Version 0:

Reviewer comments:

Reviewer #1

(Remarks to the Author)
The work aims to advance spinal cord interface technology by highlighting feasibility of multi-functional domains using a circumferential interface. Overall, the study is ambitious and technically sophisticated. The experimental design is well thought out, the methodological detail is extensive, and the data are generally convincing.
With revisions that clarify the conceptual framing, moderate claims by aligning them more closely with the presented data, and improve methodological transparency, this work has the potential to make a strong contribution to the field and to be suitable for publication. Below, I outline several points that require attention:

1) In the main text, the interface technology may appear to be introduced for the first time, whereas the used system has been previously reported and characterized (see DOI: 10.1126/sciadv.adl1230). The earlier work included validation in anesthetized rodents (demonstrations of motor and sensory readouts with source localization), assessment of tissue response using dummy implants in freely moving rats, and cadaveric human studies to evaluate surgical feasibility. The present manuscript extends this established platform by focusing on performance in freely moving rodent models and in anesthetized porcine preparations to explore feasibility in a larger anatomical context. The current text does not clearly outlines the boundary between the two works, and this should be addressed to better highlight the contribution of the present study. While the used interface concept is not entirely new, the shift toward information content, decoding, and spatial inference in freely moving animals represents a valuable contribution.
Previously published: Flexible circumferential bioelectronics to enable 360-degree recording and stimulation of the spinal cord, Science Advances, 2024, Vol 10, Issue 19, DOI: 10.1126/sciadv.adl1230,

2) Throughout the manuscript, recordings and implants are described as “chronic” and as supporting “long-term” spinal electrophysiology. However, implantation durations are limited to up to three days in rodents, and porcine experiments are performed acutely (anesthetized). Under commonly accepted definitions, 3-days correspond to acute or sub-acute studies and do not support long-term conclusions. Also, authors should explain why experiments were limited to three days and whether termination was driven by experimental design, or system limitations/failure.

3) Lines 160-162:“Across all cases, we observed no neurological deficits or tissue injury attributable to the implant, supporting both the safety and scalability of this circumferential design [12].”
The referenced work [12] refers to a previously published content. To which extent the present study contributes to additional data for this conclusion? If additional data addressing long-term the safety and scalability of this circumferential design are available, those should be provided as well.
Also, when discussing tissue–electrode interactions and cross-species implantation, it is important to discuss whether circumferential wrapping induced mechanical effects such as spinal cord compression or swelling, and whether such effects were observed in the freely moving rodent experiments and how they were assessed.

4) The impedance values (4.2 ± 5.9 kΩ vs 4.6 ± 6.0 kΩ) measured raise two points that need further clarification: at which frequency were these impedance measurements obtained (line 165-166)? And, the standard deviation exceeds the mean in both cases, indicating substantial inter-electrode variability. The authors should explain the sources of this variability and further elaborate why such dispersion is acceptable and does not compromise recording or stimulation performance.

5) Line 202-… “This analysis uncovered consistent propagation signatures, with velocity–time profiles that varied

systematically with electrode location (Fig. 3c)" and "This analysis revealed sustained directional preferences throughout locomotor epochs (Fig. 3d), which remained consistent across repeated trials."
This reports consistent propagation signatures and sustained directional preferences across repeated trials, however, Figure 3 appears to present data from a single electrode with multiple channels rather than across multiple electrodes or subjects. Please elaborate how is statistically relevant.

6) The quality of interpretation of the directional propagation and mapping analyses depends on accurate spatial localization of the electrodes. The manuscript should include a clearer description of how electrode positions are defined and maintained relative to spinal anatomy, how individual channel identities are preserved throughout implantation, and how their spatial locations are registered to anatomical targets.

7) Lines 234–238: The reported decoding performance is promising (R^2 up to 0.99, Pearson $r > 0.95$). It should be clarified whether these metrics are averaged across animals, features, and sessions, or reflect best-case performance.

8) Lines 296–302: The extension of the decoding framework to porcine models is valuable and demonstrates feasibility of applying the interface and analysis pipeline in a larger anatomical model. Yet, conclusions regarding cross-species generalization, scalability, and translational aspects should be rephrased cautiously given the statistically non-informative sample sizes (n = 1 and 2). These results more directly support recording feasibility across anatomical scales and serve as proof-of-concept demonstrations, rather than providing evidence of a clear translational pathway or robust autonomic modality discrimination.
Same applies to Lines 333–340, The reported classification accuracy (95–99%) is impressive, it seems as however, that these results are based on a single subject (n = 1) across four experimental conditions. While this supports feasibility, the interpretation should be limited to proof-of-concept performance under controlled conditions.
And in the discussion (Lines 386–391), the description of sensory and autonomic decoding, in pigs, is technically interesting, but the use of phrases such as "previously unreported exploration" and "significant breakthrough" should be moderated given the limited sample sizes and lack of longitudinal validation.

9) Lines 342–346: The inference that circumferential geometry enables discrimination of closely neighboring sites is plausible and supported by the confusion matrices. However, the claim that this resolution is "unattainable by single-plane or epidural devices" is comparative and would benefit from either supporting references/own data or more moderate phrasing.

10) Lines 355–359: The statements that the device "can access the full functional repertoire of the spinal cord" and "transforms the spinal cord into a neuroprosthetic hub" may benefit from moderation. While the study nicely demonstrates multimodal decoding under specific experimental conditions, these results are best interpreted as feasibility demonstrations rather than full access to spinal function.

11) Lines 379–381: the authors write that reported decoding performance "outperforms those reported from intraspinal electrodes", this reads rather definitive while not being sufficiently supported by the cited reference. The referenced intraspinal studies uses quite a different recording modality, signal types, and behavioral paradigms, which makes a direct performance comparison difficult. The nature of the comparison should be clarified. A more appropriate framing would be that the decoding performance is comparable to prior intraspinal work while achieved using a non-penetrating, circumferential interface.

Minor Comments
1)The reported sample size (n = 15) refers to the number of animals. To better assess readout stability, it would be helpful to include device-level or channel-level performance (e.g., for 16- or 32-channel arrays), indicating whether all channels were functional and whether any outliers were excluded. This information can be included in the Supplementary section.

2) Lines 416–423: The statement that spatial precision "elevates circumferential recording from a passive reading tool into a foundation for targeted therapeutic intervention" is overstated given the data presented (no therapeutic modulation, closed-loop stimulation, or functional outcome measures are shown). This should be rephrased in terms of improved spatial mapping and interpretive capability, or to clearly identify therapeutic applications as a future direction.

3)The image quality of some figures is poor and should be improved to enhance clarity and readability. This comment also applies to the Supplementary Figures and the caption content.

(Remarks on code availability)

Reviewer #2

(Remarks to the Author)
I read the study by El Hadwe and his colleagues with great interest. This is a well-written study about a novel circumferential spinal electrode array potentially useful as a multifunctional neuroprosthetic hub. The study was performed in rodents and pigs and decoded locomotor behaviour, classified a variety of sensory stimuli, and claimed to discriminate autonomic responses.

While this work is original and novel, I have several critical points which should be addressed.
Firstly, I think the translational impact for clinical populations, e.g. individuals with spinal cord injury, is questionable or at

least overstated at this time point. Acute SCI often presents with extended edema, extensive structural damage of white and grey matter of the cord and in more chronic stages with scar formation. All these processes will heavily limit intradural placement of an electrode array and the circumferential neural recordings. A logical next step should be the application in rodent and porcine models of SCI to study the feasibility in lesioned tissue. Please elaborate on these challenges.

Secondly, cardiovascular and respiratory autonomic control, e.g., sympathetic preganglionic neurons, is located at cervical and upper-thoracic spinal levels and therefore out of scope at this point. On the same note, I'd doubt that results presented in Figure 5 should be called "autonomic response" or interpreted as "access to spinal autonomic circuits". The observed spinal activity rather just highlights another strong sensory activation even when coming from visceral regions. Ballon inflation in urethral sphincter or rectum activates C-fibers and therefore represents another sensory mapping and not a "sphincter activation" in terms of motor sphincter control. I think the whole autonomic focus is exaggerated based on these findings and should be down tuned or omitted. Control of autonomic functions is complex and incorporates not only afferent signaling, but also supraspinal descending control from central autonomic networks, e.g. PAG, RVM etc., as well as spinal segmental function, sympathetic preganglionic neurons. The latter ones were not tested in this study.

The circumferential electrode array seems to cover only a small portion of the spinal cord (rostro-caudally). Maybe I missed this information, but how was the precise location selected and were nerve roots not affected by the placement?
In that regard, line 122 "...high-density microelectrode arrays that capture neural activity simultaneously across all spinal regions during naturalistic behavior." should be adapted.
The decoding of spinal locomotor control seems not specific. It was unclear throughout the manuscript if segmental spinal circuitries (e.g. CPG), descending motor tracts such as the CST / reticulospinal system, propriospinal projections or even sensory integration are assessed.

Specific comments

Abstract: the term "autonomic modalities" is vague and does not represent the findings. The modality was two times sensory stimulation due to balloon inflation (urethra vs. sphincter).

The seminal studies on improved upper limb (https://doi.org/10.1038/s41591-024-02940-9) and hemodynamic functon(doi: 10.1038/s41591-025-03614-w, doi: 10.1038/s41586-020-03180-w) using epidural stimulation by the Courtine group are missing in the introduction (e.g. line 107).

Figure 2d: in figure legend SNR is stated to be significantly different between movement and non-movement conditions. Please label accordingly in Figure.

Figure 3b is unclear. Do the authors herewith claim that only ascending and descending projections were assessed or also grey matter neurons (somata, missing in this figure)? This goes in the same direction as my specificity question above: which neurophysiological signals are measured (signaling along long projection neurons, segmental neuronal circuits)?

Line 404: "…making rostral spinal sampling a logical extension of necessary procedures...". Please explain in more detail. Necessary for what?

(Remarks on code availability)

Reviewer #3

(Remarks to the Author)
General Summary
The manuscript describes a thin flexible electrode array designed to wrap around the circumference of the spinal cord. The authors demonstrate the device's ability to record neural activity in freely moving rats over 3 days and to validate its scalability in acute porcine models. This work positions the implant design as a "middle ground" between non-invasive epidural leads, which lack spatial resolution and circumferential coverage, and penetrating intraspinal arrays, which risk tissue damage. The authors frame the device as a "multifunctional neuroprosthetic hub" capable of bidirectional interfacing. The work is a noteworthy proof of concept in spinal neural interfaces with the demonstration of motor intent, sensory and autonomic decoding, and will be of interest to those in the field. However, the claim of chronic viability, and the lack of neurological deficit or tissue injury in larger animal models, are not sufficiently substantiated and should be paired back.
Comments to the Authors
1. The data presented does not support the claim that the implant is chronically viable. In rats, the device was tested for only 3 days, and in pigs, it was tested only under anaesthesia. Why did the authors not evaluate implant performance, deficits, etc, for longer periods in rats, and in an awake/walking pig?
2. Why was there no evaluation of the foreign body response? Long-term implantation (weeks to months) is likely to generate significant FBR, which could limit the implant's ability to conform to mobile spinal tissue during movement, particularly in larger animals where spinal motion is greater than in a rat.
3. Can the authors elaborate on the risk profile for a circumferential implant design in a chronic model compared to a dorsal implant? How does the device design account for swelling, or dural scarring that might lead to compressive myelopathy over months/years? The authors could establish the necessity of the circumferential design by comparing decoding performance using only the dorsal subset of electrodes.

4. The authors state, “no neurological deficits or tissue injury attributable to the implant.” How was neurological deficit quantified? The presence of deficits in hind limb function, assessed with the BBB scale or similar, should be provided. Similarly, how was tissue injury quantified? Histology, showing the absence of compressive injury and the absence of an inflammatory response in the spinal cord, should be presented. How was the lack of deficit or tissue injury assessed in the pig models?
5. Was the decoding model tested on new animals that were not part of the training set (leave-one-out cross-validation), or only on held-out temporal data from the same animals? If the latter, the high accuracy may reflect overfitting to the specific gait idiosyncrasy of those five rats rather than a generalisable decoder of spinal motor control.
6. The authors presented confusion matrices for pig sensory and autonomic decoding separately. Were these separate models? The authors should address potential multimodal interference (misclassification between sensory/autonomic stimuli and motor control), as would be required in a clinical application.

Minor Comments
7. The authors state the implant can “capture neural activity simultaneously across all spinal regions”. This needs clarification - presumably, neural activity was only captured from regions under the implant. For the rodent model, it is not stated which spinal segment the implant was located over.
8. For sensory decoding, why did the authors not perform negative controls, e.g. sensory stimuli at the forelimbs, which would not be expected to be detected lower in the spinal cord?
9. “rostro–caudal signal propagation, a feature inaccessible to conventional planar arrays.” This statement needs clarification. Also note recent work by Hazelgrove et al. (2025, Scientific Reports), which reported the propagation of neural spikes in a dorsal planar array.
10. “Electrode impedances remained stable before and after implantation”. While there is no significant difference on a population level, the reported impedance values extend across orders of magnitude from 1 kΩ to 100 MΩ. What are these impedances? |Z| at 1 kHz? Why is there such a large range in the measured values? Methods should include input parameters for impedance measurement/configuration.

(Remarks on code availability)
No code review

Reviewer #4

(Remarks to the Author)
I co-reviewed this manuscript with one of the reviewers who provided the listed reports. This is part of the Nature Communications initiative to facilitate training in peer review and to provide appropriate recognition for Early Career Researchers who co-review manuscripts.

(Remarks on code availability)

Version 1:

Reviewer comments:

Reviewer #1

(Remarks to the Author)
The authors have provided extensive responses to the reviewer comments, and these efforts are appreciated. The revisions partially rely on rephrasing existing text rather than adding new data to substantiate the claims. As a result, some conclusions remain stronger than what is directly supported by the evidence, and some statements still read more like speculative points for future discussion than evidence-based findings. I outline below my comments, following the same numbering as in the initial request:
1) to address the comment, the authors have revised the text to include two lines “Building on our previously established device concept and framework...” and “...recordings during naturalistic behavior in freely moving animals.” While this is appreciated, the manuscripts novelty as presented still appears to shift between the underlying technology and the current experimental extension. Given that the core technology has already been described in a previous publication, a more substantial revision of the overall narrative would be preferable rather than limited rephrasing in the main text.
2) The authors note that 3 days does not represent a chronic experiment. However, the manuscript does not clearly identify the study as acute. Instead, the term “chronic” have been replaced in some places with “freely moving” or “recovery.” The manuscript should explicitly state in the abstract, main text, and discussion that this is concerns an acute experiment (3 days in freely moving rats, anesthetized porcine models). This information is currently mentioned only in the Results section.

Additionally, the manuscript should address why the study was limited to 3 days, or whether this was due to animal care considerations, device failure, housing constraints, or other factors. This point remains unaddressed.

3) The authors argue “ we note that across all implanted animals, no neurological deficits or gross tissue injury were observed during the study (3 days), as evidenced by animals resuming normal ambulatory behavior upon recovery” and to address the comment they revised the text to ” Across all cases, we observed no neurological deficits or tissue injury attributable to the implant during the duration of the study (3 days). (lines 163–164).

The authors’ statement that animals resumed normal ambulatory behavior upon recovery is presented without accompanying objective evidence. In the absence of histological analysis, quantitative neurological scoring, or direct assessment of tissue compression and edema, these observations remain insufficiently substantiated. As a result, the statements regarding safety and any implications for scalability are not adequately supported by the data presented. Additional quantitative evaluations would be necessary to justify these claims.
4) The authors provided the details on measurement frequency, instrument, and electrolyte setup, and add supplementary electrode-level impedance data.
The explanation for the large spread in the data remains somewhat speculative. While the authors list several possible causes, they do not clarify whether or how this variability affects performance over the implantation period. Although variability in impedance is not unusual, the magnitude of the observed spread may raise questions regarding the reproducibility of the electrode fabrication process.
5) no further comment.
6) no further comment.
7) no further comment.
8) The authors have moderated the language and now present the porcine findings as proof-of-concept. However, the manuscript still advances the translational pathway (and related discussions)relatively strongly, despite the limited porcine sample size (and the use of anesthetized animals).
9) no further comment.
10) no further comment.
11) no further comment.

Minor comments
Minor 1) The authors state that Supplementary Figure 4 demonstrates electrode-level stability across implantation with no significant population-level change. However, I am not convinced how Figure 4 (suppl.)help supporting this claim. As currently presented, the figure appears to be a collection of data points plotted together without clear structure, making it difficult to interpret the intended message. I encourage the authors to reconsider the presentation of this figure, ensuring that it conveys the key result and supports the stated conclusion.
Minor 2) no further comments.
Minor 3) no further comments.

(Remarks on code availability)

Reviewer #2

(Remarks to the Author)
The authors did a great job in addressing my comments and apadpted the manuscript accordingly. With the additionally provided information the article improved clarity.

(Remarks on code availability)

Reviewer #3

(Remarks to the Author)
The authors have addressed all questions raised in the peer review, and publication is recommended

(Remarks on code availability)

Reviewer #4

(Remarks to the Author)
I co-reviewed this manuscript with one of the reviewers who provided the listed reports. This is part of the Nature Communications initiative to facilitate training in peer review and to provide appropriate recognition for Early Career Researchers who co-review manuscripts.

(Remarks on code availability)

Open Access This Peer Review File is licensed under a Creative Commons Attribution 4.0 International License, which permits use, sharing, adaptation, distribution and reproduction in any medium or format, as long as you give appropriate credit to the original author(s) and the source, provide a link to the Creative Commons license, and indicate if changes were made.
In cases where reviewers are anonymous, credit should be given to 'Anonymous Referee' and the source.
The images or other third party material in this Peer Review File are included in the article's Creative Commons license, unless indicated otherwise in a credit line to the material. If material is not included in the article's Creative Commons license and your intended use is not permitted by statutory regulation or exceeds the permitted use, you will need to obtain permission directly from the copyright holder.
To view a copy of this license, visit https://creativecommons.org/licenses/by/4.0/

RESPONSE TO REVIEWERS

We have implemented the following color-coding system to respond to the reviewers' comments and explain the related changes in the manuscript:

REVIEWERS' COMMENTS: Original Comment
AUTHOR'S RESPONSE: Answer to each point
MANUSCRIPT MODIFICATION: Additions to the manuscript based on the addressed point

AUTHOR'S RESPONSE: We thank the Editor and Reviewers for their careful evaluation and constructive feedback. We believe that addressing all their comments significantly improved the clarity and rigor of our work. We have revised the manuscript to clarify the conceptual framing and novelty relative to our prior work, moderate claims to align more closely with the presented data and improve methodological transparency.

Reviewer #1:

The work aims to advance spinal cord interface technology by highlighting the feasibility of multi-functional domains using a circumferential interface. Overall, the study is ambitious and technically sophisticated. The experimental design is well thought out, the methodological detail is extensive, and the data are generally convincing.
With revisions that clarify the conceptual framing, moderate claims by aligning them more closely with the presented data, and improve methodological transparency, this work has the potential to make a strong contribution to the field and to be suitable for publication. Below, I outline several points that require attention:

1) In the main text, the interface technology may appear to be introduced for the first time, whereas the used system has been previously reported and characterized (see DOI: 10.1126/sciadv.adl1230). The earlier work included validation in anesthetized rodents (demonstrations of motor and sensory readouts with source localization), assessment of tissue response using dummy implants in freely moving rats, and cadaveric human studies to evaluate surgical feasibility. The present manuscript extends this established platform by focusing on performance in freely moving rodent models and in anesthetized porcine preparations to explore feasibility in a larger anatomical context. The current text does not clearly outlines the boundary between the two works, and this should be addressed to better highlight the contribution of the present study. While the used interface concept is not entirely new, the shift toward information content, decoding, and spatial inference in freely moving animals represents a valuable contribution.

Previously published: Flexible circumferential bioelectronics to enable 360-degree recording and stimulation of the spinal cord, Science Advances, 2024, Vol 10, Issue 19, DOI: 10.1126/sciadv.adl1230,

AUTHOR'S RESPONSE:
We have revised the final paragraph of the Introduction (Lines 125-134) to better delineate the contribution of the present study relative to our prior work. The revised text now explicitly states that we build upon the previously established device concept and framework and clarifies that the present work extends this platform to recovery recordings, decoding applications, and cross-species validation capabilities not demonstrated in the earlier study (1).

(1) DOI: 10.1126/sciadv.adl1230

MANUSCRIPT MODIFICATION:
Paragraph reads "Here, we demonstrate chronic, circumferential neural recordings from the spinal cord of freely moving rats, a capacity that has never been demonstrated before. Building on our previously established device concept and framework , we developed functional circumferential intradural interfaces that now achieve accurate motor intent extraction (R^2 = 0.97) and multimodal, plurisegmental sensory classification (94.4% accuracy) from surface spinal recordings during naturalistic behavior in freely moving animals. These interfaces, now scaled for larger species, reveal spatially distributed processing patterns that were previously inaccessible to conventional methods. This cross-species validation, from rodents to porcine models, establishes a translational pathway toward comprehensive closed-loop neuroprosthetics that integrate motor, sensory, and autonomic functions for clinical use."

Location in revised manuscript: (lines 125-134).

2) Throughout the manuscript, recordings and implants are described as “chronic” and as supporting “long-term” spinal electrophysiology. However, implantation durations are limited to up to three days in rodents, and porcine experiments are performed acutely (anesthetized). Under commonly accepted definitions, 3-days correspond to acute or sub-acute studies and do not support long-term conclusions. Also, authors should explain why experiments were limited to three days and whether termination was driven by experimental design, or system limitations/failure.

AUTHOR’S RESPONSE:
We agree that three days does not constitute chronic implantation under standard definitions. We apologise for this. We have replaced "chronic" with "freely moving" throughout the manuscript.

MANUSCRIPT MODIFICATION:
“Freely moving” replaced “chronic”
Location in revised manuscript: Throughout (Abstract, Introduction, Results, Discussion).

3) Lines 160-162:“Across all cases, we observed no neurological deficits or tissue injury attributable to the implant, supporting both the safety and scalability of this circumferential design [12].”
The referenced work [12] refers to a previously published content. To which extent the present study contributes to additional data for this conclusion? If additional data addressing long-term the safety and scalability of this circumferential design are available, those should be provided as well.
Also, when discussing tissue–electrode interactions and cross-species implantation, it is important to discuss whether circumferential wrapping induced mechanical effects such as spinal cord compression or swelling, and whether such effects were observed in the freely moving rodent experiments and how they were assessed.

AUTHOR’S RESPONSE:
We agree that a comprehensive biocompatibility assessment was conducted in our previously cited work. However, we note that across all implanted animals, no neurological deficits or gross tissue injury were observed during the study (3 days), as evidenced by animals resuming normal ambulatory behavior upon recovery. We have revised the manuscript to make the time-limited nature of this observation explicit: "Across all cases, we observed no neurological deficits or tissue injury attributable to the implant during the duration of the study (3 days).

MANUSCRIPT MODIFICATION:
Text read” Across all cases, we observed no neurological deficits or tissue injury attributable to the implant during the duration of the study (3 days).
Location in revised manuscript: (lines 163–164).

4) The impedance values (4.2 ± 5.9 kΩ vs 4.6 ± 6.0 kΩ) measured raise two points that need further clarification: at which frequency were these impedance measurements obtained (line 165-166)? And, the standard deviation exceeds the mean in both cases, indicating substantial inter-electrode variability. The authors should explain the sources of this variability and further elaborate why such dispersion is acceptable and does not compromise recording or stimulation performance.

AUTHOR’S RESPONSE:

Impedance measurements were obtained at 1 kHz using an AutoLAB potentiostat in phosphate-buffered saline with a platinum counter and reference electrode, as now detailed in Methods. The wide impedance distribution potentially arises from PEDOT:PSS coating thickness variations, microscale fabrication geometry differences, and variability in tissue-electrode contact area. In response to a related to a later comment we have added Supplementary Figure 4 showing electrode-by-electrode impedance stability for all 15 implanted devices (n = 480 paired electrodes). Individual electrodes maintained stable impedance characteristics across implantation.

MANUSCRIPT MODIFICATION:
Text now reads: "Impedance measurements were obtained at 1 kHz using an AutoLAB potentiostat in phosphate-buffered saline, with a platinum counter/reference electrode. Post-implantation measurement(s) where done using the Intan RHX hardware."

Location in revised manuscript: Methods, "Electrical Characterization" (lines 616-618); Supplementary Figure 4 added.

5) Line 202-… "This analysis uncovered consistent propagation signatures, with velocity–time profiles that varied systematically with electrode location (Fig. 3c)" and "This analysis revealed sustained directional preferences throughout locomotor epochs (Fig. 3d), which remained consistent across repeated trials."
This reports consistent propagation signatures and sustained directional preferences across repeated trials, however, Figure 3 appears to present data from a single electrode with multiple channels rather than across multiple electrodes or subjects. Please elaborate how is statistically relevant.

AUTHOR'S RESPONSE:
We thank the reviewer for this important observation. We have now performed the directional propagation analysis across all five rats implanted with two-row devices. Figure 3 presents data from a single representative animal across three independent locomotor sessions to illustrate the approach in detail; consistent afferent-efferent dominance patterns with lateral symmetry were observed across all five animals and are now presented in Supplementary Figure 24. The figure legend has been updated accordingly to clarify the representative nature of the main figure.

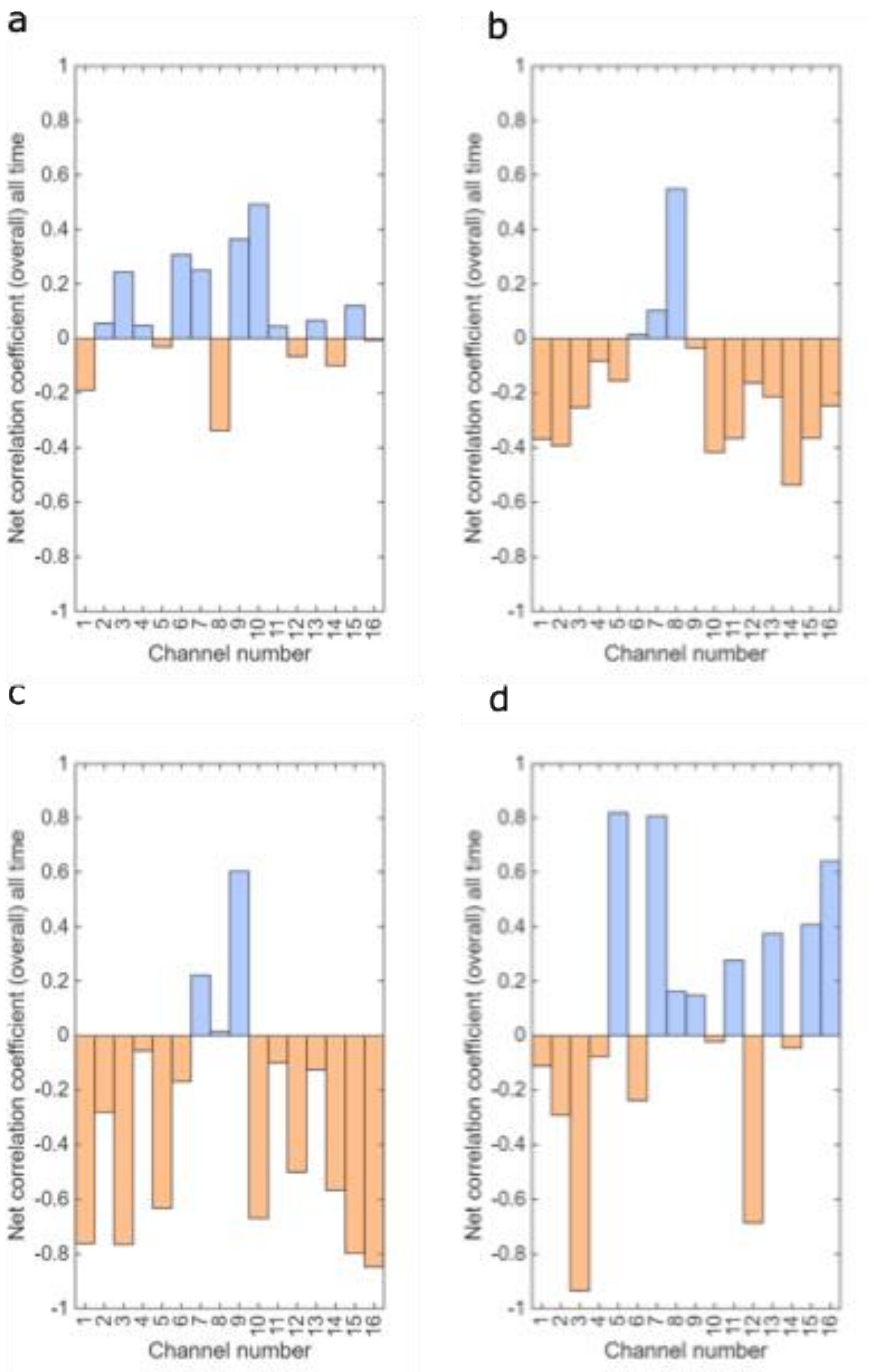

MANUSCRIPT MODIFICATION:

Figure 3 legend updated to explicitly identify the data as from a single representative animal. Supplementary Figure 24 added showing equivalent afferent-efferent dominance index plots for all remaining four two-row implanted rats.

Location in revised manuscript: Figure 3 legend; Supplementary Figure 24 added.

6) The quality of interpretation of the directional propagation and mapping analyses depends on accurate spatial localization of the electrodes. The manuscript should include a clearer description of how electrode positions are defined and maintained relative to spinal anatomy, how individual channel identities are preserved throughout implantation, and how their spatial locations are registered to anatomical targets.

AUTHOR'S RESPONSE:
We thank the reviewer for this important point. Electrode spatial localization was achieved through the following approach: At implantation, we recorded the rotational orientation of the device relative to anatomical landmarks, specifically noting which electrode pairs were positioned on the dorsal surface in relation to the central descending artery. For example, in Figure 1 B, electrode pairs 5 and 6 are shown in the dorsal position. Once positioned, the device was stabilized using cyanoacrylate adhesive applied to the dura, which we tested to ensure that device rotation around the cord could not occur. The intrinsic adhesiveness of the parylene-C substrate to the spinal cord surface provided additional stabilization. At the terminal procedure, we confirmed that the device remained in its original position relative to the anatomical landmarks. Electrode channel identity was preserved throughout implantation using a standardized mapping protocol. We developed a custom script that tracked electrode positions from the device through the flexible flat cable (FFC), intermediate connector PCB (known architecture), and recording unit channels, ensuring that the spatial arrangement of electrodes was maintained during visualization and analysis. We have added clarification of this approach to the Methods section.

MANUSCRIPT MODIFICATION:
Text reads "…The device was stabilized with cyanoacrylate adhesive applied to the dura, preventing rotation around the spinal cord. The intrinsic adhesiveness of the parylene-C substrate to the spinal cord surface provided additional stabilization. Device position was verified at the terminal procedure to confirm no displacement had occurred relative to the documented anatomical landmarks.
Electrode channel identity was preserved throughout the recording chain using a standardized mapping protocol. Mapping of electrode locations from devices to recording unit channels was performed using a custom script that tracked electrode positions through the entire signal path: from the device electrode array through the FFC, the FFC to the zero-insertion-force (ZIF) connector on the intermediate connector PCB, the intermediate PCB to the recording unit connector, and finally to the recording unit channels. The script processed an array representing the original spatial positions of the electrodes on the device and mapped them to the corresponding recording unit channels, ensuring that the physical arrangement of electrodes was maintained during visualization and analysis."

Location in revised manuscript: Methods, subsection "Electrode mapping" (lines 726-738).

7) Lines 234–238: The reported decoding performance is promising (R^2 up to 0.99, Pearson r >

0.95). It should be clarified whether these metrics are averaged across animals, features, and sessions, or reflect best-case performance.

AUTHOR'S RESPONSE:
We thank the reviewer for seeking this clarification. The reported $R^2 = 0.99$ represents the best-performing feature (ankle position) when the model was tested on held-out data. The decoder was trained on pooled data from all five rats across multiple sessions, and performance metrics reflect testing on temporally held-out data from these same animals. Performance varied by feature: ankle position achieved $R^2 = 0.99$, while joint angles consistently exceeded $R^2 = 0.93$. All 22 biomechanical features exhibited strong correlations with ground truth trajectories (Pearson $r > 0.95$, $p < 0.001$). Complete performance metrics for all tracked and calculated features are provided in Supplementary Figure 6.

MANUSCRIPT MODIFICATION:
Text reads "The LSTM–GRU network architecture, trained on pooled data from all five rats, achieved robust decoding across the 22 biomechanical features when tested on held-out data. Ankle position achieved the highest accuracy ($R^2 = 0.99$, MAE = 0.09), representing the best-performing feature. All features exhibited strong correlations with ground truth trajectories (Pearson $r > 0.95$, $p < 0.001$, $n = 5$), as shown in scatter plots and trajectory reconstructions (Fig. 4b,e; Supplementary Figs. 6–8)."

Location in revised manuscript: Results, subsection "Decoding motor intent with delta-band dominance" (lines 239–242).

8) Lines 296–302: The extension of the decoding framework to porcine models is valuable and demonstrates feasibility of applying the interface and analysis pipeline in a larger anatomical model. Yet, conclusions regarding cross-species generalization, scalability, and translational aspects should be rephrased cautiously given the statistically non-informative sample sizes (n = 1 and 2). These results more directly support recording feasibility across anatomical scales and serve as proof-of-concept demonstrations, rather than providing evidence of a clear translational pathway or robust autonomic modality discrimination.
Same applies to Lines 333–340, The reported classification accuracy (95–99%) is impressive, it seems as however, that these results are based on a single subject (n = 1) across four experimental conditions. While this supports feasibility, the interpretation should be limited to proof-of-concept performance under controlled conditions.
And in the discussion (Lines 386–391), the description of sensory and autonomic decoding, in pigs, is technically interesting, but the use of phrases such as "previously unreported exploration" and "significant breakthrough" should be moderated given the limited sample sizes and lack of longitudinal validation.

AUTHOR'S RESPONSE:
We have revised the manuscript throughout to moderate claims and accurately characterize these findings as feasibility demonstrations that support scalability to larger anatomical models, rather than definitive evidence of translational readiness.
We agree with the reviewer that the porcine experiments (n = 1–2) represent proof-of-concept demonstrations under controlled conditions. However, we note that the primary technological advance occurs precisely at this proof-of-concept stage: demonstrating that circumferential surface recordings can capture and decode functionally relevant signals across species and anatomical scales represents a critical first step toward clinical translation. These initial

demonstrations establish the foundation for more extensive validation studies with larger cohorts and longer implantation durations.

MANUSCRIPT MODIFICATION:

Specifically, we have:

1. Revised the Results section to explicitly state sample sizes and frame findings as proof-of-concept demonstrations
2. Moderated Discussion language, removing phrases such as "significant breakthrough" and "previously unreported exploration"
3. Clarified that these demonstrations establish feasibility across anatomical scales but require further validation in larger cohorts to quantify performance characteristics and translational viability

Location in revised manuscript: Results, subsection "Neural decoding differentiates sensory and autonomic responses across species" (lines 293–294); Results, subsection "Precise intraspinal source localization via circumferential recording" (lines 343-345; 352–357); Discussion (lines 365–368, 388-392, 396-398, 401-406, 408-409, 413-418, 443-446, 456-460).

9) Lines 342–346: The inference that circumferential geometry enables discrimination of closely neighboring sites is plausible and supported by the confusion matrices. However, the claim that this resolution is “unattainable by single-plane or epidural devices” is comparative and would benefit from either supporting references/own data or more moderate phrasing.

AUTHOR’S RESPONSE:

We agree with the reviewer that the original phrasing was overly definitive without direct experimental comparison. We have revised the statement to more accurately reflect the current state of the literature, noting that such resolution has not been demonstrated using single-plane or epidural devices, rather than claiming it is unattainable.

MANUSCRIPT MODIFICATION:

Paragraph now reads " The ability to discriminate between closely neighboring sites demonstrates that the circumferential geometry provides complementary perspectives on activation spread across the dorsal, lateral, and ventral surfaces. By sampling simultaneously from all territories, the interface captures spatially distributed activation patterns that enable reconstruction of a spatial fingerprint for intraspinal sources ; a capability that has not been demonstrated using single-plane or epidural devices."

Location in revised manuscript: " (lines 343–345).

10) Lines 355–359: The statements that the device “can access the full functional repertoire of the spinal cord” and “transforms the spinal cord into a neuroprosthetic hub” may benefit from moderation. While the study nicely demonstrates multimodal decoding under specific experimental conditions, these results are best interpreted as feasibility demonstrations rather than full access to spinal function.

AUTHOR’S RESPONSE:

We have moderated the language to accurately reflect that our work demonstrates multimodal decoding capabilities under specific experimental conditions rather than comprehensive access to all spinal functions.

MANUSCRIPT MODIFICATION:
Paragraph now reads “We demonstrate that a single, ultra-thin circumferential intradural device can decode motor, sensory, and autonomic signals while resolving spatial sources, all achieved without penetrating or disrupting neural tissue. This approach positions the spinal cord as a target for bidirectional, multifunctional neural interfacing. ”

Location in revised manuscript: Discussion, opening paragraph (lines 352–357).

11) Lines 379–381: The authors write that reported decoding performance “outperforms those reported from intraspinal electrodes.” This reads rather definitive, while not being sufficiently supported by the cited reference. The referenced intraspinal studies uses quite a different recording modality, signal types, and behavioral paradigms, which makes a direct performance comparison difficult. The nature of the comparison should be clarified. A more appropriate framing would be that the decoding performance is comparable to prior intraspinal work while achieved using a non-penetrating, circumferential interface.

AUTHOR’S RESPONSE:
We have revised the statement to indicate that our decoding performance is comparable to prior intraspinal work while being achieved using a non-penetrating, circumferential interface.

MANUSCRIPT MODIFICATION:
Location in revised manuscript: Discussion (lines 396–406).
Paragraph now reads “Our reported decoding capabilities are comparable to those reported with intraspinal electrodes, achieved using a non-penetrating, circumferential interface, presumably due to the nature of the coverage. Most importantly, for both directional mapping and locomotion decoding, granular channel analysis revealed spatial patterns that aligned with known spinal cord anatomy when mapped to the circumferential geometry ”

Minor Comments
1)The reported sample size (n = 15) refers to the number of animals. To better assess readout stability, it would be helpful to include device-level or channel-level performance (e.g., for 16- or 32-channel arrays), indicating whether all channels were functional and whether any outliers were excluded. This information can be included in the Supplementary section.

AUTHOR’S RESPONSE:
The reported sample size (n = 15) refers to the number of animals implanted across the study. To better assess device- and channel-level performance, we have added Supplementary Figure 4, which shows electrode-by-electrode impedance measurements for all 15 implanted devices.

Supplementary Figure 4 presents paired impedance measurements for all 480 electrodes (15 animals × 32 channels), demonstrating electrode-level stability across implantation with no significant population-level change.

Functional analyses (motor decoding, sensory classification) were performed on recordings with adequate signal quality, as determined by signal-to-noise ratio during experimental sessions. No channels were excluded based on arbitrary impedance thresholds; rather, recording quality was assessed during live sessions based on signal characteristics.

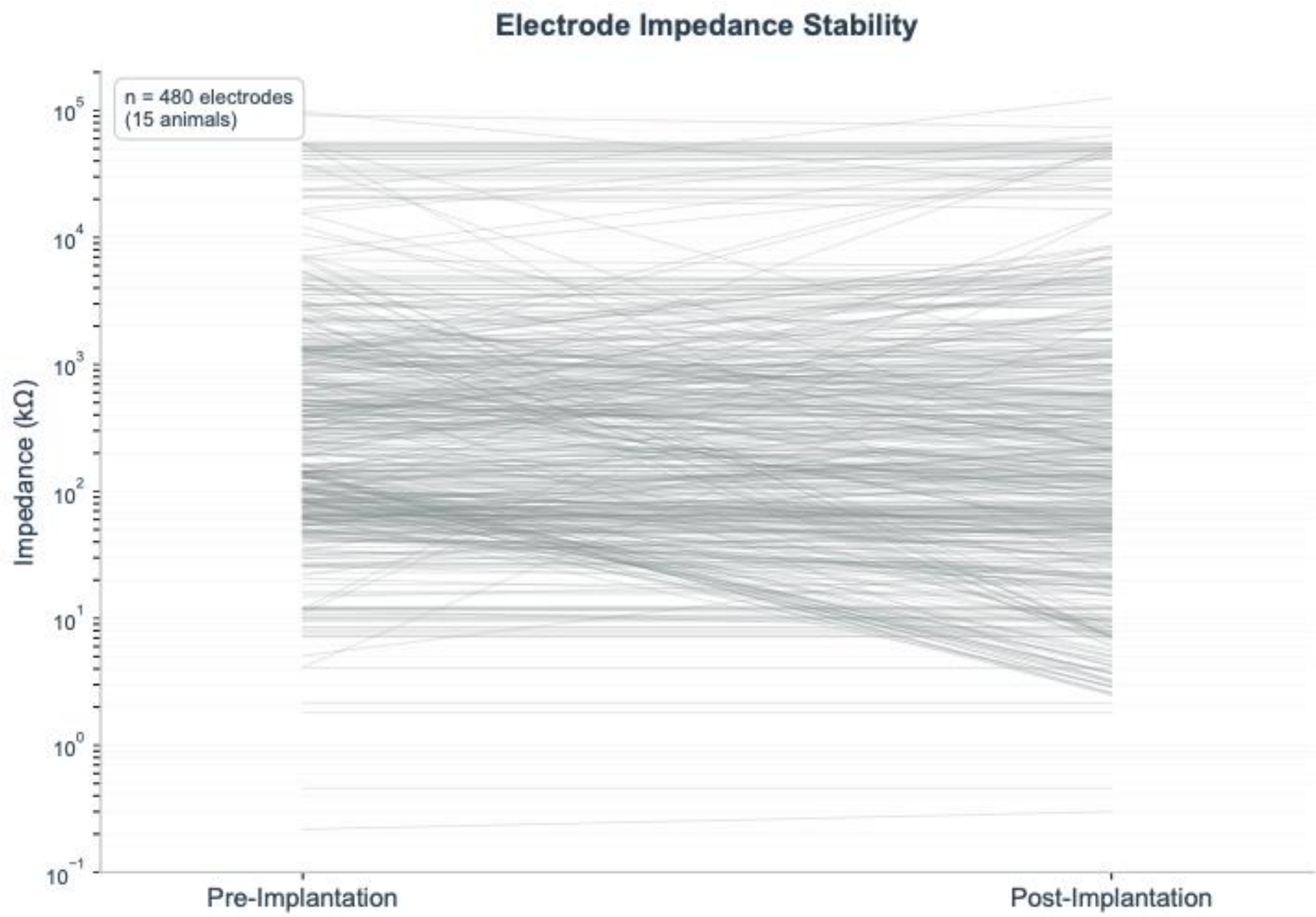

MANUSCRIPT MODIFICATION:
Supplementary Figure 4 added

2) Lines 416–423: The statement that spatial precision "elevates circumferential recording from a passive reading tool into a foundation for targeted therapeutic intervention" is overstated given the data presented (no therapeutic modulation, closed-loop stimulation, or functional outcome measures are shown). This should be rephrased in terms of improved spatial mapping and interpretive capability, or to clearly identify therapeutic applications as a future direction.

AUTHOR'S RESPONSE:
We have revised the text to focus on the demonstrated spatial-mapping capability and to identify therapeutic applications as a future direction.

MANUSCRIPT MODIFICATION:
Location in revised manuscript: Discussion (lines 443–446).
Paragraph now reads "……This spatial mapping capability provides insight into the origins of intraspinal neural activity. In future applications, such spatial information could potentially guide targeted stimulation of defined parenchymal targets."

3)The image quality of some figures is poor and should be improved to enhance clarity and readability. This comment also applies to the Supplementary Figures and the caption content.

AUTHOR’S RESPONSE:
All main figures were originally created as vectorized files. The reduced quality may have resulted from file compression during the initial submission process. In the revised submission, we will ensure that all figures are uploaded in their original vectorized PDF format to maintain maximum clarity and readability. We will also review and enhance the quality of all Supplementary Figures.

MANUSCRIPT MODIFICATION:
N/A

Reviewer #2:

I read the study by El Hadwe and his colleagues with great interest. This is a well-written study about a novel circumferential spinal electrode array potentially useful as a multifunctional neuroprosthetic hub. The study was performed in rodents and pigs and decoded locomotor behaviour, classified a variety of sensory stimuli, and claimed to discriminate autonomic responses.

While this work is original and novel, I have several critical points which should be addressed.

- Firstly, I think the translational impact for clinical populations, e.g. individuals with spinal cord injury, is questionable or at least overstated at this time point. Acute SCI often presents with extended edema, extensive structural damage of white and grey matter of the cord and in more chronic stages with scar formation. All these processes will heavily limit intradural placement of an electrode array and the circumferential neural recordings. A logical next step should be the application in rodent and porcine models of SCI to study the feasibility in lesioned tissue. Please elaborate on these challenges.

AUTHOR'S RESPONSE:

We thank the reviewer for raising this important translational consideration. The feasibility of intradural device placement in acute SCI with edema is directly supported by clinical evidence from duroplasty procedures. A multicenter randomized controlled trial (DISCUS, Saadoun et al., Trials 2023) (1) demonstrated that intradural surgical access is safe and feasible within 72 hours of acute cervical SCI, precisely during the period of maximal cord swelling and edema. In that study, duroplasty (dural opening with patch placement). Exploratory studies preceding DISCUS showed no persistent CSF leak, wound infection, or neurological worsening following acute intradural intervention during the edematous phase.

(1) doi: 10.1186/s13063-023-07454-2

MANUSCRIPT MODIFICATION:

Location in revised manuscript: Discussion (lines 456–460).

Text now reads "…. Importantly, circumferential recording targets preserved cord segments rostral or caudal to the lesion rather than the injury epicenter, where edema and scarring are most severe; clinical evidence from intradural duroplasty procedures confirms surgical feasibility even in the acute post-injury period [48] . Moreover, individual anatomical variability in spinal cord anatomy and level of injury may also…."

- Secondly, cardiovascular and respiratory autonomic control, e.g., sympathetic preganglionic neurons, is located at cervical and upper-thoracic spinal levels and therefore out of scope at this point. On the same note, I'd doubt that results presented in Figure 5 should be called "autonomic response" or interpreted as "access to spinal autonomic circuits". The observed spinal activity rather just highlights another strong sensory activation even when coming from visceral regions. Ballon inflation in urethral sphincter or rectum activates C-fibers and therefore represents another sensory mapping and not a "sphincter activation" in terms of motor sphincter control. I think the whole autonomic focus is exaggerated based on these findings and should be down tuned or omitted. Control of autonomic functions is complex and incorporates not only afferent signaling, but also supraspinal descending control from central autonomic networks, e.g. PAG, RVM etc., as well as spinal segmental function, sympathetic preganglionic neurons. The latter ones were not tested in this study.

AUTHOR'S RESPONSE:
We thank the reviewer for this important clarification. We agree that our porcine experiment detected afferent visceral sensory signals (from urethral and rectal sphincter distension) rather than sympathetic preganglionic neuron activity. However, these visceral afferents constitute the sensory limb of spinal autonomic reflex arcs and carry information relevant to autonomic function to supraspinal and spinal autonomic centers. We have clarified in the revised manuscript that we demonstrate discrimination of visceral afferent inputs relevant to autonomic function rather than claiming direct access to efferent autonomic control pathways.

For clinical applications, the ability to decode visceral afferent activity is directly relevant to monitoring and closed-loop control of bladder and bowel function in SCI patients, where both afferent sensation and efferent control are commonly impaired. We agree that comprehensive autonomic neuroprosthetics would ultimately require interfacing with both afferent and efferent limbs of autonomic pathways, and we have moderated our claims accordingly.

MANUSCRIPT MODIFICATION:
Location in revised manuscript: Results, subsection "Neural decoding differentiates sensory and autonomic responses across species" (lines 312-317); Discussion (lines 413-418).

Paragraph now reads "We then turned to visceral afferent decoding, which provides autonomic-relevant information for bladder and bowel function monitoring, as an exploratory proof-of-concept demonstration. In a single porcine experiment, the network achieved near-perfect classification of rest, urethral sphincter distension, and rectal sphincter distension ($F1 \geq 0.97$, $n = 1$), …."

AND

Visceral afferent signal discrimination in pigs, while demonstrated in a single proof-of-concept experiment, suggests that surface electrodes may provide access to autonomic-relevant sensory pathways; a capability that, if validated in larger cohorts, could address a critical gap in current neurorehabilitation practice. Our proof-of-concept demonstrations in porcine models suggest that the principles of circumferential intradural recording may scale to larger, clinically relevant dimensions.

- The circumferential electrode array seems to cover only a small portion of the spinal cord (rostro-caudally). Maybe I missed this information, but how was the precise location selected and were nerve roots not affected by the placement?

AUTHOR'S RESPONSE:
The device was indeed designed to cover a limited rostro-caudal extent. The implantation level was selected as the thoracolumbar junction in each species.

The device width (rostro-caudal dimension) was calculated based on anatomical measurements to ensure it fit between two adjacent dorsal root exit zones without contacting or compressing the nerve roots. Specifically, the 1.7 mm width (two-row device) and 1.4 mm width (one-row device) were selected to be smaller than the inter-root distance at the target spinal level in both rats and pigs, ensuring that implantation did not damage exiting nerve roots or interfere with their function. We have added this information to the Methods section.

MANUSCRIPT MODIFICATION:
Location in revised manuscript: Methods, subsection "Device fabrication" or "Surgical procedures" (lines 641-645).

Paragraph reads "Under sterile conditions, a 4-cm midline thoracolumbar incision and muscle dissection exposed three spinal levels. Device widths (1.7 mm for two-row, 1.4 mm for one-row) were designed to fit between adjacent dorsal root exit zones. A two-level laminectomy and pedicle resection opened the ventral epidural plane, into which a 7-0 polypropylene suture guided the array circumferentially around the cord. The device was rotated into orientation, tacked dorsally with silicone sealant (Kwik-Sil), and a grounding wire affixed to paravertebral muscle. The flexible flat cable was externalized via a vascular port into a subcutaneous pocket with controlled slack and dual 90° bends to relieve strain; the port was sutured to paraspinal musculature and the skin closed."

-In that regard, line 122 "...high-density microelectrode arrays that capture neural activity simultaneously across all spinal regions during naturalistic behavior." should be adapted.
The decoding of spinal locomotor control seems not specific. It was unclear throughout the manuscript if segmental spinal circuitries (e.g. CPG), descending motor tracts such as the CST / reticulospinal system, propriospinal projections or even sensory integration are assessed.

AUTHOR'S RESPONSE:
We agree that our device captures activity from all regions of the spinal cord cross-section (dorsal, ventral, lateral) at the implanted thoracolumbar level, rather than implying coverage of all rostro-caudal spinal segments. Regarding the source of recorded signals: our surface recordings likely reflect a mixture of segmental spinal circuitry (including CPG-related interneurons), descending motor tracts (corticospinal, reticulospinal), propriospinal projections, and sensory integration. The compound field potential signals represent ensemble activity from multiple neuronal populations simultaneously. Our decoding approach leverages this mixed-source signal for functional prediction rather than isolating individual circuit components.

MANUSCRIPT MODIFICATION:
The following sentence has been added to the Discussion: "Surface recordings at the thoracolumbar level likely reflect ensemble activity from multiple co-active neuronal populations, including segmental CPG circuitry, descending corticospinal and reticulospinal tracts, propriospinal projections, and sensory integration networks; our decoding approach leverages this compound signal for functional prediction rather than isolating individual circuit contributions."
Location in revised manuscript: Discussion, paragraph beginning "By targeting the thoracolumbar cord..." (lines 388–392).

Specific comments
Abstract: the term "autonomic modalities" is vague and does not represent the findings. The modality was two times sensory stimulation due to balloon inflation (urethra vs. sphincter).

AUTHOR'S RESPONSE:
The porcine experiment involved discrimination of visceral afferent stimuli (urethral and rectal sphincter balloon distension), representing sensory input from autonomic-relevant structures rather than efferent autonomic control.

MANUSCRIPT MODIFICATION:
We have revised the Abstract to state "visceral sensory inputs" instead of "autonomic modalities."
Location in revised manuscript: Abstract (line 71).

-The seminal studies on improved upper limb (https://doi.org/10.1038/s41591-024-02940-9) and hemodynamic functon(doi: 10.1038/s41591-025-03614-w, doi: 10.1038/s41586-020-03180-w) using epidural stimulation by the Courtine group are missing in the introduction (e.g. line 107).

AUTHOR'S RESPONSE:
We have added citations to the Courtine group's seminal work on epidural stimulation for upper limb recovery and hemodynamic function to the Introduction.

MANUSCRIPT MODIFICATION:
Location in revised manuscript: Introduction (lines 112-113).

-Figure 2d: in figure legend SNR is stated to be significantly different between movement and non-movement conditions. Please label accordingly in Figure.

AUTHOR'S RESPONSE:
We have added significance indicators to Figure 2d to clearly mark the statistically significant difference in SNR between movement and non-movement conditions.
Location in revised manuscript:

MANUSCRIPT MODIFICATION:
Figure 2d

Figure 3b is unclear. Do the authors herewith claim that only ascending and descending projections were assessed or also grey matter neurons (somata, missing in this figure)? This goes in the same direction as my specificity question above: which neurophysiological signals are measured (signaling along long projection neurons, segmental neuronal circuits)?

AUTHOR'S RESPONSE:
We thank the reviewer for this question. Figure 3b is a schematic illustration showing the anatomical organization of major ascending and descending white matter tracts around the spinal cord circumference. The cross-correlation velocity analysis presented in Figure 3 is designed to detect propagating signals within these white matter tracts (compound action potentials traveling rostro-caudally along axonal pathways).
We do not claim to directly record somatic activity from gray matter neurons. While volume conduction from gray matter sources cannot be entirely excluded, the metrics (RMS) and frequency band used for this analysis and the observed directional propagation velocities are most consistent with white matter tract activity. While we cannot claim with certainty that the detected signals belonged exclusively to long-projecting spinal tracts, the distinct directional patterns we were able to record, with sustained afferent versus efferent dominance that mapped systematically to anatomical tract locations around the cord circumference (Fig. 3e), support this interpretation.
Definitive determination of whether gray matter neuronal somata contribute to the recorded signals would require simultaneous surface and penetrating electrode recordings, which is

beyond the scope of the current study. We have clarified in the figure legend that the schematic depicts white matter tract organization relevant to the directional analysis.

MANUSCRIPT MODIFICATION:
Location in revised manuscript: Legend Figure 3 (lines 527).
"Figure 3b legend now reads.'"Electrode pairs positions (1-16) mapped around the spinal cord circumference showing anatomical orientation relative to major white matter tracts**"**

Line 404: "…making rostral spinal sampling a logical extension of necessary procedures...". Please explain in more detail. Necessary for what?

AUTHOR'S RESPONSE:
We have clarified that "necessary procedures" refers to the spinal decompression and stabilization surgeries routinely performed as part of acute SCI management.

MANUSCRIPT MODIFICATION:
Location in revised manuscript: Discussion (lines 428-430).
Paragraph now reads "….. undergo spinal surgery (e.g, decompression and/or stabilization) as part of acute injury management, making rostral spinal sampling a logical extension of these clinically necessary procedures rather than additional brain interventions …...."

Reviewer #3:

General Summary
The manuscript describes a thin flexible electrode array designed to wrap around the circumference of the spinal cord. The authors demonstrate the device's ability to record neural activity in freely moving rats over 3 days and to validate its scalability in acute porcine models. This work positions the implant design as a "middle ground" between non-invasive epidural leads, which lack spatial resolution and circumferential coverage, and penetrating intraspinal arrays, which risk tissue damage. The authors frame the device as a "multifunctional neuroprosthetic hub" capable of bidirectional interfacing.
The work is a noteworthy proof of concept in spinal neural interfaces with the demonstration of motor intent, sensory and autonomic decoding, and will be of interest to those in the field. However, the claim of chronic viability, and the lack of neurological deficit or tissue injury in larger animal models, are not sufficiently substantiated and should be paired back.

Comments to the Authors
1. The data presented does not support the claim that the implant is chronically viable. In rats, the device was tested for only 3 days, and in pigs, it was tested only under anaesthesia. Why did the authors not evaluate implant performance, deficits, etc, for longer periods in rats, and in an awake/walking pig?

AUTHOR'S RESPONSE:
We agree that three days does not constitute chronic implantation. As noted in our response to Reviewer 1, we have replaced "chronic" with "freely moving" throughout the manuscript to accurately reflect the implantation duration. The three-day limitation was imposed by UK Home Office licensing and housing constraints; our project license did not permit single housing, and group housing resulted in cage mates damaging the externalized connector through chewing behavior. Longer-term device viability in freely moving rats was demonstrated in our previous work, which included six-week dummy device implantation studies confirming stable tissue-electrode interaction and absence of neurological deficit (ref 15). The porcine experiments were designed exclusively as acute proof-of-concept demonstrations of anatomical scalability and decoding feasibility, and we have moderated all associated claims accordingly.

MANUSCRIPT MODIFICATION:
"Freely moving" replaces "chronic" throughout (Abstract, Introduction, Results, Discussion).
Porcine findings reframed as proof-of-concept demonstrations of scalability.

2. Why was there no evaluation of the foreign body response? Long-term implantation (weeks to months) is likely to generate significant FBR, which could limit the implant's ability to conform to mobile spinal tissue during movement, particularly in larger animals where spinal motion is greater than in a rat.

AUTHOR’S RESPONSE:
We agree that foreign body response (FBR) evaluation is critical for long-term viability. Comprehensive histological assessment of tissue response was performed in our previous work (1), which included six-week dummy device implantation studies in freely moving rats demonstrating no spinal cord compression, tissue damage, or significant inflammatory response. However, that prior work did not include electrophysiological recordings in freely moving animals or functional decoding, which are the primary focus of the present study. The

current work therefore complements the earlier biocompatibility assessment by demonstrating stable electrophysiological performance during recovery-period implantation.
We fully acknowledge that chronic FBR evaluation in larger animals (pigs, non-human primates) under conditions of greater spinal motion represents an essential next step for translational validation and should be performed in relevant models prior to clinical deployment.

(1) doi: 10.1126/sciadv.adl1230

3. Can the authors elaborate on the risk profile for a circumferential implant design in a chronic model compared to a dorsal implant? How does the device design account for swelling, or dural scarring that might lead to compressive myelopathy over months/years? The authors could establish the necessity of the circumferential design by comparing decoding performance using only the dorsal subset of electrodes.

AUTHOR'S RESPONSE:
We agree that a circumferential design introduces a theoretical long-term risk of compressive myelopathy if dural scarring or fibrotic encapsulation progressively reduces the subdural space. Such delayed compression has been reported around epidural neuromodulation hardware in rare cases, supporting the importance of long-term surveillance in chronic implants. (1)
That said, the magnitude of chronic foreign-body response is strongly influenced by mechanical micromotion and effective stiffness at the tissue–implant interface (2). Our arrays are ultrathin (parylene-C Young's modulus ~2.8 GPa) and therefore have very low bending stiffness (scaling with E·h^3), which reduces local tissue deformation compared with thicker conventional implants; silicon-based components have substantially higher modulus (~130–170 GPa)(3). Spinal cord tissue mechanics are in the kPa range by indentation-based measurements (with method-dependent variability), underscoring why geometry-driven compliance is critical (4).

Future iterations could further mitigate chronic encapsulation risk by incorporating tissue-like hydrogel/silicone coatings or other anti-fibrotic surface strategies shown to reduce foreign-body reaction (5). We also agree that extended-duration studies (months) in large-animal models are required to quantify chronic risk and confirm absence of delayed compression (6).

Finally, regarding the necessity of circumferential coverage, we note that the manuscript already includes a channel-level importance analysis that directly addresses this point. Specifically, the per-electrode ablation/importance map (Fig. 4d, Supplementary Figs. 10–11) shows that the most informative electrode–frequency features are distributed around the cord, with a predominant contribution from ventral and lateral sites, while some dorsal electrodes remain informative.
This indicates that high decoding accuracy is not driven solely by dorsal recordings; rather, it depends on access to signals captured outside the dorsal domain. Therefore, a dorsal-only configuration would remove a substantial fraction of the highest-value inputs identified by the model, and would be expected to reduce peak decoding performance relative to the full circumferential array.

Regarding the necessity of circumferential coverage, the reviewer raises an important point that we believe other readers may also consider. We note that the manuscript already includes a channel-level importance analysis (Fig. 4d, Supplementary Figs. 10–11) that directly addresses this question. To ensure this analysis is more clearly highlighted for readers, we have expanded the relevant Discussion paragraph to emphasize that the per-electrode ablation/importance map demonstrates that the most informative electrode–frequency features are distributed around the

cord, with predominant contributions from ventral and lateral sites, while some dorsal electrodes remain informative.
This spatial distribution indicates that high decoding accuracy is not driven solely by dorsal recordings; rather, it depends on access to signals captured outside the dorsal domain. Therefore, a dorsal-only configuration would remove a substantial fraction of the highest-value inputs identified by the model and would be expected to reduce peak decoding performance relative to the full circumferential array. We thank the reviewer for highlighting this point, as it underscores a key functional advantage of the circumferential design that may benefit from greater emphasis in the manuscript.

(1) doi:10.3171/2009.10.SPINE09193
(2) doi:10.3389/fbioe.2021.622524
(3) doi:10.1002/jbm.b.31584
(4) doi:10.1089/ten.teb.2010.0520
(5) doi:10.1101/829648
(6) doi:10.4403/1673-5374.380895

MANUSCRIPT MODIFICATION:
The following has been added to the Discussion paragraph on circumferential coverage: *"The per-electrode ablation and importance analyses (Fig. 4d, Supplementary Figs. 10–11) demonstrate that the most informative features are distributed around the cord circumference, with predominant contributions from ventral and ipsilateral electrodes. This spatial distribution underscores the functional necessity of circumferential coverage, as a dorsal-only configuration would exclude substantial predictive information from non-dorsal territories."*
Location in revised manuscript: Discussion, paragraph beginning "Building on these recordings..." (lines 401–406).

4. The authors state, “no neurological deficits or tissue injury attributable to the implant.” How was neurological deficit quantified? The presence of deficits in hind limb function, assessed with the BBB scale or similar, should be provided. Similarly, how was tissue injury quantified? Histology, showing the absence of compressive injury and the absence of an inflammatory response in the spinal cord, should be presented. How was the lack of deficit or tissue injury assessed in the pig models?

AUTHOR’S RESPONSE:
We agree that the original statement lacked methodological detail. As noted in our response to Reviewer 1, we have removed this sentence from the manuscript.
Rodent neurological assessment: In the current study, animals were observed daily for gross motor deficits (dragging, paresis, or paralysis of hindlimbs) and alterations in gait during open-field locomotion used for kinematic recordings. No overt deficits were observed over the three-day recovery period. However, we did not perform standardized hindlimb function scoring (e.g., BBB scale) or terminal histological examination in these animals, as comprehensive tissue response characterization was conducted in our previous work (1) using six-week dummy device implants, which demonstrated absence of spinal cord compression, axonal damage, or significant inflammatory infiltration.

Porcine assessment: Pigs were implanted under terminal anesthesia and euthanized at the conclusion of electrophysiological recordings (2–4 hours post-implantation). No histological or

functional assessment was performed. The statement regarding absence of injury in pigs was not supported by data and has been removed.

(1) doi: 10.1126/sciadv.adl1230

MANUSCRIPT MODIFICATION:
Location in revised manuscript: Sentence removed from Results.

5. Was the decoding model tested on new animals that were not part of the training set (leave-one-out cross-validation), or only on held-out temporal data from the same animals? If the latter, the high accuracy may reflect overfitting to the specific gait idiosyncrasy of those five rats rather than a generalisable decoder of spinal motor control.

AUTHOR'S RESPONSE:
The decoder was trained on pooled data from all five rats and tested on temporally held-out data from these same animals (train/test split within each recording session). We did not perform leave-one-animal-out cross-validation.

We acknowledge that testing on held-out temporal data from the training animals does not fully exclude the possibility of overfitting to individual gait characteristics. However, several factors support the generalizability of the decoding approach:

1. The model was trained on combined data from five rats with varied gait patterns, body weights, and individual motor strategies, rather than being optimized for a single animal.
2. Multiple independent analyses (ablation, SHAP, Random Forest feature importance, mutual information) all identified the same delta-band (1–4 Hz) features as most predictive (Fig. 4c, Supplementary Figs. 9–12), suggesting the model leveraged generalizable biomechanical principles (central pattern generator rhythms) rather than animal-specific idiosyncrasies.
3. Performance remained stable across different phases of each recording session, indicating the model generalized across time within animals.

We note that inter-individual variability in locomotor patterns; arising from differences in body morphometry, muscle strength, gait preferences, and spinal circuitry organization, is substantial even among genetically similar laboratory rats. Importantly, in real neuroprosthetic or brain–computer interface applications, decoders are not deployed as fixed models trained exclusively on other individuals. Rather, models are calibrated and, when necessary, fine-tuned using each subject's own neural and behavioral data to account for subject-specific physiology and neural representations. This subject-specific adaptation is standard practice across both invasive and non-invasive BCI systems and is essential for achieving optimal performance. Accordingly, the primary goal of the present study was to demonstrate that spinal cord surface recordings contain sufficient information to support high-fidelity motor decoding when appropriately trained, rather than to establish cross-animal generalization of a single universal decoder.

In this context, our goal was to demonstrate that spinal cord surface recordings contain sufficient information to support high-fidelity motor decoding when appropriately trained. The convergence of feature importance across independent analytical methods and the reliance on low-frequency rhythms consistent with established CPG dynamics suggest that the decoding strategy is grounded in generalizable physiological principles, even though optimal performance would require individualized model training in clinical deployment.

MANUSCRIPT MODIFICATION:
N/A

6. The authors presented confusion matrices for pig sensory and autonomic decoding separately. Were these separate models? The authors should address potential multimodal interference (misclassification between sensory/autonomic stimuli and motor control), as would be required in a clinical application.

AUTHOR'S RESPONSE:
These were separate models trained independently on distinct experimental paradigms. We acknowledge the importance of testing multimodal interference; specifically, whether models can discriminate between sensory inputs, visceral afferent activation, and ongoing motor activity within a single recording session, as would be required for closed-loop clinical neuroprosthetics.

We initially considered conducting multimodal testing in the anesthetized porcine preparation but determined that awake, freely moving animals provide a more rigorous and clinically relevant testbed for this challenge. To address this, in the rodent sensory decoding experiments (Fig. 5a–b), we deliberately performed sensory testing while animals were freely ambulatory. The model successfully discriminated evoked sensory responses in the presence of spontaneous locomotor activity. In our view, this represents a form of multimodal interference testing, albeit not systematically varied. Awake preparations are inherently more demanding: they include spontaneous neural activity, ongoing postural adjustments, and sensory feedback from naturalistic movement, all of which increase background signal complexity compared to anesthetized states where neural systems are pharmacologically suppressed. The porcine experiments were designed as proof-of-concept demonstrations of cross-species scalability and device feasibility at larger anatomical scales, not as definitive multimodal classification studies. We agree that systematic multimodal decoding (training a single classifier to distinguish motor intent, sensory stimuli, and visceral afferent activation simultaneously) is an essential next step and should be performed in awake large-animal models prior to clinical translation. We have revised the text to clarify that sensory and visceral decoding used separate models, to appropriately frame the porcine work as scalability demonstrations, and to explicitly state that rodent sensory testing was performed during ambulatory behavior to introduce multimodal complexity.

MANUSCRIPT MODIFICATION:
We then turned to visceral afferent decoding using an independent model, trained separately from the sensory classification network on a distinct experimental paradigm, which provides autonomic-relevant information for bladder and bowel function monitoring, as an exploratory proof-of-concept demonstration. In a single porcine experiment, an independent network achieved near-perfect classification of rest, urethral sphincter distension, and rectal sphincter distension ($F1 \geq 0.97$, $n = 1$), with virtually no cross-class confusion (Fig. 5d; Supplementary Figs. 20–22). These results demonstrate that even subtle physiological states, previously inaccessible to spinal surface recordings, can be discriminated with high fidelity.

Location in revised manuscript: Results, "Neural decoding differentiates sensory and autonomic responses across species" subsection (lines 312-317); clarified in figure legends (Fig. 5c–d, line 585).

Minor Comments

7. The authors state the implant can “capture neural activity simultaneously across all spinal regions”. This needs clarification - presumably, neural activity was only captured from regions under the implant. For the rodent model, it is not stated which spinal segment the implant was located over.

AUTHOR’S RESPONSE:

We agree this phrasing was ambiguous. As clarified in our response to Reviewer 2, "all spinal regions" refers to all territories of the spinal cord cross-section (dorsal, ventral, and lateral surfaces) at the implantation level, not to all rostro-caudal segments.

In rodents, devices were implanted at the thoracolumbar junction , as now explicitly stated in both the Results and Methods sections. This location was selected to access lumbar motor circuitry, including central pattern generators for hindlimb locomotion, while avoiding the cervical and lumbar enlargements where increased cord diameter and root density complicate implantation.

MANUSCRIPT MODIFICATION:

We have revised the manuscript to explicitly state the implantation level in the Results text and to clarify that circumferential coverage refers to the spinal cord cross-section at this anatomical location.

Location in revised manuscript: Results, "Scalable circumferential designs safely validate across species" subsection (line 148); Methods, "Device implantation (rodents)" (lines 641-645).

8. For sensory decoding, why did the authors not perform negative controls, e.g. sensory stimuli at the forelimbs, which would not be expected to be detected lower in the spinal cord?

AUTHOR’S RESPONSE:

We agree that forelimb sensory stimulation would have provided an anatomically grounded negative control to confirm that signals detected at the thoracolumbar level arise from hindlimb/tail territories rather than volume-conducted or non-specific activity. We did not include this control in the current study.

However, we note that the experimental design included rest periods with no sensory stimulation, which served as a baseline class in the decoding model (Fig. 5a–b, Supplementary Figs. 14–15). The classifier successfully discriminated rest from all sensory modalities, demonstrating that the model does not spuriously classify activity during non-stimulation epochs as sensory input. Additionally, the confusion matrix (Fig. 5b) shows that classification errors were confined to stimuli of the same modality applied to different limbs or intensities, consistent with overlapping somatotopic representation within adjacent spinal segments.

While rest-state baselines partially address specificity, we acknowledge that forelimb stimulation would provide a more rigorous negative control by testing whether the decoder inappropriately responds to afferent signals outside the anatomical territory sampled by the device. This represents a valuable addition for future validation studies.

MANUSCRIPT MODIFICATION:

N/A

9. “rostro–caudal signal propagation, a feature inaccessible to conventional planar arrays.” This statement needs clarification. Also note recent work by Hazelgrove et al. (2025, Scientific Reports), which reported the propagation of neural spikes in a dorsal planar array.

AUTHOR’S RESPONSE:
We thank the reviewer for bringing this recent work to our attention. Hazelgrove et al. (2025, Scientific Reports) was not available during drafting. This work demonstrates the detection of propagating neural spikes along a dorsal planar array in freely moving rodents, which represents an important methodological advancement.
We have revised the statement to acknowledge this recent work and to clarify our claim. Our two-row configuration enables simultaneous sampling of rostro-caudal propagation across dorsal, lateral, and ventral territories of the spinal cord circumference (Fig. 3b,e), providing spatial information about directional bias that varies systematically around the cord cross-section. Additionally, while Hazelgrove et al. rely on spike detection, which requires high signal-to-noise ratio and may not capture all underlying spinal activity, our cross-correlation velocity analysis uses ensemble field potentials (RMS amplitude across the full frequency band), thereby capturing propagating activity across a broader range of signal amplitudes and neural populations. This circumferential perspective complements the dorsal planar approach demonstrated by Hazelgrove et al. by capturing propagation signals from ventral and lateral white matter tracts in addition to the dorsal columns.

MANUSCRIPT MODIFICATION:
We have added this citation and tempered the language accordingly.
Location in revised manuscript: Results, "Two-row arrays resolve directional signal flow within spinal circuits" subsection (lines 205-206); Discussion (lines 384); reference added to reference list.

10. “Electrode impedances remained stable before and after implantation”. While there is no significant difference on a population level, the reported impedance values extend across orders of magnitude from 1 kΩ to 100 MΩ. What are these impedances? |Z| at 1 kHz? Why is there such a large range in the measured values? Methods should include input parameters for impedance measurement/configuration.

AUTHOR’S RESPONSE:
Impedance measurements were obtained at 1 kHz using an AutoLAB potentiostat in phosphate-buffered saline with platinum counter and reference electrodes, as now detailed in Methods.
The reviewer correctly identified that impedance values span multiple orders of magnitude. Measured impedances ranged from 0.22 kΩ to 125 MΩ (125,000 kΩ) across all electrodes. This wide range might arise from PEDOT:PSS coating thickness variations, microscale fabrication geometry differences, and variability in tissue-electrode contact area and possibly to electrode damage while handling and before and after implantation.
We have added Supplementary Figure 4, which shows electrode-by-electrode impedance measurements for all 15 implanted devices ($n = 480$ paired electrodes). Individual electrodes maintained stable impedance characteristics across implantation, with no significant population-level change ($p = 0.412$), indicating that most electrodes preserved their electrical characteristics despite surgical implantation.

Functional analyses (motor decoding, sensory classification) were performed on recordings with adequate signal quality, as determined by signal-to-noise ratio during experimental sessions

Location in revised manuscript: Methods, Impedance Measurements (lines 643–645); Supplementary Figure 4 added.

MANUSCRIPT MODIFICATION:
Methods section updated to specify that impedance measurements were obtained at 1 kHz using an AutoLAB potentiostat in phosphate-buffered saline with platinum counter and reference electrodes. Supplementary Figure 4 added showing electrode-by-electrode impedance measurements for all 15 implanted devices (n = 480 paired electrodes).
Location in revised manuscript: Methods, Impedance Measurements (lines 616-618); Supplementary Figure 4 added.

Reviewer #3:

No code review

AUTHOR'S RESPONSE:
Code is provided with resubmission

Manuscript Additions
Code provided.

In addition to the revisions described above, we have included the following new supplementary item in the revised submission:

Supplementary Movie 1 | Neural decoding of locomotor kinematics from circumferential spinal recordings, showing side-by-side comparison of ground truth DeepLabCut trajectories versus spinal electrode-derived decoder predictions in a freely moving rat.

RESPONSE TO REVIEWERS

We have implemented the following color-coding system to respond to the reviewers' comments and explain the related changes in the manuscript:

REVIEWERS' COMMENTS: Original Comment
AUTHOR'S RESPONSE: Answer to each point
MANUSCRIPT MODIFICATION: Additions to the manuscript based on the addressed point

AUTHOR'S RESPONSE: We thank the Editor and Reviewers for their careful evaluation and feedback. We have revised the manuscript to clarify the remaining points raised by reviewer one and toned down the manuscript to reflect the presented results and accurately mirror the evidence.

REVIEWERS' COMMENTS

Reviewer #1 (Remarks to the Authr):

The authors have provided extensive responses to the reviewer comments, and these efforts are appreciated. The revisions partially rely on rephrasing existing text rather than adding new data to substantiate the claims. As a result, some conclusions remain stronger than what is directly supported by the evidence, and some statements still read more like speculative points for future discussion than evidence-based findings. I outline below my comments, following the same numbering as in the initial request:

1) to address the comment, the authors have revised the text to include two lines "Building on our previously established device concept and framework..." and "...recordings during naturalistic behavior in freely moving animals." While this is appreciated, the manuscripts novelty as presented still appears to shift between the underlying technology and the current experimental extension. Given that the core technology has already been described in a previous publication, a more substantial revision of the overall narrative would be preferable rather than limited rephrasing in the main text.

We thank the reviewer for this feedback. We have revised the final paragraph of the Introduction to explicitly delineate what was established in our prior publication from what the present study contributes. The revised text now opens by summarizing the prior work, then enumerates three specific directions that are new to the present study, and closes with a forward-looking statement that avoids overstating translational readiness.

MANUSCRIPT MODIFICATION
" Our prior work established the circumferential intradural recording concept: flexible thin-film arrays capable of 360-degree spinal cord coverage, with proof-of-concept recordings in anesthetized rodents, tissue-response assessment using dummy implants, and cadaveric human feasibility studies . The present study extends this platform in three directions not

previously demonstrated: stable recordings during short-term implantation (up to three days) in freely moving rats; high-fidelity motor intent extraction ($R^2 = 0.97$) and multimodal, plurisegmental sensory classification (94.4% accuracy) during naturalistic behavior; and cross-species scaling to porcine models with proof-of-concept visceral afferent discrimination. These advances position circumferential spinal recording as a functionally characterized platform for multidomain spinal decoding, identifying a path toward future closed-loop neuroprosthetic applications."

Location in revised manuscript: Introduction, final paragraph (lines 89–99).

2) The authors note that 3 days does not represent a chronic experiment. However, the manuscript does not clearly identify the study as acute. Instead, the term “chronic” have been replaced in some places with “freely moving” or “recovery.” The manuscript should explicitly state in the abstract, main text, and discussion that this is concerns an acute experiment (3 days in freely moving rats, anesthetized porcine models). This information is currently mentioned only in the Results section.

Additionally, the manuscript should address why the study was limited to 3 days, or whether this was due to animal care considerations, device failure, housing constraints, or other factors. This point remains unaddressed.

We thank the reviewer for this important point. We agree that the implantation duration and its basis should be explicitly stated throughout the manuscript.

We have made the following additions:

Abstract: "In freely moving rats" has been revised to "In freely moving rats during short-term implantation (up to three days)" and "In pigs, cross-species validation confirmed translational scalability" has been revised to "In acutely anaesthetized pigs, cross-species proof-of-concept experiments confirmed anatomical scalability."

Introduction: The final paragraph now reads "stable recordings during short-term implantation (up to three days) in freely moving rats."

Results: The section heading has been revised to "Short-term freely moving recordings reveal locomotor signatures" and the text now reads "confirming that the platform supports short-term recovery spinal electrophysiology."

Methods: The following statement has been added to the In vivo experiment section: "Implantation duration was limited to three days by UK Home Office project license constraints (PFF2068BC), which precluded single housing; group-housed animals with externalized connectors were susceptible to cage-mate interference and thus unnecessary harm. Porcine implantations were performed under terminal anesthesia as acute proof-of-concept demonstrations of anatomical scalability."

MANUSCRIPT MODIFICATION

Location in revised manuscript: Abstract (lines 33-34 & 36-37); Introduction, final paragraph (line 93); Results, section heading and paragraph (lines XXX–XXX); Methods, "In vivo experiment" section (lines 132 & 139).

3) The authors argue " we note that across all implanted animals, no neurological deficits or gross tissue injury were observed during the study (3 days), as evidenced by animals resuming normal ambulatory behavior upon recovery" and to address the comment they revised the text to " Across all cases, we observed no neurological deficits or tissue injury attributable to the implant during the duration of the study (3 days). (lines 163–164).
The authors' statement that animals resumed normal ambulatory behavior upon recovery is presented without accompanying objective evidence. In the absence of histological analysis, quantitative neurological scoring, or direct assessment of tissue compression and edema, these observations remain insufficiently substantiated. As a result, the statements regarding safety and any implications for scalability are not adequately supported by the data presented. Additional quantitative evaluations would be necessary to justify these claims.

We thank the reviewer for this important point. We have removed the claim regarding absence of neurological deficits and tissue injury from the manuscript. In its place, we have included a factual observational statement referencing Supplementary Movie 1, which documents ambulatory behavior in freely moving rats following recovery from anesthesia, and directing readers to our prior work for comprehensive tissue-response characterization conducted using six-week dummy implants in freely moving rats.

MANUSCRIPT MODIFICATION
The revised text reads:
"All animals resumed spontaneous ambulatory behavior following recovery from anesthesia, as documented in Supplementary Movie 1. Tissue-response characterization was conducted in our prior work [15]."

Location in revised manuscript: Results, "Scalable circumferential designs safely validate across species" (lines 128–130).

4) The authors provided the details on measurement frequency, instrument, and electrolyte setup, and add supplementary electrode-level impedance data.
The explanation for the large spread in the data remains somewhat speculative. While the authors list several possible causes, they do not clarify whether or how this variability affects performance over the implantation period. Although variability in impedance is not unusual, the magnitude of the observed spread may raise questions regarding the reproducibility of the electrode fabrication process.

We thank the reviewer for this thoughtful observation and agree that the sources of impedance variability warranted further clarification. Wide impedance distributions in PEDOT:PSS-coated electrode arrays are a well-documented characteristic of this material system, arising from microscale coating thickness variations and fabrication geometry differences across large arrays. We acknowledge that the magnitude of the

spread observed here is substantial and that tighter fabrication process control represents an important objective for future device iterations.
Critically however, this variability did not compromise functional recording performance in the current study. Animals yielded recordings with adequate signal quality (SNR: 33.7 ± 9.4 dB), and population-level impedance remained stable across the implantation period (two-sided paired t-test, $t(14) = 0.86$, $p = 0.412$), confirming that the observed variability did not worsen over time and did not adversely affect the electrophysiological data used for all decoding analyses reported in this manuscript.

No changes were made to the manuscript for this comment.

5) no further comment.

6) no further comment.

7) no further comment.

8) The authors have moderated the language and now present the porcine findings as proof-of-concept. However, the manuscript still advances the translational pathway (and related discussions)relatively strongly, despite the limited porcine sample size (and the use of anesthetized animals).

We thank the reviewer for this continued feedback. We agree that the porcine findings should be consistently framed as proof-of-concept demonstrations given the limited sample sizes (n = 1–2) and use of anesthetised animals. We have carefully reviewed the Discussion and made the following targeted modifications to confirm that the language accurately reflects the nature of the evidence.

MANUSCRIPT MODIFICATION

1. "Validation in pig models showed successful scaling of sensory decoding capabilities" has been revised to "Validation in pig models showed promising scaling of sensory decoding capabilities."
2. "Porcine intraspinal source-localization experiments achieved reliable discrimination" has been revised to "porcine intraspinal source-localization experiments demonstrated discrimination."
3. "This anatomical complementarity represents a paradigm shift from current brain-centric approaches" has been revised to "This anatomical complementarity represents an alternative approach to current brain-centric strategies."

Location in revised manuscript: Discussion (lines 375 & 384-385 & 391 & 407).

9) no further comment.

10) no further comment.

11) no further comment.

Minor comments

Minor 1) The authors state that Supplementary Figure 4 demonstrates electrode-level stability across implantation with no significant population-level change. However, I am not convinced how Figure 4 (suppl.)help supporting this claim. As currently presented, the figure appears to be a collection of data points plotted together without clear structure, making it difficult to interpret the intended message. I encourage the authors to reconsider the presentation of this figure, ensuring that it conveys the key result and supports the stated conclusion.

We thank the reviewer for this feedback. We agree that the figure legend did not sufficiently guide interpretation of the visual. We have substantially revised the legend to clarify the structure: each grey line represents a single electrode connecting its pre- and post-implantation impedance value on a log scale. The overlapping appearance is intentional and reflects the full dataset of 480 electrode pairs plotted simultaneously to maintain complete transparency. The key message ; no significant population-level change across implantation , is directly supported by the statistical summary now included in the legend.

MANUSCRIPT MODIFICATION
Supplementary Figure 4 | Electrode-by-electrode impedance stability. Paired impedance measurements at 1 kHz for all 15 implanted rodent devices (32 channels per animal, n = 480 electrodes total, 15 biological replicates). Each grey line connects the pre- and post-implantation impedance values of a single electrode on a log scale (kΩ). The overlapping appearance reflects the full dataset plotted simultaneously to maintain transparency across all 480 electrode pairs. The population-level analysis shows no significant change across implantation which is supported by a two-sided paired t-test ($t(14) = 0.86$, $p = 0.412$, Cohen's $d = 0.29$).
Location in revised manuscript: Supplementary information (page4).

Minor 2) no further comments.
Minor 3) no further comments.

Reviewer #2 (Remarks to the Author):

The authors did a great job in addressing my comments and apadpted the manuscript accordingly. With the additionally provided information the article improved clarity.

Reviewer #3 (Remarks to the Author):

The authors have addressed all questions raised in the peer review, and publication is recommended

Reviewer #4 (Remarks to the Author):

I co-reviewed this manuscript with one of the reviewers who provided the listed reports. This is part of the Nature Communications initiative to facilitate training in peer review and to provide appropriate recognition for Early Career Researchers who co-review manuscripts.
